# Proteomic Analysis of INS-1 Rat Insulinoma Cells: ER Stress Effects and the Protective Role of Exenatide, a GLP-1 Receptor Agonist

**DOI:** 10.1371/journal.pone.0120536

**Published:** 2015-03-20

**Authors:** Mi-Kyung Kim, Jin-Hwan Cho, Jae-Jin Lee, Moon-Ho Son, Kong-Joo Lee

**Affiliations:** 1 Graduate School of Pharmaceutical Sciences and College of Pharmacy, Ewha Womans University, Seoul 120–750, Republic of Korea; 2 Dong-A ST Research Institute, Yongin-si, Gyeonggi-do 446–905, Republic of Korea; Indiana University School of Medicine, UNITED STATES

## Abstract

Beta cell death caused by endoplasmic reticulum (ER) stress is a key factor aggravating type 2 diabetes. Exenatide, a glucagon-like peptide (GLP)-1 receptor agonist, prevents beta cell death induced by thapsigargin, a selective inhibitor of ER calcium storage. Here, we report on our proteomic studies designed to elucidate the underlying mechanisms. We conducted comparative proteomic analyses of cellular protein profiles during thapsigargin-induced cell death in the absence and presence of exenatide in INS-1 rat insulinoma cells. Thapsigargin altered cellular proteins involved in metabolic processes and protein folding, whose alterations were variably modified by exenatide treatment. We categorized the proteins with thapsigargin initiated alterations into three groups: those whose alterations were 1) reversed by exenatide, 2) exaggerated by exenatide, and 3) unchanged by exenatide. The most significant effect of thapsigargin on INS-1 cells relevant to their apoptosis was the appearance of newly modified spots of heat shock proteins, thimet oligopeptidase and 14-3-3β, ε, and θ, and the prevention of their appearance by exenatide, suggesting that these proteins play major roles. We also found that various modifications in 14-3-3 isoforms, which precede their appearance and promote INS-1 cell death. This study provides insights into the mechanisms in ER stress-caused INS-1 cell death and its prevention by exenatide.

## Introduction

Recent reports suggest that hyperglycemia results in both oxidative and endoplasmic reticulum (ER) stresses [1], suggesting that ER stress and oxidative stress are endogenous aggravating factors for type 2 diabetes. ER stress is a cellular state involving accumulation of unfolded proteins, perturbation of calcium ions, or disturbances of redox state [2,3]. Inhibitors of ER stress might therefore serve as antidiabetic agents. Thapsigargin, a selective inhibitor of endoplasmic reticulum Ca^2+^/ATPase causing the depletion of ER Ca^2+^ store, is widely used in studies of ER stress-caused beta cell death. When cells fail to adapt to ER stress, beta cell apoptosis is initiated by diverse signaling molecules.

A progressive increase in beta cell apoptosis and decrease in beta cell mass in the pancreas, occur with the progression of type 2 diabetes. Because pancreatic beta cell loss cannot be readily recovered, there has been a heightened interest in agents such as exenatide that preserve beta cell mass in addition to lowering glucose. Exenatide, a dipeptidyl peptidase 4-resistant glucagon-like peptide (GLP)-1 analogue, has been approved for therapy of type 2 diabetes [4], because it effectively blocks beta cell death caused by various diabetogenic agents via cAMP-dependent, beta arrestin-mediated and PI3-kinase-mediated signaling pathways [5]. Recently, it has been reported that a component of beta arrestin-mediated signaling pathway, 14–3–3 scaffold protein, binds to the proapoptotic protein, BAD, and inhibits beta cell apoptosis [6]. 14–3–3 proteins are 28–30 kDa signaling proteins, that inhibit apoptosis by regulating over 200 partner proteins [7,8]. 14–3–3 proteins appear in seven isoforms (β, γ, ε, ζ, η, θ, and σ), but little is known on the modulation of these isoforms under stress conditions [9].

We previously reported that isoform-specific changes in 14–3–3 proteins are crucial to INS-1 rat insulinoma cell death caused by stress and that decreases in 14–3–3θ is a causative factor in INS-1 cell death [10]. We also suggested that exenatide prevents stress-induced INS-1 cell death by modulating 14–3–3 isoforms. Several questions remain in this regard: Are other entities in addition to 14–3–3 isoforms affected by ER stress?; what are the effects of GLP-1R signaling on these entities?; and what modifications in these entities are responsible for INS-1 cell apoptosis in ER stress and type 2 diabetes?

In efforts to answer these questions, we comprehensively examined the protein changes occurred after treatment with thapsigargin with and without exenatide treatment based on our previous extensive studies [10],. For this, we performed proteomics combined with two-dimensional gel electrophoresis (2D-PAGE) and mass spectrometry (MS) to identify the proteins changed by ER stress as well as during prevention or reversal of those changes by exenatide. We sorted the proteins up- or down-regulated by thapsigargin and are thus presumably involved in ER stress, into three groups, based how they were further altered by exenatide: 1) those whose altered regulations were reversed; 2) those whose altered regulations were exaggerated; 3) those whose altered regulations were unchanged. We concluded that proteins in groups 1 and 2, in which exenatide up- or down-regulated thapsigargin initiated alterations, may be involved in cell death, and those in group 3 not affected by exenatide may be products of ER stress, but not involved in prevention by exenatide. We identified the modified proteins in the three groups by mass spectrometry and computer assisted predictions. Based on the identities of the key proteins, we attempted to infer the signaling pathways activated by ER stress and how exenatide may have influenced these pathways to reverse the effects of ER stress.

## Materials and Methods

### Materials

Exenatide was obtained from American Peptide (Sunnyvale, CA, USA). Unless otherwise specified, the cell culture reagents were obtained from Invitrogen (Carlsbad, CA, USA), and the chemical reagents from Sigma-Aldrich (St. Louis, MO, USA).

### Cell culture

Rat insulinoma cell line, INS-1, was kindly provided by Prof. Kang of Ajou University, Suwon, Korea [11]. The cells were maintained in RPMI 1640 (11 mM glucose), supplemented with 10% fetal bovine serum, 1 mM sodium pyruvate, and 10 mM HEPES, at 37°C in a humidified atmosphere (5% CO_2_, 95% air). All experiments were performed with INS-1 cells between the 15^th^ and 23^rd^ passages.

### Glucose-stimulated insulin secretion

INS-1 cells were seeded at a density of 5 × 10^4^ cells per well in a 96-well plate, and allowed to grow for 48 h. After two washes with phosphate buffered saline, the cells were serum-deprived for 1 h in Krebs–Ringer bicarbonate HEPES (KRBH) buffer (11.5 mM NaCl, 24 mM NaHCO_3_, 5 mM KCl, 1 mM MgCl_2_, 25 mM HEPES, pH 7.4) including 1% bovine serum albumin (BSA), followed by treatment with exenatide in KRBH buffer containing 1% BSA, 11.1 mM glucose, and 100 μM Ro-201724 (Calbiochem, San Diego, CA, USA) for 0.5 h and centrifuged. The secreted insulin was measured in the supernatants using Ultrasensitive Rat Insulin ELISA kit (ALPCO, Windham, NH, USA). The cellular protein levels were measured using bichinchonic acid kit (Pierce, Rockford, IL, USA) after lysing the cells with 0.1 M hydrogen chloride. Insulin levels were presented as ng insulin/μg protein.

### INS-1 cell viability assay

INS-1 cells were plated at 7 × 10^4^ cells per well in a 96-well plate, and at 7.8 × 10^6^ cells in 150 mm dish, and incubated for 48 h in serum-deprived in RPMI media with 5.6 mM glucose. The resulting cells were maintained in serum-free, glucose (5.6 mM) containing media and then treated with 0.3 μM thapsigargin, to promote ER stress, and apoptosis [12], in the absence or presence of exenatide, for 6 h at 0.2% final dimethyl sulfoxide concentration. Cell viability was assessed by determining intracellular ATP levels with Celltiter-Glo reagent (Promega, Madison, WI, USA) using a luminometer (LmaxII384, MDC, Sunnyvale, CA, USA), according to the manufacturer’s instructions and expressed as percentages of untreated control.

### Caspase activity

This was assessed using Caspase-Glo 3/7 assay reagent (Promega Co., WI, USA) described as previously [10].

### RT-PCR

Total RNA was extracted using Trizol (Invitrogen, Carlsbad, CA). First strand cDNA was synthesized using M-MLV reverse transcriptase (Promega, Madison, WI, USA). PCR was performed for following rat target genes-thioredoxin interacting protein (TXNIP; Gene Bank Accession No. NM 001008767), insulin receptor substrate-2 (IRS-2; NM 001168633), immunoglobulin heavy chain binding protein (Bip, alternatively called GRP78; M14050), CCAAT/enhancer binding protein homologous protein (CHOP; U30186), glucagon-like peptide 1 receptor (GLP-1R; NM 012728) and beta actin (BC063166) in a Dyad Thermal Cycler (MJ Research, Watertown, MA). First strand cDNA was synthesized using M-MLV reverse transcriptase (Promega, Madison, WI, USA). Each set of primers was designed using Primer3 (http://bioinfo.ut.ee/primer3-0.4.0/primer3/). Primer sequences used, concentration of MgCl_2_, cycle number and annealing temperature for each target gene are listed in S1 Table. Images of PCR products were captured using BioCapt v1.01 software and densitometric analysis was performed with Bio1D v1.01 software (Vilber-Loumat, Marne la Vallée, France).

### Extraction of cellular proteins

Following harvest, the cells were frozen immediately and stored at −80°C until further use.

Soluble proteins were extracted from the cells as follows: Cells were solubilized with a lysis buffer containing 7 M urea, 2 M thiourea, 4% v/v CHAPS, 2% ampholine (1.5% for pH 3–10, 0.5% for pH 5–7) and 65 mM DTT. After centrifugation at 20,000 x g for 30 min at 25°C, the supernatants were left at room temperature for 1 h to ensure protein solubilization and denaturation. The protein concentrations of the final extracts were determined using a 2-D Quant Kit (GE Healthcare, Piscataway, NJ, USA).

### Protein separation using two-dimensional polyacrylamide gel electrophoresis

Samples of the cellular protein extracts prepared as described above were subjected to two dimensional-polyacrylamide gel electrophoresis (2D-PAGE). One hundred μg of each protein sample was loaded onto the strip gels and rehydrated for 12 h (18 cm, pH 4–7) with rehydration buffer (7 M urea, 2 M thiourea, 2% v/v CHAPS, 2% IPG buffer, pH 4–7) (GE Healthcare, Piscataway, NJ, USA). The samples were then electrofocused in a manifold cup-loading system with IPGphor (GE Healthcare, Piscataway, NJ, USA) in the following sequential steps; pre-separation at 100 V for 1 h, 200 V for 1 h, 500 V for 1 h; application of the samples to the strip gels initially at 1,000 V for 4 h; gradient focusing from 1,000 V to 8,000 V for 30 min; and finally steady-state focusing at 8,000 V for 8 h. The separated strip gels were equilibrated with equilibration buffer (6 M urea, 2% SDS, 50 mM Tris-Cl, pH 8.8, 30% glycerol) containing 65 mM DTT for 15 min. After a second equilibration with the same buffer containing 2.5% v/v iodoacetamide instead of DTT and a trace of bromphenol blue for 15 min; the equilibrated strip gels were applied to 1.0 mm thick 10% acrylamide gels and sealed with 0.25% (w/v) agarose. SDS-PAGE was carried out at 15 mA overnight using a PROTEAN IIxl 2-D Cell apparatus (BIO-RAD, Hercules, CA, USA).

### Detection of protein spots and image analysis

All sets of gels were silver-stained simultaneously in the same tray. The stained gels were then scanned using an Image Scanner III (GE Healthcare, Piscataway, NJ, USA). Spot detection, matching, normalization and quantification were automatically carried out using the ProgenesisSameSpots v5.0 (Nonlinear Dynamics, Newcastle, UK). For high reliability, same parameters, based on stringent criteria (fold difference in protein abundance> 1.5, *p*< 0.05) were applied to each set of analytical gels. The protein spots showing at least 1.5 fold difference in abundance in three replicates were subjected to MS/MS analysis for protein identification.

### Identification of proteins and their post-translational modifications by UPLC-ESI-q-TOF tandem MS

In order to identify the proteins and post-translational modifications (PTMs), peptide sequencings were performed by nanoAcquity UPLC/ESI/MS (SYNAPT HDMS, Waters Co. UK). The gel spots on 2D-PAGE were destained and digested with trypsin and the resulting peptides extracted as previously described [13]. The peptide extracts were evaporated to dryness in SpeedVac and dissolved in 10% acetonitril solution containing 1.0% formic acid. The dissolved samples were desalted on line prior to separation using trap column (5 μm particle size, NanoEase dC_18_, Waters Co., Milford, MA, USA) cartridge. Peptides were separated by using a C18 reversed-phase 75 μm i.d. × 200 mm analytical column (1.7 μm particle size, BEH130 C_18_, Waters) with an integrated electrospray ionization PicoTip (±10 μm, New Objective, USA). Peptide mixtures (5 μL) were dissolved in buffer A (Water/formic acid; 100:0.1, v/v), injected on a column and eluted by a linear gradient of 5–60% buffer B (ACN/formic acid; 100:0.1, v/v) over 120 min. Initially, the flow rate was set to 250 nL/min and the capillary voltage (2.8 keV) was applied to the UPLC mobile phase before spray. Chromatography was performed on line to SYNAPT HDMS. The mass spectrometer was programmed to record scan cycles composed of one MS scan followed by MSMS scans of the 3∼4 most abundant ions in each MS scan. MS parameters for efficient data-dependent acquisition were intensity (>10), number of components (3∼4) to be switched from MS to MS/MS analysis.

Raw data obtained from the mass spectrometer were converted to. pkl files using ProteinLynx Global Server (PLGS) 2.3 data processing software (Waters Co., Milford, MA, USA). MS/MS spectra were matched against amino acid sequences in NCBI (USA) and SwissProt using the database search program Mascot (global search engine), ProteinLynx Global SERVER (PLGS) 2.3 (Waters Co., UK).

In order to raise the MS coverage for PTM analysis, SEMSA methodology was employed [13]. The first run analysis, the 4 most abundant precursors were selected for MS/MS analysis. Following positive identification, all identified peptides from database search (Mascot) were non-redundantly excluded in the next run analysis until almost full sequence coverage was obtained. Large numbers and types of potential PTMs were considered. All reported assignments were verified by automatic and manual interpretation of spectra using the database search program Mascot (global search engine), ProteinLynx Global SERVER (PLGS) 2.3 (Waters Co., UK) and MOD^i^ (Korea, http://prix.hanyang.ac.kr/modi/) [14] in a blind mode. In addition, a minimum total score of 50, comprising at least a peptide match of ion score more than 20, was arbitrarily set as threshold for acceptance. All reported assignments were verified by automatic and manual interpretation of spectra. Each modification was assigned with an observed mass shift.

The functions of identified proteins determined using PANTHER classification system (www.pantherdb.org) are listed. The list includes proteins with a wide spectrum of cellular functions, not all which are known.

### Statistical analysis

Statistical analysis was executed using SigmaStat v2.03 (SPSS Inc., Chicago, IL, USA). Statistical comparisons were performed using two-tailed Student’s t-test between two groups and using One-way ANOVA followed by Bonferroni’s post hoc analysis for multiple comparisons among over three groups. *P* values under 0.05 were considered statistically significant. All data are presented as mean ± SE.

## Results and Discussion

### Effects of exenatide on thapsigargin-induced INS-1 cell death

**Fig 1:**
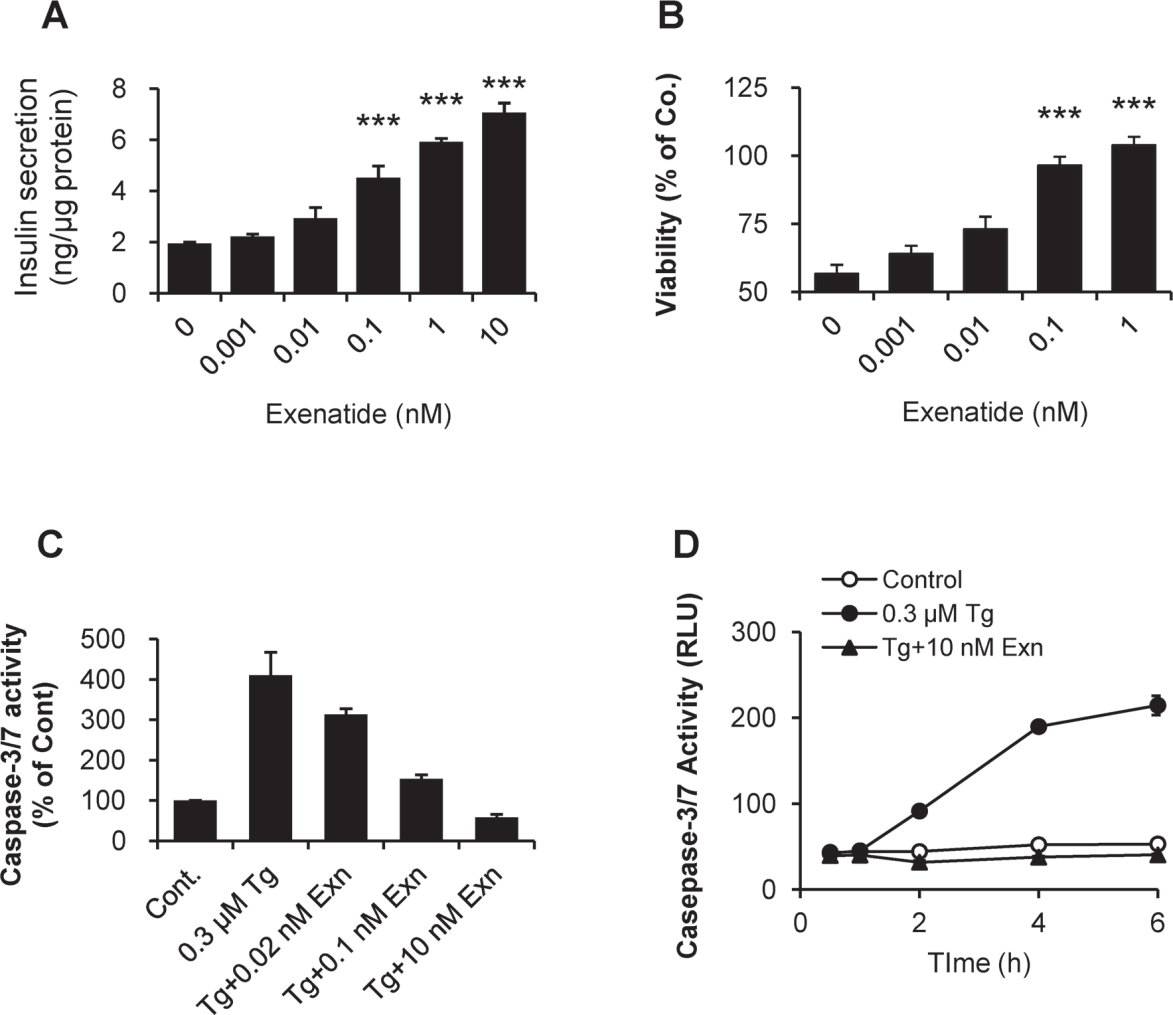
Effects of exenatide on glucose-stimulated insulin secretion and INS-1 cell death caused by thapsigargin-induced ER-stress. (A) INS-1 cells were stimulated by exenatide at 11.1 mM glucose for insulin secretion. (B) Serum-deprived INS-1 cells were treated 0.3 μM thapsigargin in the absence or presence of various concentrations of exenatide for 6 h. At the end of experiment, cellular viability was assessed by determining intracellular ATP levels. (C) After 6 h treatment, cellular caspase-3/7 activity was determined as a sensitive apoptotic marker. (D) Caspase-3/7 activity was determined according to the treatment time of 0.3 μM thapsigargin in the absence or presence of 10 nM exenatide. ***p< 0.001 vs. untreated control by Bonferroni’s t-test. Each experiment was run in triplicate.

Exenatide increased insulin secretion and cell viability in thapsigargin-treated INS-1 cells in a dose dependent manner. We first established the optimal concentration of exenatide that has maximal INS-1 cell protection against thapsigargin and increased insulin secretion in INS-1 cells. We found that 1 ∼ 10 nM exenatide led to maximal efficacy in both effects (Fig. 1A, 1B). To determine whether exenatide has protective effects via inhibiting pro-apoptotic proteases caspase-3/7 activity, we examined the dose- and time-dependence to exenatide treatment on caspase activity. We found that levels higher than 10 nM of exenatide blocked caspase-3/7 activation caused by thapsigargin treatment (Fig. 1C, 1D). Since the response of insulin secretion induced by exenatide continuously increased up to 10 nM, and our previous study [10] showed that exenatide completely blocked all types of beta cell death at 10 nM, but not at 1 nM, we further used 10 nM exenatide as the optimal concentration at which exenatide can lead to the distinct effects in INS-1 cells.

**Fig 2:**
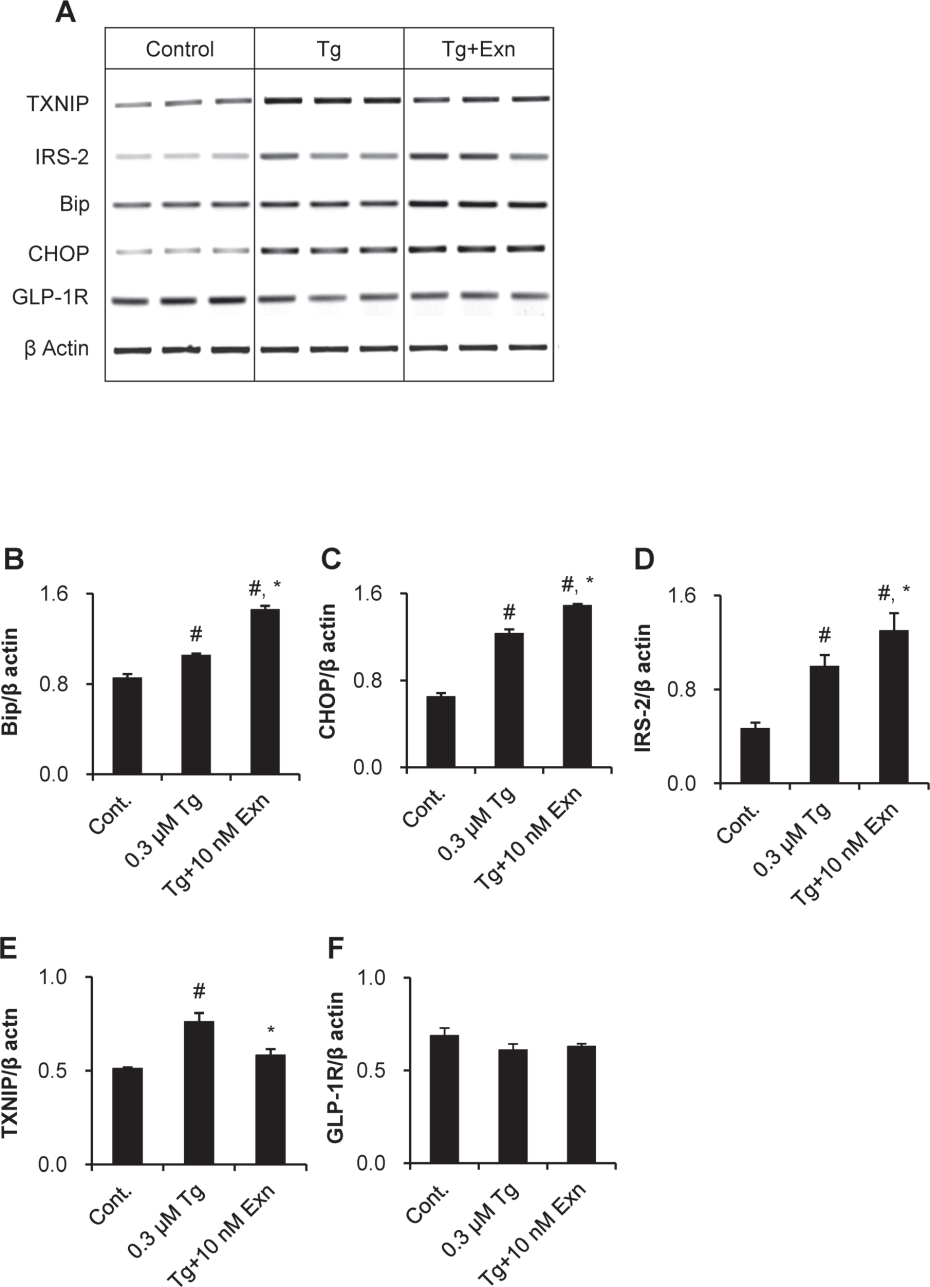
Regulation of gene expression by exenatide treatment under thapsigargin-induced INS-1 cell death. Serum-deprived INS-1 cells were treated 0.3 μM thapsigargin in the absence or presence of 10 nM exenatide for 6 h. Total RNA was isolated and mRNA expression of five genes related to the INS-1 cell death/survival was determined by RT-PCR. (A) Gel images of PCR products were obtained and quantified using densitometry. Expression levels of (B) Bip and (C) CHOP as ER stress-related chaperons, (D) IRS-2 as a signal mediator of INS-1 cell survival, (E) TXNIP as a glucotoxicity mediator, and (F) GLP-1 receptor genes as a target molecule of exenatide were assessed. Data were presented as mean ± SE from three individual determinants. #*p*< 0.05 vs. untreated control; *p< 0.05 vs. thapsigargin alone by Bonferroni’s t-test.

We then examined whether exenatide affects the expression of the ER stress- or beta cell survival-related genes, during thapsigargin-induced beta cell death. Thapsigargin treatment by itself substantially increased the expression of Bip and CHOP genes, indicating that chaperone proteins were induced to overcome ER stress. This effect became larger when the treatment was with a combination of thapsigargin and exenatide (Fig. 2A-C). This finding is in agreement with an earlier *in vitro* study [15]. Also, expression of insulin receptor substrate (IRS)-2, a signaling mediator of beta cell survival, which increased following thapsigargin treatment was also further enhanced by exenatide (Fig. 2D).

It was recently demonstrated that thioredoxin interacting protein (TXNIP), a glucotoxicity mediator and an endogenous inhibitor of thioredoxin, enhances ER stress-induced beta cell death through initiation of the inflammasome [16,17] and that exenatide protects beta cells from glucose-induced cell death, by reducing TXNIP protein levels [18]. The present study also demonstrated that thapsigargin-induced ER stress upregulated TXNIP mRNA and that exenatide blocked TXNIP up-regulation (Fig. 2E). Our results confirm that exenatide induces protein folding and replication, and decreases the inflammasome. But we found that exenatide does not affect the GLP-1R gene expression, although GLP-1R is a target molecule of exenatide (Fig. 2F). Our proteomic study thus confirms that thapsigargin induces cell death and that exenatide prevents the effects of thapsigargin and promotes cell survival.

### Protein profiles of INS-1 cells treated with thapsigargin alone and with thapsigargin plus exenatide

**Fig 3:**
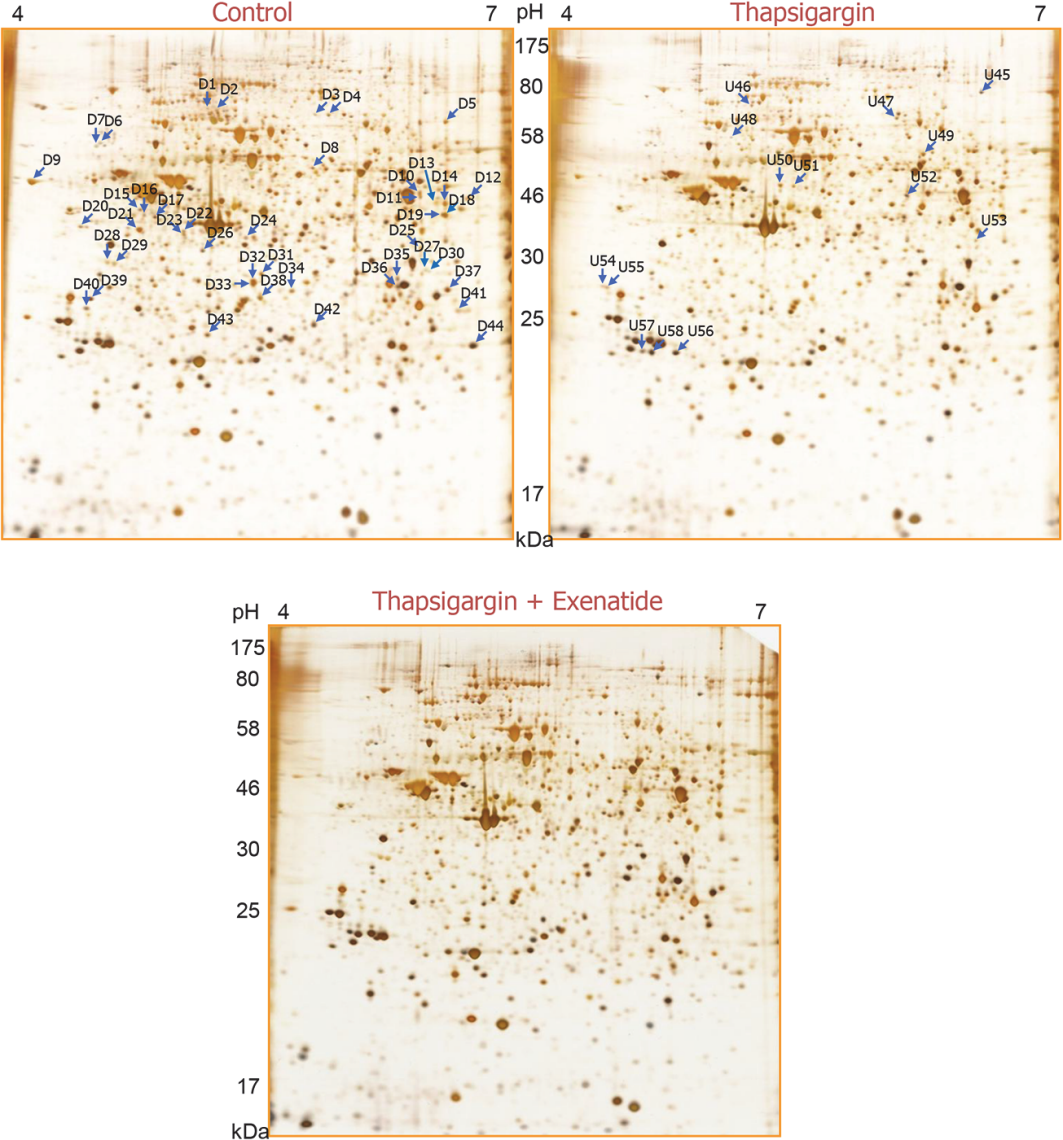
Differential protein expression during INS-1 cell death induced by ER stress and its prevention by exenatide on 2D-PAGE separation. After 6 h treatment of thapsigargin alone or thapsigargin plus exenatide, lysates of INS-1 cells were separated on 2D-PAGE and visualized by silver staining. Arrow denotes the differentially expressed protein spots shown in thapsigargin alone compared to untreated control. Direction of change was denoted as alphabet “D” (down) or “U” (up) in front of each spot number.

**Fig 4:**
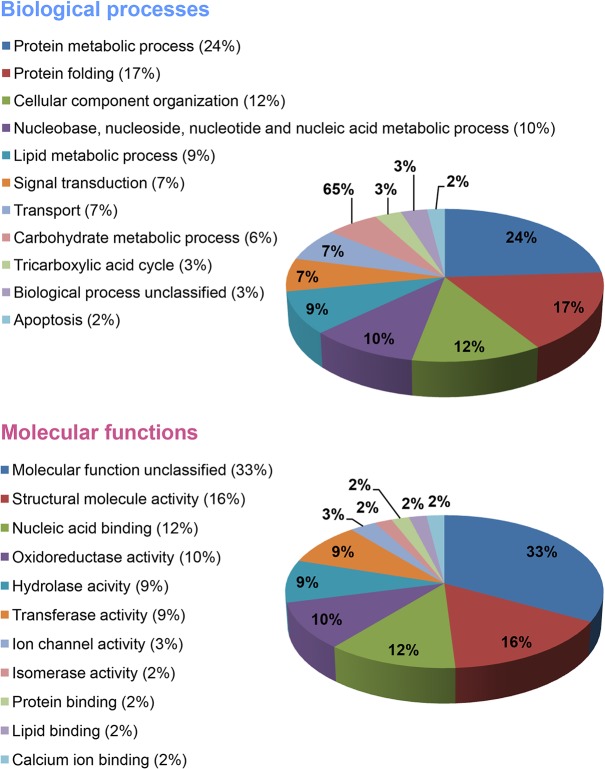
Classification of proteins altered by thapsigargin treatment based on their biological process or molecular functions using PANTHER classification system (PANTHER; www.pantherdb.org).

We conducted comparative proteomic studies to explore the overall protein profile changes in INS-1 cells treated with thapsigargin alone and with thapsigargin plus exenatide, with the goal understanding the molecular mechanisms underlying thapsigargin-induced ER stress and exenatide-induced protection or prevention of such ER stress. 2D-PAGE analysis of INS-1 cells treated with thapsigargin or thapsigargin plus exenatide revealed over 60 new protein spots that appeared in cells under thapsigargin-induced ER stress, but absent in control cells. We focused our comprehensive analysis on 58 protein spots that reproducibly appeared following these treatments (Fig. 3). Spot densities determined in triplicate runs are summarized in Table 1 and S2 Table. Of the 58 spots, 44 were statistically significantly down-regulated and 14 spots were up-regulated by thapsigargin treatment. These fifty eight spots came from 49 individual proteins, indicating multiple spots of the same protein possibly due to different modifications. As shown in Fig. 4, 24% of the 58 differentially altered proteins were associated with protein metabolic processes. These include thimet oligopeptidase and eukaryotic translation initiation factor 3 subunit 1, and 17% were concerned with protein folding. Most of the proteins associated with metabolic processes, tended to decrease and those involved in protein folding showed mixed responses. All protein spots related to carbohydrate and lipid metabolic processes were down regulated. Structural proteins (12%), nucleic acid binding proteins (10%), and proteins having oxidoreductase activity in carbohydrate or lipid metabolic processes (9%) mostly changed in response to thapsigargin treatment.

**Table 1:** Proteins differentially altered by thapsigargin treatment (N = 3).

Spot no.	Mascot score	Accession no.	Queries matched	Protein name	Mass	pI	Fold difference vs. Control			
							Mean		SE	*P value*
***Nucleobase*, *nucleoside*, *nucleotide and nucleic acid metabolic process***										
**D5**	201	Q4V7C6	14	**GMP synthase**	76709	6.21	0.464	±	0.029	1.6E-05
**D27**	103	Q3SWU3	2	**Heterogeneous nuclear ribonucleoprotein D-like**	35272	9.14	0.710	±	0.007	6.2E-09
**D30**	165	Q3SWU3	5	**Heterogeneous nuclear ribonucleoprotein D-like**	35272	9.14	0.593	±	0.018	7.4E-07
**D28**	418	P13084	12	**Nucleophosmin**	32540	4.62	0.680	±	0.025	1.4E-06
**D29**	547	P13084	20	**Nucleophosmin**	32540	4.62	0.530	±	0.007	2.7E-08
**D41**	113	Q5M827	7	**Pirin**	32158	6.22	0.616	±	0.013	1.7E-07
***Protein metabolic process***										
**D3**	409	P24155	15	**Thimet oligopeptidase**	78335	5.64	0.178	±	0.003	1.8E-07
**D4**	587	P24155	26	**Thimet oligopeptidase**	78335	5.64	0.300	±	0.011	4.0E-06
**U47**	385	P24155	20	**Thimet oligopeptidase**	78335	5.64	3.722	±	0.516	6.2E-03
**D6**	381	Q9JJP9	12	**Ubiquilin-1**	62032	4.87	0.752	±	0.043	8.0E-06
**D7**	179	Q9JJP9	11	**Ubiquilin-1**	62032	4.87	0.770	±	0.017	1.4E-07
**D8**	425	Q64303	15	**Serine/threonine-protein kinase PAK 2**	57924	5.57	0.570	±	0.008	3.7E-08
**D13**	269	Q3B8Q2	11	**Eukaryotic initiation factor 4A-III**	46811	6.30	0.670	±	0.050	1.7E-05
**D14**	179	Q68FR6	10	**Elongation factor 1-gamma**	50029	6.31	0.647	±	0.031	4.2E-06
**D15**	256	P62193	15	**26S protease regulatory subunit 4**	49154	5.87	0.568	±	0.008	3.6E-08
**D31**	640	B0BNA7	26	**Eukaryotic translation initiation factor 3 subunit I**	36438	5.38	0.734	±	0.009	1.2E-08
**D36**	197	P19945	10	**60S acidic ribosomal protein P0**	34194	5.91	0.512	±	0.058	1.2E-04
**D40**	156	P38983	6	**40S ribosomal protein SA**	32803	4.80	0.529	±	0.007	2.7E-08
**D43**	324	P18422	18	**Proteasome subunit alpha type-3**	28401	5.29	0.672	±	0.020	6.1E-07
**U52**	308	Q01205	12	**Dihydrolipoyllysine-residue succinyltransferase component of 2-oxoglutarate dehydrogenase complex, mitochondrial**	48894	8.89	1.434	±	0.075	4.4E-03
***Carbohydrate metabolic process***										
**D35**	239	P11980	8	**Pyruvate kinase isozymes M1/M2**	57781	6.63	0.568	±	0.017	9.6E-07
**D37**	346	P07943	13	**Aldose reductase**	35774	6.26	0.709	±	0.022	1.3E-10
**D44**	564	P25113	35	**Phosphoglycerate mutase 1**	28814	6.67	0.740	±	0.038	5.0E-06
***Lipid metabolic process***										
**D10**	348	O35077	17	**Glycerol-3-phosphate dehydrogenase [NAD+], cytoplasmic**	37428	6.16	0.418	±	0.051	4.0E-04
**D11**	146	O35077	11	**Glycerol-3-phosphate dehydrogenase [NAD+], cytoplasmic**	37428	6.16	0.222	±	0.019	1.4E-04
**D25**	351	O35077	16	**Glycerol-3-phosphate dehydrogenase [NAD+], cytoplasmic**	37428	6.16	0.540	±	0.018	1.4E-06
**U53**	48	O35077	4	**Glycerol-3-phosphate dehydrogenase [NAD+], cytoplasmic**	37428	6.16	1.816	±	0.200	1.5E-02
**D42**	64	Q8CIN7	2	**Inositol monophosphatase 2**	31776	5.68	0.555	±	0.004	3.2E-09
***Protein folding***										
**D1**	446	Q66HA8	21	**Heat shock protein 105 kDa**	96357	5.4	0.383	±	0.033	7.1E-05
**D2**	94	Q66HA8	3	**Heat shock protein 105 kDa**	96357	5.4	0.622	±	0.042	1.7E-05
**U49**	62	Q66HA8	2	**Heat shock protein 105 kDa**	96357	5.4	2.703	±	0.038	1.5E-06
**D9**	686	P18418	43	**Calreticulin**	47966	4.33	0.649	±	0.040	1.1E-05
**D20**	410	P34058	20	**Heat shock protein HSP 90-beta**	83229	4.97	0.443	±	0.003	4.6E-09
**U46**	203	P34058	8	**Heat shock protein HSP 90-beta**	83229	4.97	2.084	±	0.312	2.5E-02
**U54**	133	P34058	6	**Heat shock protein HSP 90-beta**	83229	4.97	5.087	±	0.586	2.2E-03
**U55**	313	P34058	16	**Heat shock protein HSP 90-beta**	83229	4.97	7.797	±	0.576	3.0E-04
**D21**	299	P63018	14	**Heat shock cognate 71 kDa protein**	70827	5.37	0.719	±	0.009	1.5E-08
**D39**	178	P63018	16	**Heat shock cognate 71 kDa protein**	70827	5.37	0.564	±	0.024	3.0E-06
***Tricarboxylic acid cycle***										
**D12**	139	P16638	11	**ATP-citrate synthase**	1E+05	6.96	0.340	±	0.072	4.5E-03
**D34**	278	P42123	19	**L-lactate dehydrogenase B chain**	36589	5.7	0.755	±	0.013	5.6E-08
***Signal transduction***										
**D38**	138	P54311	5	**Guanine nucleotide-binding protein G(I)/G(S)/G(T) subunit beta-1**	37353	5.6	0.745	±	0.022	4.4E-07
**U56**	238	P35213	11	**14–3–3 protein beta/alpha**	28037	4.81	15.693	±	0.175	1.2E-07
**U57**	205	P68255	8	**14–3–3 protein theta**	27761	4.69	7.464	±	0.341	4.6E-05
**U58**	317	P62260	22	**14–3–3 protein epsilon**	29155	4.63	13.220	±	1.034	2.9E-04
***Transport***										
**D16**	69	P10719	2	**ATP synthase subunit beta, mitochondrial**	56318	5.18	0.380	±	0.009	5.4E-07
**D17**	323	P10719	11	**ATP synthase subunit beta, mitochondrial**	56318	5.18	0.788	±	0.011	3.9E-09
**D18**	70	P85515	1	**Alpha-centractin**	42587	6.19	0.777	±	0.010	2.0E-08
**D19**	239	P85515	9	**Alpha-centractin**	42587	6.19	0.740	±	0.012	4.2E-08
***Cellular organization***										
**D22**	64	P62738	3	**Actin, aortic smooth muscle**	41982	5.24	0.352	±	0.012	2.3E-06
**D23**	186	P63259	10	**Actin, cytoplasmic 2**	41766	5.31	0.432	±	0.004	7.1E-09
**D32**	414	Q6P9V9	25	**Tubulin alpha-1B chain**	50120	4.94	0.772	±	0.013	4.3E-08
**D33**	414	Q6P9V9	25	**Tubulin alpha-1B chain**	50120	4.94	0.718	±	0.057	3.4E-05
**U48**	116	Q62871	6	**Cytoplasmic dynein 1 intermediate chain 2**	71134	5.11	3.407	±	0.194	2.4E-04
**U50**	331	P68370	14	**Tubulin alpha-1A chain**	50104	4.94	3.377	±	0.262	8.2E-04
**U51**	361	P68370	21	**Tubulin alpha-1A chain**	50104	4.94	6.029	±	0.353	1.4E-04
***Apoptosis***										
**U45**	448	Q9QZA2	16	**Programmed cell death 6-interacting protein**	96570	6.15	1.467	±	0.025	4.8E-05
***Biological process unclassified***										
**D24**	50	P04764	1	**Alpha-enolase**	47098	6.16	0.296	±	0.005	1.6E-07
**D26**	206	Q6JE36	6	**Protein NDRG1**	42927	5.77	0.377	±	0.031	5.1E-05

From comparative differential 2D-PAGE analysis between untreated control and thapsigargin alone, 58 protein spots which were significantly altered by thapsigargin-induced INS-1 cell death were classified according to the biological process. Data were denoted as mean ± SE of fold differences vs. control from three individual determinants. Its statistical significance against untreated control was analyzed by Student’s t-test. Symbols in front of spot number present the up (U) or down (D) regulation of spot change, and same color spots denote same protein having several spots on 2D-PAGE.

**Fig 5:**
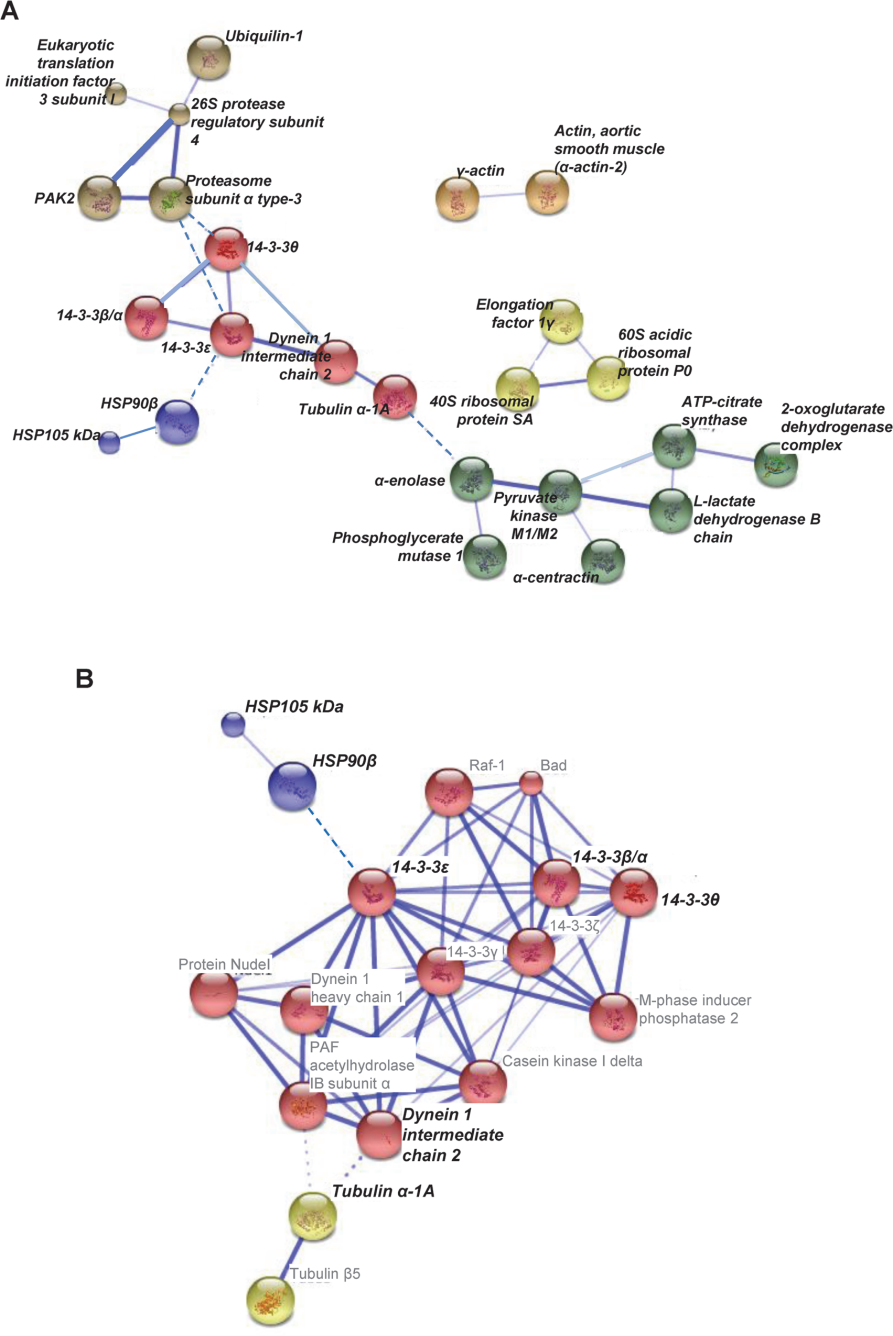
Protein networks; (A) proteins altered by thapsigargin-induced INS-1 cell death, (B) predicted protein interaction by String analysis (www.string-db.org). Proteins altered by thapsigargin in our experiment were denoted as bold and italic.

String analysis (www.string-db.org) and other published information suggest that nineteen of the proteins altered by thapsigargin treatment may be part of a linked network. This protein network includes signaling molecules such as 14–3–3 isoforms that mediate signals to Hsps (chaperons), protein metabolism-related proteins and lipid-carbohydrate metabolism-related proteins (Fig. 5A). String map suggested that the network may be expanded, to include potential binding partners; i) stress-related Bad and Raf1; ii) cytoskeleton-related M-phase inducer phosphatase, nuclear distribution protein nudE-like 1, dynein heavy chain; iii) signaling-related casein kinase Iδ, 14–3–3 (Fig. 5B). The possible pathways involved in ER stress in insulinoma cells can be inferred from the combined findings from proteomic studies and informatic string analysis.

### Comparison of protein expressions in cells treated thapsigargin and thapsigargin plus exenatide

**Fig 6:**
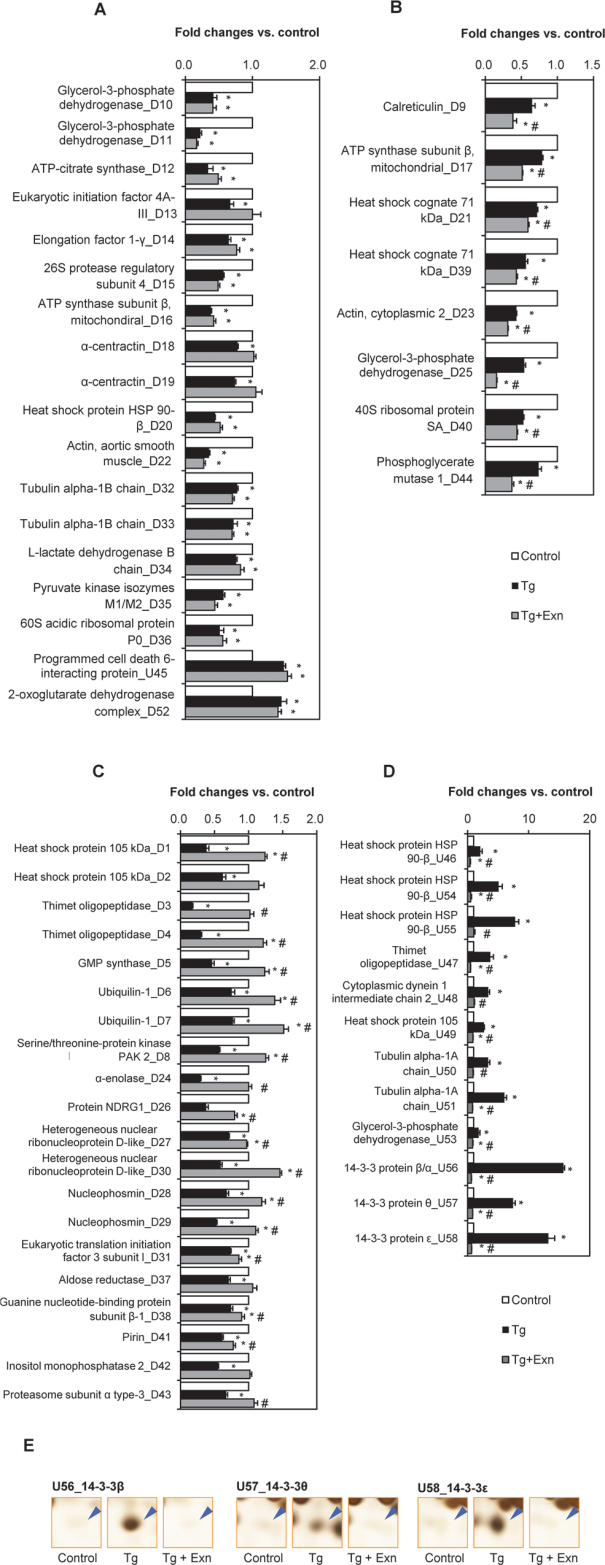
Effects of exenatide on proteins which were up- or down-regulated during thapsigargin-induced INS-1 cell death. (A) Eighteen protein spots altered by thapsigargin alone were not changed by addition of exenatide. (B) Change of eight protein spots among total 58 protein spots significantly altered by thapsigargin alone, was augmented by exenatide add-on. (C) Twenty protein spots decreased by thapsigargin-induced INS-1 cell death were significantly reversed by addition of exenatide. (D) Increase of twelve protein spots by thapsigargin alone was completely blocked by exenatide add-on. Data were presented as mean ± SE from three individual determinants. (E) Enlarged spot images of 14–3–3 isoforms. *p< 0.05 vs. control; #*p*< 0.05 vs. thapsigargin alone by Bonferroni’s t-test.

To investigate how exenatide protects ER stress induced cell death, we compared the differentially expressed proteins in cells treated thapsigargin and thapsigargin plus exenatide. Treatment with thapsigargin resulted in changes in fifty eight protein spots. Results of treatment with thapsigargin plus exentide are as follows. 18 of the fifty eight spots were unaffected (Fig. 6A, S3 Table); 8 spots were exaggerated (Fig. 6B, S4 Table); and changes in 32 protein spots were reversed (Fig. 6C-D, S5 Table). The latter included twelve proteins that were up-regulated by thapsigargin (Fig. 6D). The total list of proteins identified with MS/MS is shown in S6 Table. The appearance of 14–3–3β, θ, and ε isomers after treatment with thapsigargin and its complete blocking by exenatide (Fig. 6E) is a significant feature of the manifold protein changes involved in the protective effect of exenatide on the cell death caused by thapsigargin induced ER stress.

**Fig 7:**
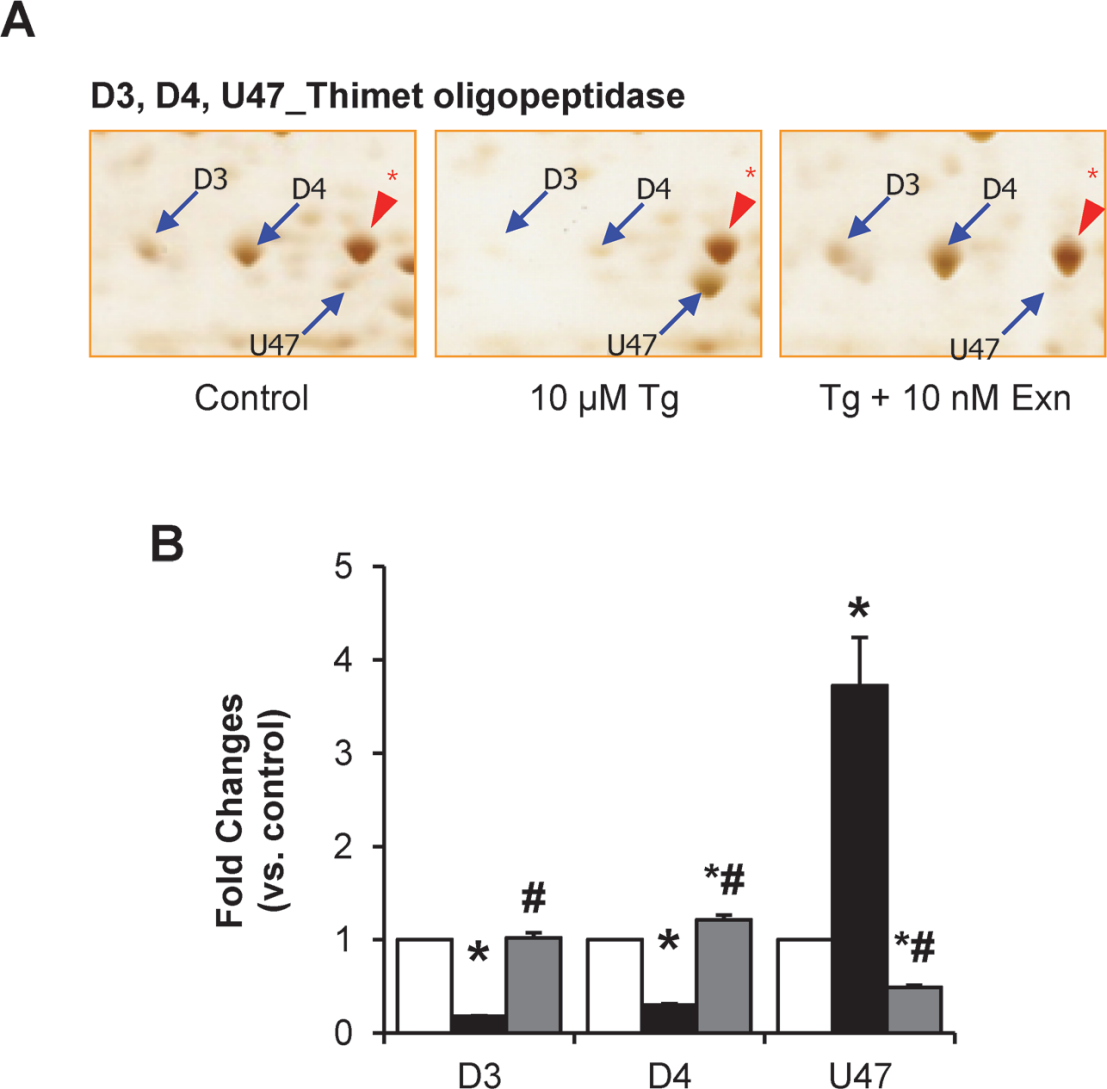
Changes in post-translational modifications of thimet oligopeptidase in response to thapsigargin and thapsigargin plus exenatide. Protein spots at a different modification status were quantified and data were presented as mean ± SE from three individual determinants. *p< 0.05 vs. control; #*p*< 0.05 vs. thapsigargin alone by Bonferroni’s t-test.

We therefore tried to dissect the functions of the proteins subjected to thapsigargin induced changes but subsequently restored by exenatide treatment. We broadly classified them into four groups: i) proteins such as HSP90, HSP105, thimet oligopeptidase, ubiquilin-1, PAK2, and proteasome subunit α type-3 concerned with protein metabolic processes; ii) proteins such as GMP synthase, pirin, nucleophosmin, guanine-binding proteins, concerned with nucleotide metabolic processes; iii) proteins such as 14–3–3 isoforms, related to signal transduction and iv) cellular structural proteins such as tubulin. We found it interesting that proteins related to translation machinery or glucose metabolism were not affected by thapsigargin plus exenatide treatment. Fourteen of the proteins discussed above contained different post-translational modifications (PTMs). Also, we noted in some cases that all spots representing the same protein were altered by the treatments. For example, various spots of HSP71, HSP105, α-centractin, nuclear ribonucleoprotein D-like, nucleophosmin, and ubiquilin-1 represented different alterations produced by exenatide treatment. In the case of HSP90, ATP synthase subunit β, glycerol-3-phosphate dehydrogenase, thimet oligopeptidase and 14–3–3 isoforms, exenatide altered only some spots significantly changed by thapsigargin (S1 Fig.; Fig. 7). Exenatide blocked thapsigargin caused alterations in only three (D3, D4, and U47) of the four spots representing thimet oligopeptidase involved in intracellular protein metabolism (Fig. 7A, 7B). We further examined the PTMs of thimet oligopeptidase employing proteomics and identified 4 different modifications including oxidative modifications with the conversion of cysteine (Cys) to dehydroalanin (Dha) or serine (Ser) at Cys^175^ residues as described previously [19] (Table 2 and S2 Fig.). Several phosphorylations at Ser^172^, Thr^656^, Ser^89^ of spot D4 and at The^656^ of U47 were identified. This indicates that phosphorylations of Ser^172^ and Ser^89^ that were not detectable in spot U47, appeared only in response to thapsigargin as more basic spot. This suggests that thimet oligopeptidase is regulated by phosphorylation and its phosphorylated form is dephosphorylated in ER stress induced by thapsigargin. This suggested regulatory mechanism for thimet oligopeptidase during ER stress and its protection by exenatide, should be verified experimentally. If confirmed, phosphorylation changes in thimet oligopeptidase may serve as biomarkers of ER stress.

**Table 2:** Modifications of thimet oligopeptidase identified on 2D-PAGE (Fig. 7) using peptide sequencing with MS/MS analysis.

Spot no.	a.a	m/z	Mr (cal.)	Mascot score	Observed modification
*	not determined				
D3	not determined				
D4	**79–93**	**729.8090**	**1457.7102**	**33**	**RL** ***S*** **LL** ***C*** **IDFNK + Phospho (ST); Carbamidomethyl (C)**
	169–181	566.2813	1130.6213	56	LSLL***C***IDFNK + Dehydroala (C)
	170–181	575.2861	1148.6213	60	LSLL***C***IDFNK + Cys to Ser (C)
	**170–181**	**794.8166**	**1587.7446**	**24**	**NILDFPQHV** ***S*** **PNK + Phospho (ST)**
	642–660	929.8585	1857.8543	33	TSILRPGGSEDAS***TM***LK + Phospho (ST); Oxidation (M)
U47	170–181	566.2836	1130.6213	68	LSLL***C***IDFNK + Dehydroala (C)
	170–181	575.2897	1148.6213	45	LSLL***C***IDFNK + Cys to Ser (C)
	642–660	929.8628	1587.7446	23	TSILRPGGSEDAS***TM***LK + Phospho (ST); Oxidation (M)

Differentially modified peptides between spot D4 and U47 were indicated as bold characters.

### Appearance of 14–3–3 protein isoforms is prominently associated with thapsigargin-induced INS-1 cell death

**Fig 8:**
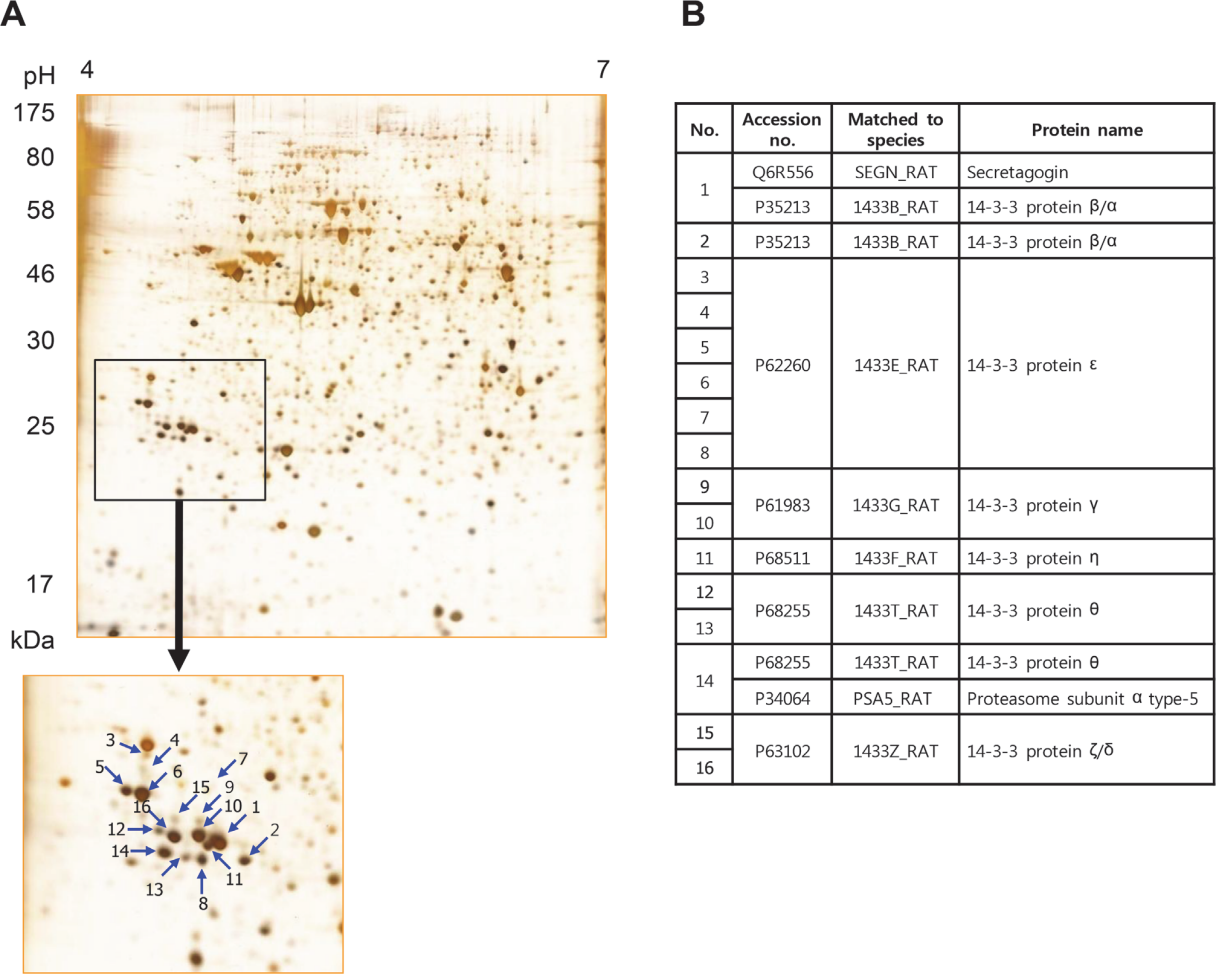
Identification of 14–3–3 family proteins by 2-D PAGE. From lysates of thapsigargin-treated INS-1 cells, total 16 protein spots of 14–3–3 proteins were separated on 2D-PAGE and PTMs in each spot were determined by peptide sequencing with MS/MS analysis.

In our previous studies, we demonstrated that reduction in 14–3–3θ causes for ER stress in INS-1 cells [10]. Moreover our data suggested that the appearance of specific 14–3–3 isoform spots correlates with the appearance of the apoptotic signal and cell death under ER stress. Previous studies reported changes in 14–3–3 proteins in INS-1 cells exposed to alternative ER stress inducers, but these changes included only alterations in 14–3–3β and ζ levels, but not their PTMs status [20]. In this study we attempted to identify the specific isoforms as well as the modifications of 14–3–3 proteins that might be involved in ER stress-caused INS-1 cell death. As shown in Fig. 8, 16 spots representing isoforms of 14–3–3 protein were identified; six of 14–3–3ε; three of 14–3–3θ; two of 14–3–3β, ζ and γ each and one of 14–3–3η. Of the seven isoforms of 14–3–3 proteins, 14–3–3σ was not detected on 2D-PAGE, because it is absent in INS-1 cells, but exists only in pancreatic ductal adenocarcinoma cells [21]. Significantly, β, θ and ε isoforms of 14–3–3 protein respectively appeared in the thapsigargin changed spots: U56, U57 and U58 (Fig. 6E, Table 1).

### Exenatide completely blocks modifications of 14–3–3 proteins occurring under thapsigargin-induced ER stress

**Fig 9:**
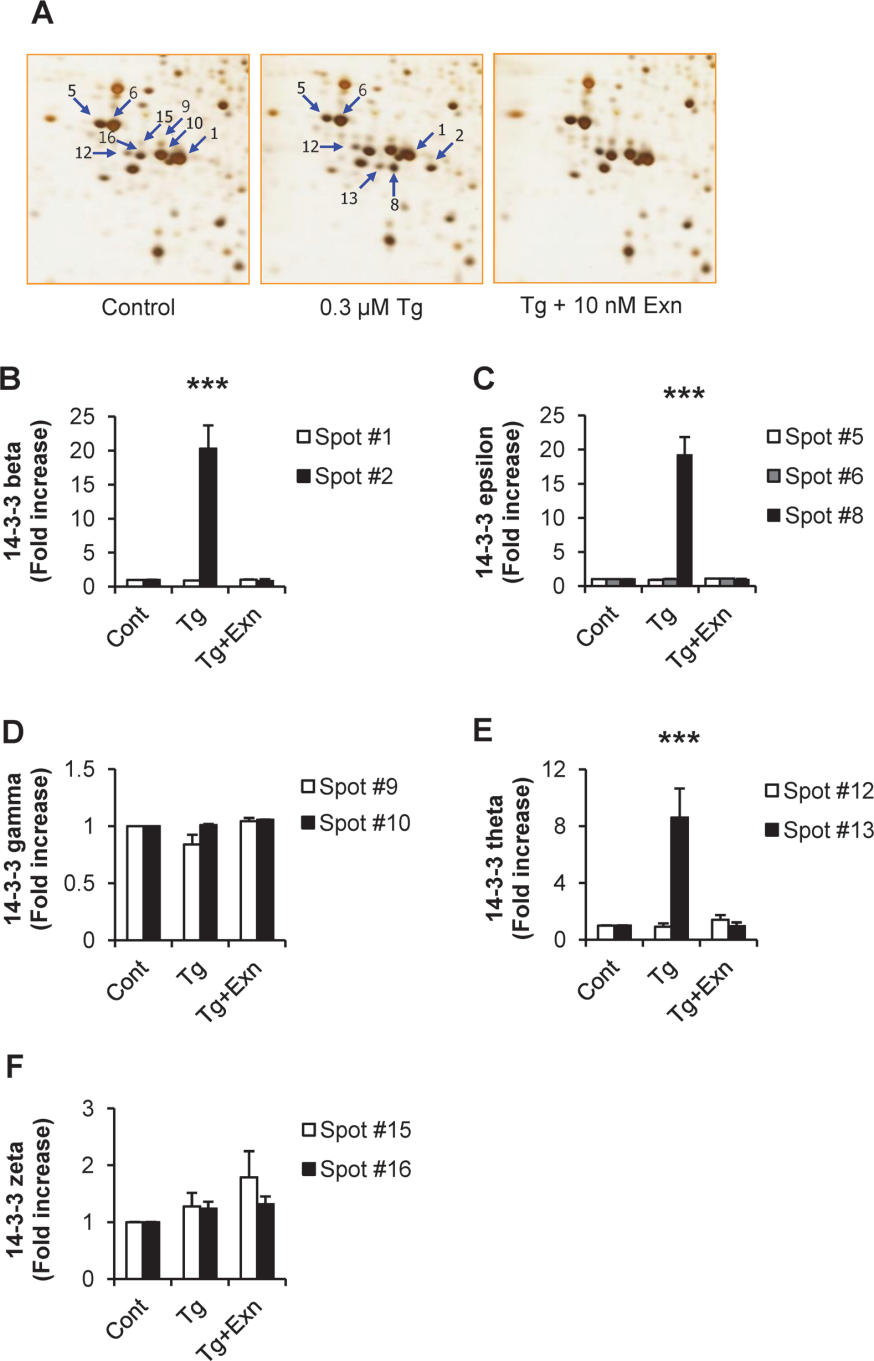
Changes in post-translational modifications of 14–3–3 proteins during thapsigargin and thapsigargin plus exenatide treatments. Protein spots of each 14–3–3 isoform at a different modification status were quantified and data were presented ad mean ± SE from three separate experiments. ***p< 0.001 vs. control by Bonferroni’s t-test.

The spots of 14–3–3 isoforms appearing when INS-1 cells were treated with thapsigargin alone or thapsigargin plus exenatide, were analyzed. As shown in Fig. 9, 14-3-3ε spot no. 8 significantly increased during thapsigargin-induced cell death without discernible changes in unmodified spot 5 or modified spot 6. 14–3–3β also showed no changes in its unmodified abundant form in spot no. 1, while spot no. 2 considerably increased with INS-1 cell death. 14–3–3θ also significantly increased as spot no. 13 compared to spot no. 12. 14–3–3ζ tended to increase on treatment with thapsigargin alone or thapsigargin plus exenatide. Both the spots of 14–3–3ζ changed similarly. 14–3–3γ showed no significant change following either treatment. Spots which could not be separated from other spots (e.g. spot no. 14), or remained unchanged (e.g. spot no. 11 of 14–3–3η), or were too faint (e.g. spots no. 3, 4 and 7) were not included in this study. These results suggest that 14–3–3 isoforms were altered both during thapsigargin induced cell death and during its reversal by exenatide.

### Identification of ER stress related post-translational modifications of 14–3–3 proteins

Among the 14–3–3 protein isoform spots, spots 2, 13, and 8, respectively identical to U56, U57, and U58 shown in Table 1, differently changed in response to thapsigargin and restored by exenatide treatment (Fig. 9) suggesting that they play critical roles in ER stress. We examined the PTMs of these 14–3–3 isoforms employing MS methodology with SEMSA and the searching algorithm MOD^i^. As shown in Table 3, a single isoform of 14–3–3 appears in several spots, showing different PTMs including acetylation at Lys residue, oxidation at Met and Cys, and phosphorylation at Ser, Thr and Tyr. Also, a single spot of 14–3–3 showed several modifications. As examples, 14–3–3ε showed six spots with a broad range of molecular weights, probably products of cleavage by caspase [22]. Spot 6, 14–3–3ε, a more abundant and more acidic spot than spot 8, showed phosphorylation at Tyr^20^, Ser^156^ and Thr^205^, while spot 8, which appeared after thapsigargin treatment and restored after exenatide treatment, contained acetylation at Lys^29^ and phosphorytions at Thr^28^, Ser^156^ and Thr^205^. Spot 8 appeared with a lower molecular weight and more basic position because of acetylation at Lys^29^, dephosphorylation at Tyr^20^ and phosphorylation at Thr^28^. This indicates that PTMs of 14–3–3 are changed during ER stress with thapsigargin and recovered with exenatide. Spot 2 is presumed to result from phosphorylations at Thr^32^ and Tyr^106^ of 14–3–3β and phosphorylation at Ser^92^ of spot no. 13 (Table 3, underlined; S2–4 Fig.). These results suggest that specific PTMs of 14–3–3 β, ε, and θ including phosphorylations, contribute to signaling in thapsigargin-induced ER stress. Protein spots no. 8 and 2 of 14–3–3ε and β, which were produced by thapsigargin treatment, reflect the heterogeneity in each modification, which makes it difficult to decide which particular modification contributed to the position shift. On the other hand, protein 14–3–3ζ (spot no. 16) phosphorylated at Ser^190^ and 14–3–3γ (spot no. 10) phosphorylated at Thr^31^ were homogeneous entities. We ruled out 14–3–3ζ and γ for further study because they changed minimally during ER stress. Intriguingly, 14–3–3θ showed identical modification in each spot, indicating that phosphorylation at its Ser^92^ contributed to the shift of 14–3–3θ because spot no. 12 and spot no. 13 were both acetylated at Lys^3^ (Table 3, Fig. 9, S5–7 Figs.). These modifications also appeared in palmitate-induced (unpublished data) and in streptozotocin-induced INS-1 cell death [10]. It appears that phosphorylations of 14–3–3β, ε and θ may contribute to the signal pathway in INS-1 cell death, but how this pathway is regulated in ER stress and recovery is not clear.

**Table 3:** Modifications of 14–3–3 isoforms identified on 2D-PAGE (Fig. 8) using peptide sequencing with MS/MS analysis.

Protein	Spot no.	a.a	m/z	Mr (cal.)	Mascot score	Observed modification
**14–3–3β/α**	spot 1					
	**spot 2**	**30–43**	**839.8474**	**1,677.70**	**34**	**AV** ***T*** **EQGHELSNEER + Phospho (ST)**
		**106–117**	**720.3335**	**1,438.69**	**21**	***Y*** **LILNATHAESK + Phospho (ST)**
**14–3–3ε**	spot 3					
	spot 4					
	spot 5					
	spot 6	**13–28**	**696.9818**	**2,087.80**	**20**	**LAEQAER** ***Y*** **DEMVE** ***S*** **MK + Phospho (ST); Phospho (Y)**
		**197–215**	**1084.468**	**2,166.92**	**34**	**AAFDDAIAELD** ***T*** **LSEESYK + Phospho (ST)**
		154–170	958.4717	1,914.89	61	AA***S***DIA***M***TELPPTHPIR + Oxidation (M); Phospho (ST)
	spot 7					
	**spot 8**	154–170	958.4717	1,914.89	39	AA***S***DIA***M***TELPPTHPIR + Oxidation (M); Phospho (ST)
		**29–42**	**429.1172**	**1,712.77**	**24**	***K*** **VAG** ***M*** **DVEL** ***T*** **VEER + Acetyl (K); Oxidation (M); Phospho (ST)**
**14–3–3γ**	spot 9	153–162	607.7217	1,213.50	20	AY***S***EAHEISK + Phospho (ST)
	spot 10	29–42	862.4017	1,722.75	50	NV***T***ELNEPLSNEER + Phospho (ST)
		153–62	607.7471	1,213.50	47	AY***S***EAHEISK + Phospho (ST)
**14–3–3η**	spot 11	92–106	877.9100	1,753.82	36	ELE***T***VCNDVLALLDK + Phospho (ST)
		29–52	833.9036	1,665.72	34	AV***T***ELNEPLSNEDR + Phospho (ST)
		92–106	906.4291	1,810.84	39	ELE***T***V*C*NDVLALLDK + Carbamidomethyl (C); Phospho (ST)
**14–3–3θ**	spot 12	1–9	589.2384	1,176.61	20	***M***E***K***TELIQK + Acetyl (K); Oxidation (M)
	**spot 13**	1–9	589.2384	1,176.61	26	***M***E***K***TELIQK + Acetyl (K); Oxidation (M)
		**92–103**	**707.8676**	**1,413.68**	**40**	***S*** **ICTTVLELLDK + Phospho (ST)**
**14–3–3ζ/** δ	spot 15	1–9	581.7730	1,161.57	29	***M***D***K***NELVQK + Acetyl (K); Oxidation (M)
	spot 16	1–9	581.7884	1,161.57	23	***M***D***K***NELVQK + Acetyl (K); Oxidation (M)
		92–103	721.3329	1,440.66	21	DICNDVL***S***LLEK + Phospho (ST)
		92–103	749.8460	1,497.68	27	DI*C*NDVL***S***LLEK + Carbamidomethyl (C); Phospho (ST)
		140–158	1124.9850	2,247.96	62	GIVDQ***S***QQAYQEAFEISKK + Phospho (ST)
		188–212	711.3508	2,841.27	46	A*C* ***S***LAKTAFDEAIAELDTLSEESYK + Carbamidomethyl (C); Phospho (ST)

Differentially modified peptides in each isoform were indicated as bold characters.

### Prediction of potential kinases involved in phosphorylation14–3–3

**Fig 10:**
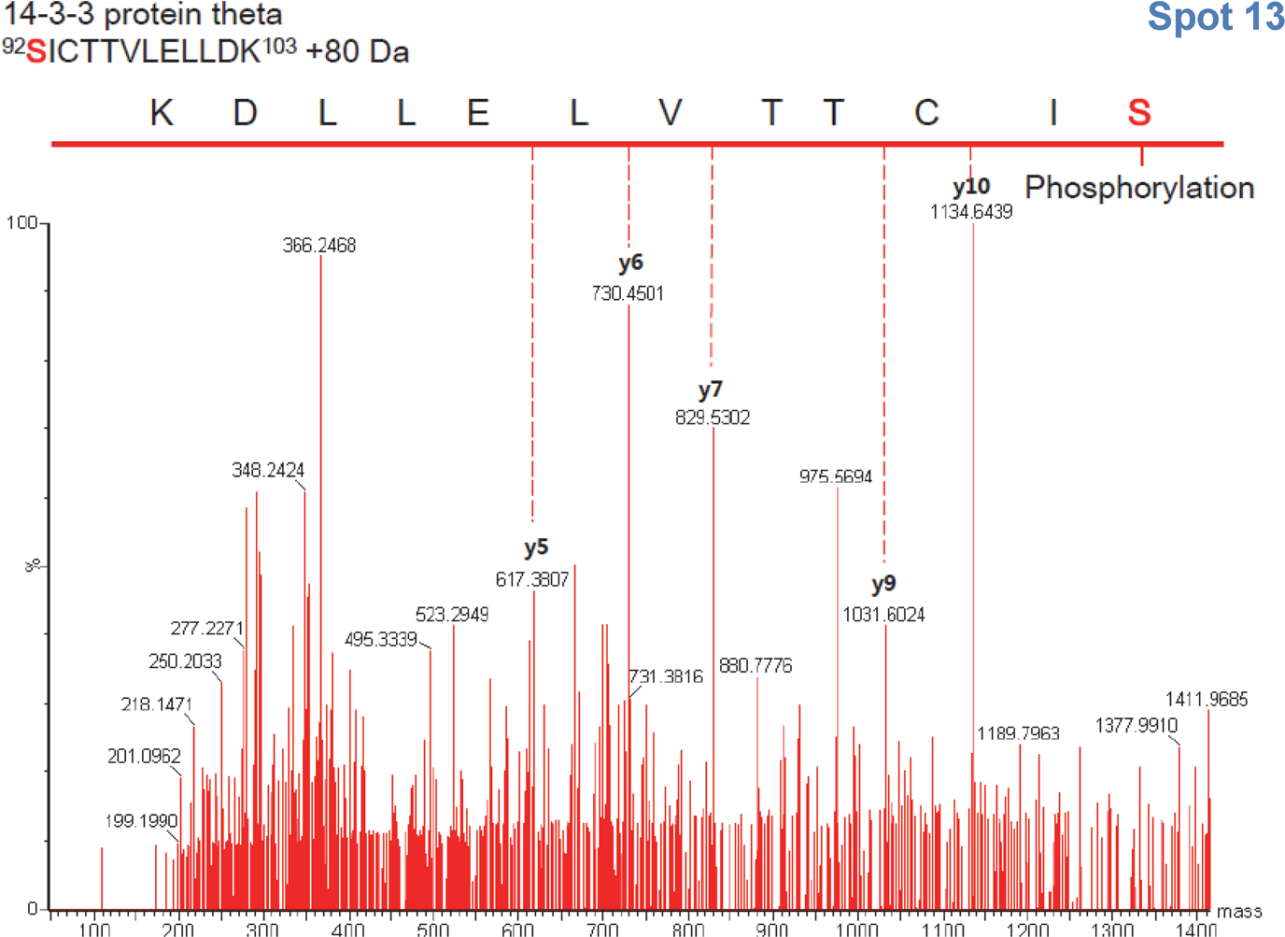
MS/MS Spectrum of 14–3–3θ peptide phosphorylated at Ser^92^ (^92^SICTTVLELLDK^103^ of spot No. 13).

In order to determine which kinases are phosphorylating 14–3–3 in response to ER stress, we employed *in silico* prediction using various available software listed below. 14–3–3β, ε, and ζ have a common JNK phosphorylation site (Ser-Pro) [9], while 14–3–3θ has no consensus sequences (Ser-Pro or Thr-Pro) for JNK phosphorylation. We tried to predict kinases potentially phosphorylating rat 14–3–3θ at Ser^92^ which was identified by MS/MS (Fig. 10).

For identification of cognate protein kinases, three softwares were applied; scansite available at http://scansite.mit.edu/motifscanseq.phtml, NetPhosK 1.0 at http://www.cbs.dtu.dk/services/NetPhosK/, and GPS 2.1 at http://gps.biocuckoo.org/ [23]. NetPhosK predicted ribosomal s6 kinase (RSK), protein kinase G (PKG), and cell division control protein 2 (cdc2; also known as cyclin-dependent kinase (CDK)-1) for Ser^63^, casein kinase (CK) II for Ser^88^ and Ser^156^, and CKI/II for Ser^232^, but it did not predict phosphorylation at Ser^92^ of 14–3–3θ. Scansite also did not predict phosphorylation for Ser^92^ (data not shown). On the other hand, GPS 2.1 predicted cognate kinases for five Ser phosphorylations except for one at Ser^63^ among five sites which were predicted by NetPhos. Cognate kinases with highest score were Akt2 for Ser^88^, NimA related kinase (NEK)-2 for Ser^156^, mitogen-activated protein kinase kinase kinase 8 (MAP3K8; also called as COT) for Ser^210^, and Ca^2+^/calmodulin-dependent protein kinases (CaMK)1a for Ser^232^. For Ser^92^ of 14–3–3θ, predicted cognate kinases are listed with score in Table 4. CaMK1a was ranked with the highest priority. Protein kinase B or protein kinase C was also proposed, but with relatively lower scores. Other kinases were predicted as potential kinases phosphorylating spots no. 2 and 8 of 14–3–3α/β and 14–3–3ε. Mitogen-activated protein kinase kinase (MAPKK) known as a survival signal, was predicted with a high score as the phosphorylating kinase for both spots no. 2 and 8; MAP2K1 for phosphorylation at Thr^32^ of 14–3–3β/α, MAP2K1 or MAP2K7 for phosphorylation at Thr^38^ and MAP2K2 at Ser^156^ of 14–3–3ε. These results suggest that i) CaMK1a might be the kinase involved in phosphorylation of 14–3–3θ under ER stress-induced INS-1 cell death, and ii) MAPKK might play a role in phosphorylation of 14–3–3β/α and 14–3–3ε for survival signaling.

**Table 4:** Prediction of kinases possibly promoting the phosphorylation of 14–3–3 using GPS v2.1 (http://gps.biocuckoo.org/).

*Isoform*	*Spot #*	*Peptide*	*Position*	*Code*	*Kinase*	*Score*	*Cutoff*
**β/α**	**2**	AAAMKAV***T***EQGHELS	32	T	CK1	1.156	1.091
			32	T	AGC/GRK	1.131	0.583
			32	T	CK1/CK1	1.575	1.521
			32	T	STE/STE-Unique	2.286	1.571
			32	T	Other/PEK	2.188	2.000
			32	T	AGC/GRK/GRK	1.958	1.583
			32	T	CAMK/DAPK/DAPK3	2.944	2.889
			32	T	CK1/CK1/CK1a	1.882	1.118
			**32**	**T**	**STE/STE7/MAP2K1**	**11.000**	**5.000**
			32	T	STE/STE7/MAP2K2	4.000	4.000
			32	T	Other/NEK/NEK2	5.667	4.333
			32	T	Other/PEK/PKR	2.062	2.062
			32	T	Other/PLK/PLK1	1.149	1.021
			32	T	AGC/PKC/Alpha/PKCg	2.000	1.600
**ε**	**8**	AGMDVEL***T***VEERNLL	38	T	AGC/DMPK	1.385	1.256
			38	T	AGC/PKC/Iota	3.077	2.769
			**38**	**T**	**STE/STE7/MAP2K1**	**7.000**	**5.000**
			**38**	**T**	**STE/STE7/MAP2K7**	**5.400**	**1.800**
			38	T	TKL/MLK/ILK	3.000	2.444
			38	T	Other/PLK/PLK1	1.277	1.021
			38	T	AGC/GRK/GRK/GRK-5	2.091	1.545
			38	T	AGC/PKC/Iota/PKCz	2.769	2.692
		LVAYKAA***S***DIAMTEL	156	S	AGC/PKB	1.588	1.118
			156	S	CK1/CK1/CK1e	0.857	0.643
			**156**	**S**	**STE/STE7/MAP2K2**	**5.000**	**4.000**
			156	S	AGC/GRK/GRK/GRK-1	3.500	2.875
**θ**	**13**	KVESELR***S***ICTTVLE	92	S	AGC/PKB	2.91	1.12
			92	S	CAMK/CAMK1	2.00	1.88
			92	S	CAMK/CAMKL	2.58	1.87
			92	S	STE/STE-Unique	1.57	1.57
			92	S	Other/IKK	1.06	0.53
			92	S	AGC/PKB/PDK1	2.09	1.06
			**92**	**S**	**CAMK/CAMK1/CAMK1a**	**8.00**	**3.75**
			92	S	CAMK/CAMKL/LKB	2.41	2.06
			92	S	AGC/PKC/Eta/PKCh	4.25	3.75

## Conclusion

In this study, we employed comparative proteomic analyses of cellular protein profiles during thapsigargin-induced INS-1 rat insulinoma cell death in the absence and presence of exenatide, a GLP-1 receptor agonist, in INS-1 cell line, to identify the proteins involved in ER stress caused INS-1 cell death and its protection by exenatide. We found that thapsigargin caused a variety of alterations in a number of cellular proteins involved in metabolic processes and protein folding. These alterations were reversed by exenatide treatment. While most of the proteins up- and down-regulated by thapsigargin can be assumed to be involved in ER stress, our most significant finding relevant to INS-1 cell apoptosis is the appearance of modified spots of heat shock proteins, thimet oligopeptidase and three 14–3–3 protein isomers, 14–3–3β, ε, and θ. Treatment with exenatide completely blocked their appearance, suggesting that modifications of these proteins are major events in ER-stress induced INS-1 cell death. We further found that various other modifications including phosphorylation of 14–3–3 isoforms precede its appearance and promotion of INS-1 cell death. This study provides the following insights into INS-1 cell death during ER stress and the anti-apoptotic effect of exenatide: 1) Phosphorylations of 14–3–3 protein may be critical modification in the process of ER stress-induced INS-1 cell death. 2) Alteration of 14–3–3 phosphorylation status by exenatide may play a key role in its INS-1 cell protective effect. 3) 14–3–3 and thimet oligopeptidase are selectively modified in response to INS-1 cell death caused by thapsigargin and restored by exenatide, caused prevention of INS-1 cell death in ER stress. This study is the first to report on the possible involvement of 14–3–3 and thimet oligopeptidase and their PTMs in the survival and function of INS-1 rat insulinoma cells. Our finding that phosphorylation of 14–3–3β, ε and to the appearance of 14–3–3 protein spots after thapsigargin treatment, warrants further studies to confirm and extend the role of phosphorylations in 14–3–3 in ER stress and its protection. Also needed is further information identifying the interacting partners of these phosphorylated proteins, their upstream cognate kinases and their mutants that could provide further insights into the mechanisms underlying ER stress induced INS-1 cell death and anti-apoptotic effect of exenatide. These studies pave the way for further understanding of the role of ER stress in diseases associated with protein folding.

## Supporting Information

S1 Fig Changes of multiple spots of same protein by thapsigargin alone or thapsigargin plus exenatide. Left panel indicates quantitative amount of each spot on 2D-PAGE stained with silver staining (right panel).
(PDF)

S2 Fig MS/MS spectra of oxidized thimet oligopeptidase. (PDF)

S3 Fig MS/MS spectrum of phosphorylated 14–3–3β/α. (PDF)

S4 Fig MS/MS spectra of phosphorylated 14–3–3ε. (PDF)

S5 Fig MS/MS spectrum of phosphorylated 14–3–3θ. (PDF)

S6 Fig MS/MS spectra of phosphorylated 14–3–3γ. (PDF)

S7 Fig MS/MS spectra of phosphorylated 14–3–3δ/ζ. (PDF)

S1 Table List of primer sequence and polymerase chain reaction conditions for rat target genes. (PDF)

S2 Table Comparative proteomic analysis of protein alterations during thapsigargin-induced beta cell death. Total protein spots significantly altered during thapsigargin-induced beta cell death are listed in ***the order of spot number*.**
(PDF)

S3 Table Classification of 18 protein spots whose thapsigargin-induced changes were unaffected by exenatide treatment. (PDF)

S4 Table Classification of 8 protein spots whose thapsigargin-induced changes are augmented by exenatide treatment. (PDF)

S5 Table Classification of 32 protein spots whose thapsigargin-induced changes are reversed by exenatide treatment. (PDF)

S6 Table List of peptide sequences for each protein assignment. (PDF)
