# Supplementary material for: Proteomic Analysis of INS-1 Rat Insulinoma Cells: ER Stress Effects and the Protective Role of Exenatide, a GLP-1 Receptor Agonist
Source: PLoS One. 2015 Mar 20;10(3):e0120536. doi: 10.1371/journal.pone.0120536 (PMC4368701; doi:10.1371/journal.pone.0120536)

Figure S1. Changes of multiple spots of same protein by thapsigargin alone or thapsigargin plus exenatide

A. Heat shock cognate 71 kDa protein

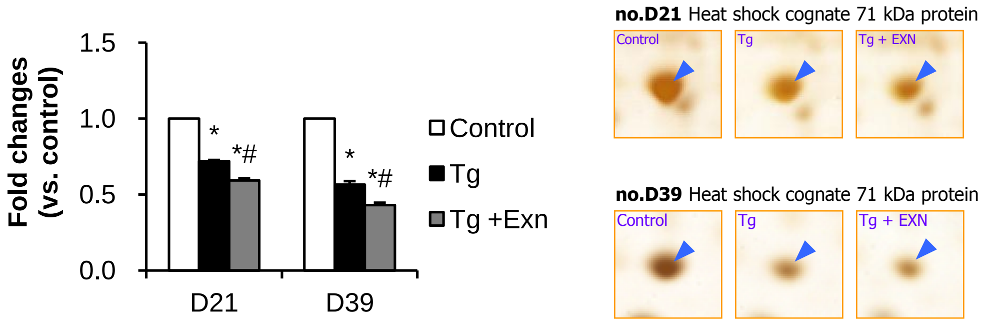

B. Heat shock protein HSP 90-β

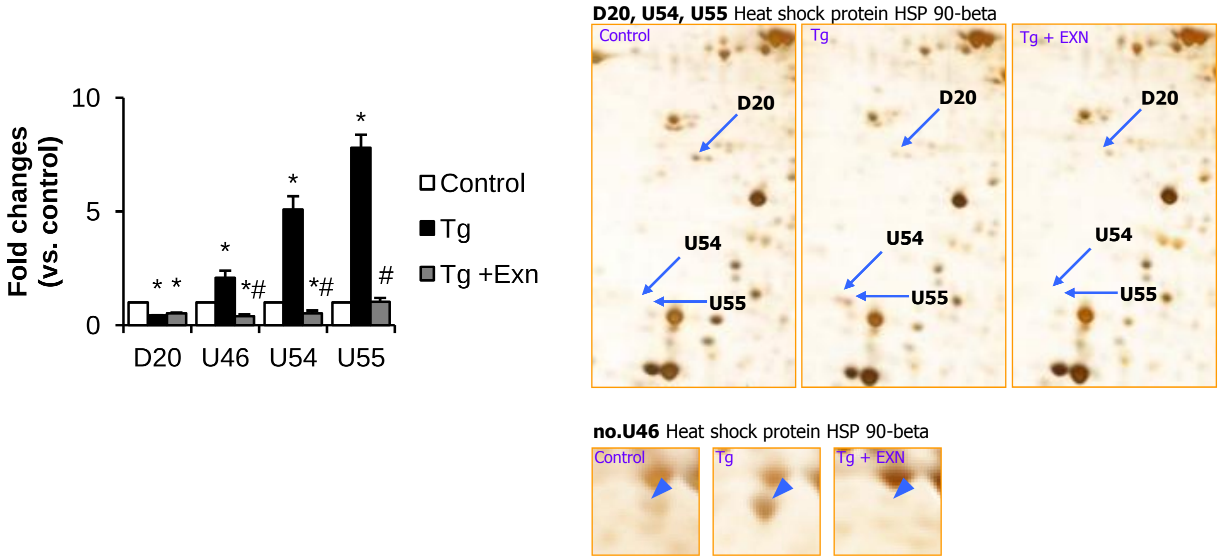

C. Heat shock protein 105 kDa

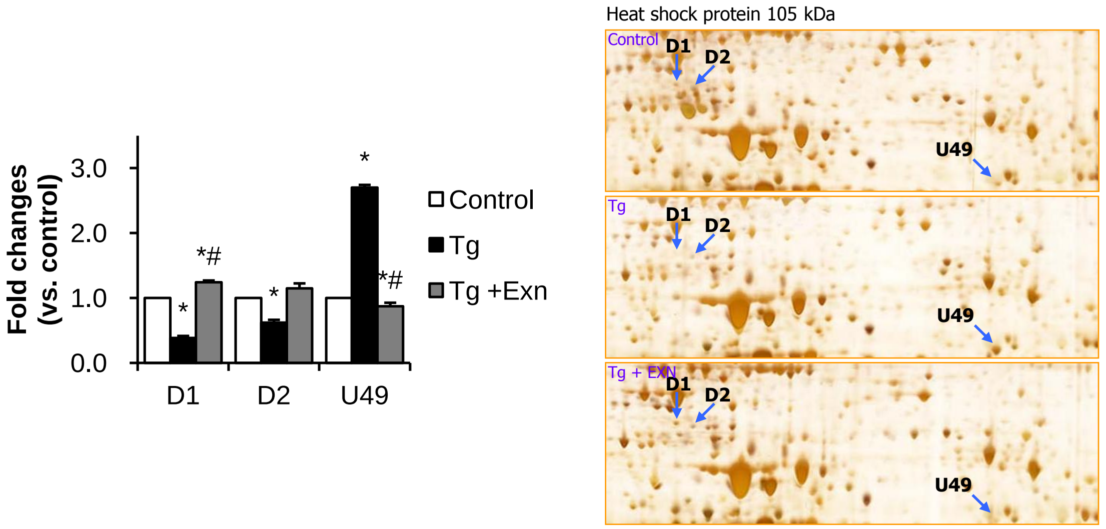

Figure S1. Changes of multiple spots of same protein by thapsigargin alone or thapsigargin plus exenatide *(continued)*

D. ATP synthase subunit β, mitochondrial

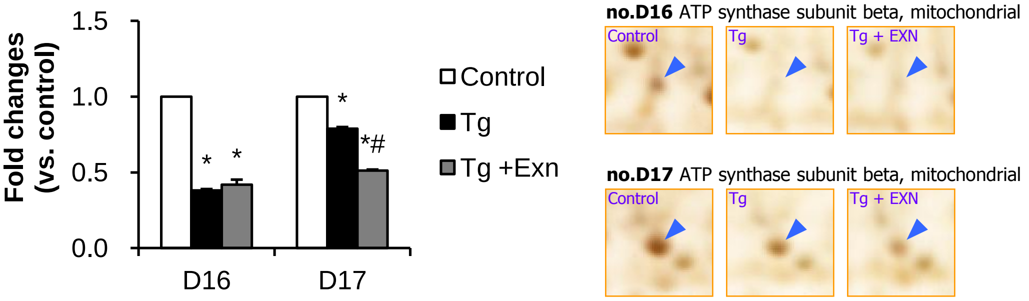

E. α-centractin

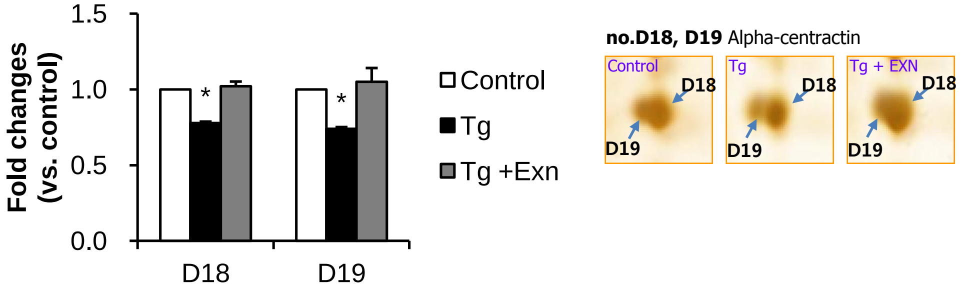

F. Heterogeneous nuclear ribonucleoprotein D-like

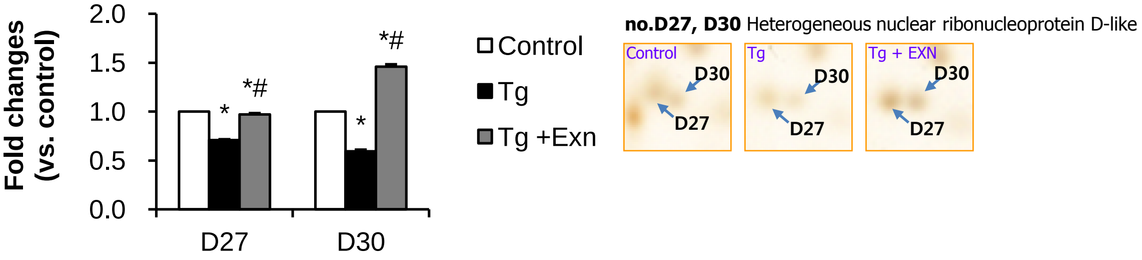

G. Nucleophosmin

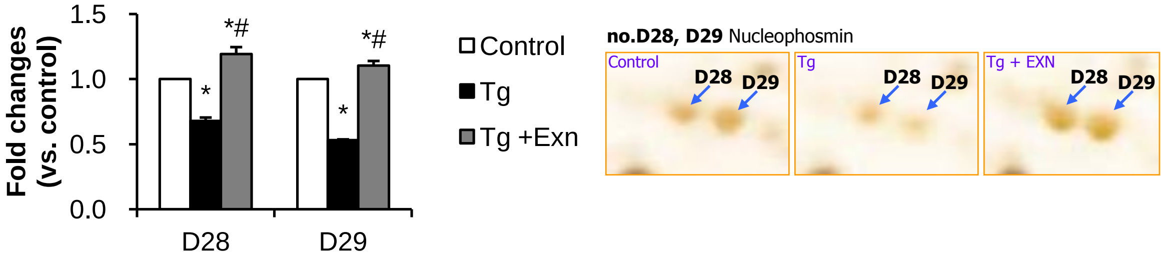

Figure S1. Changes of multiple spots of same protein by thapsigargin alone or thapsigargin plus exenatide *(continued)*

H. Glycerol-3-phosphate dehydrogenase [NAD+], cytoplasmic

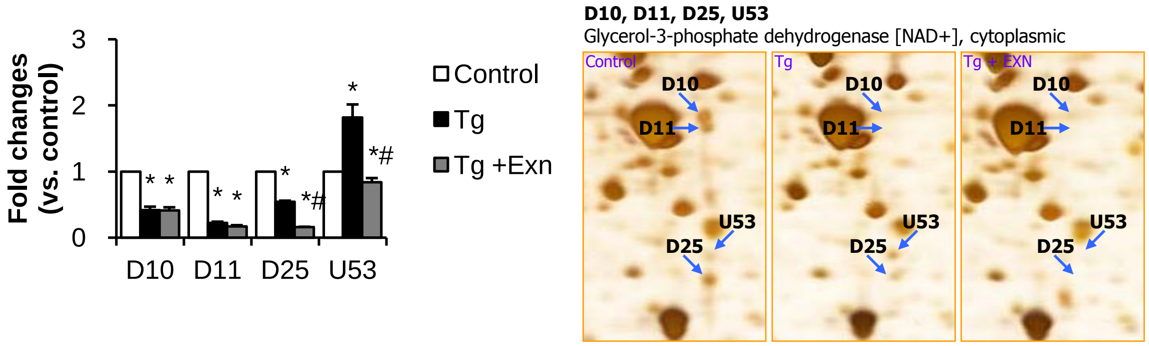

I. Ubiquilin-1

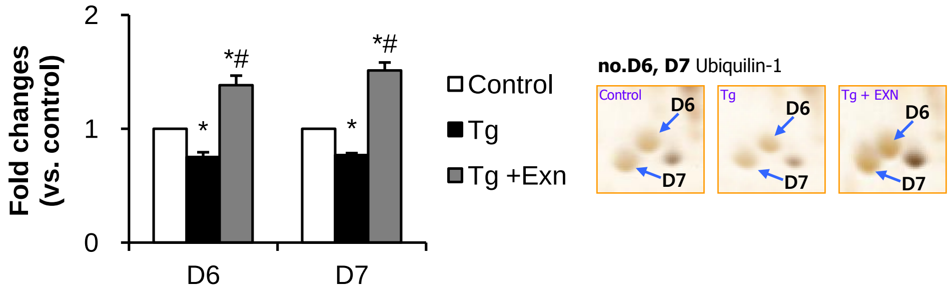

J. Tubulin alpha-1B chain

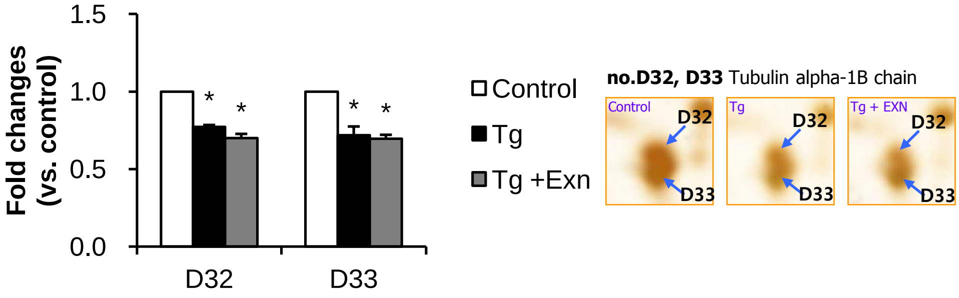

Supplement: S1 Fig — Left panel indicates quantitative amount of each spot on 2D-PAGE stained with silver staining (right panel). (PDF) [file pone.0120536.s001.pdf]
