# Supplementary material for: Proteomic Analysis of INS-1 Rat Insulinoma Cells: ER Stress Effects and the Protective Role of Exenatide, a GLP-1 Receptor Agonist
Source: PLoS One. 2015 Mar 20;10(3):e0120536. doi: 10.1371/journal.pone.0120536 (PMC4368701; doi:10.1371/journal.pone.0120536)

Figure S2. MS/MS spectra of oxidized thimet oligopeptidase

Thimet oligopeptidase

^{171}LSLLCIDFNK^{180} 175C -16Da conversion of Cys to Ser at spot D4 and U47

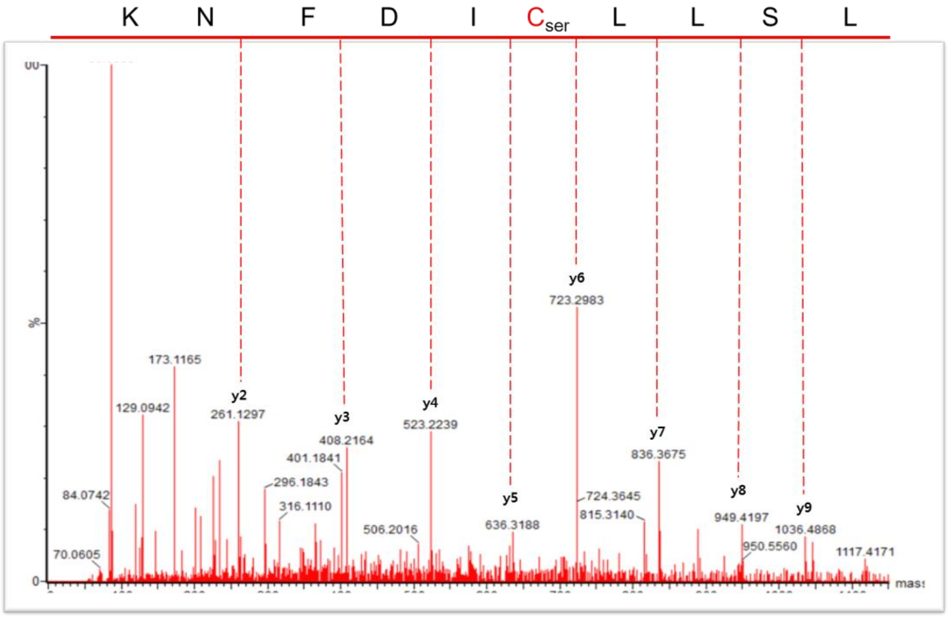

Thimet oligopeptidase

^{171}LSLLCIDFNK^{180} 175C -34Da conversion of Cys to DHA at spot D4 and U47

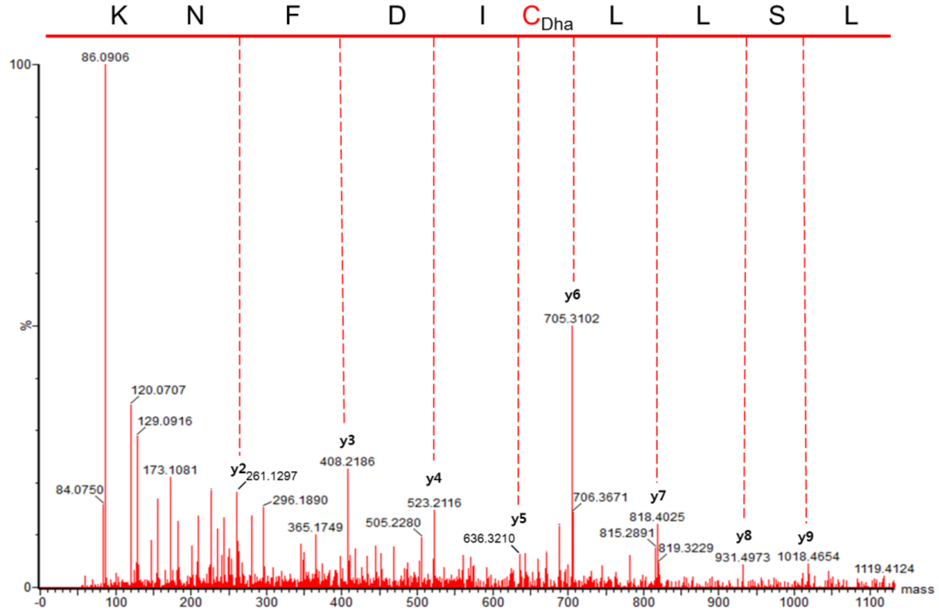

Supplement: S2 Fig — (PDF) [file pone.0120536.s002.pdf]
