# Supplementary figures and images for: Proteomic Analysis of INS-1 Rat Insulinoma Cells: ER Stress Effects and the Protective Role of Exenatide, a GLP-1 Receptor Agonist
Source: PLoS One. 2015 Mar 20;10(3):e0120536. doi: 10.1371/journal.pone.0120536 (PMC4368701; doi:10.1371/journal.pone.0120536)

Figure S3. MS/MS spectrum of phosphorylated 14-3-3β/α

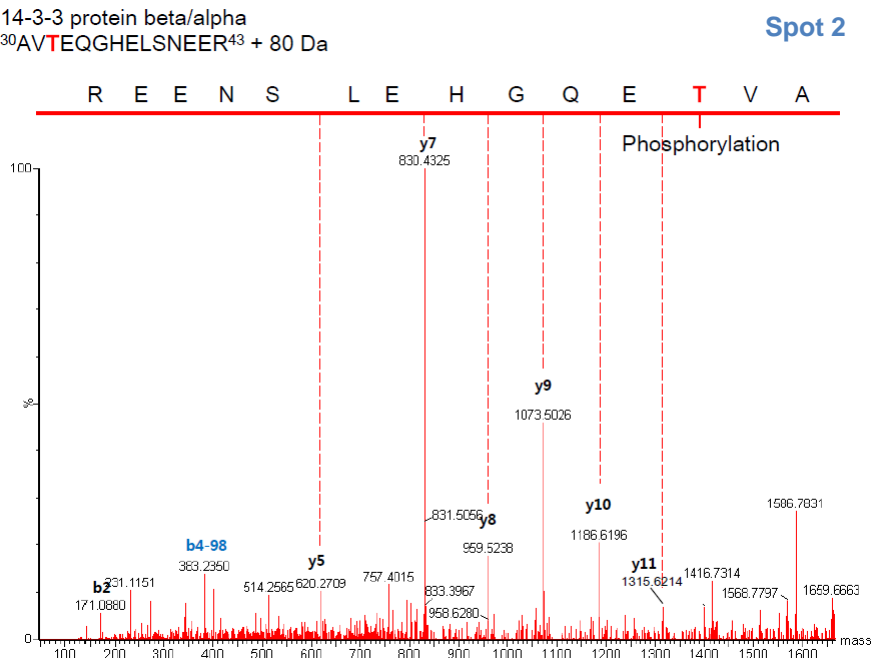

Supplement: S3 Fig — (PDF) [file pone.0120536.s003.pdf]

Figure S5. MS/MS spectrum of acetylated 14-3-3θ

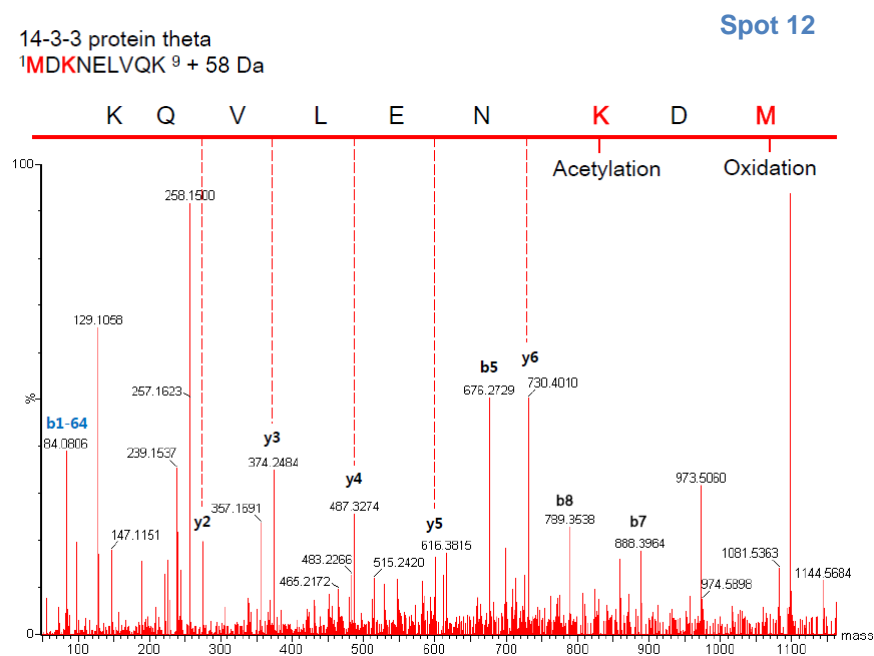

Supplement: S5 Fig — (PDF) [file pone.0120536.s005.pdf]

Figure S6. MS/MS spectra of phosphorylated 14-3-3γ

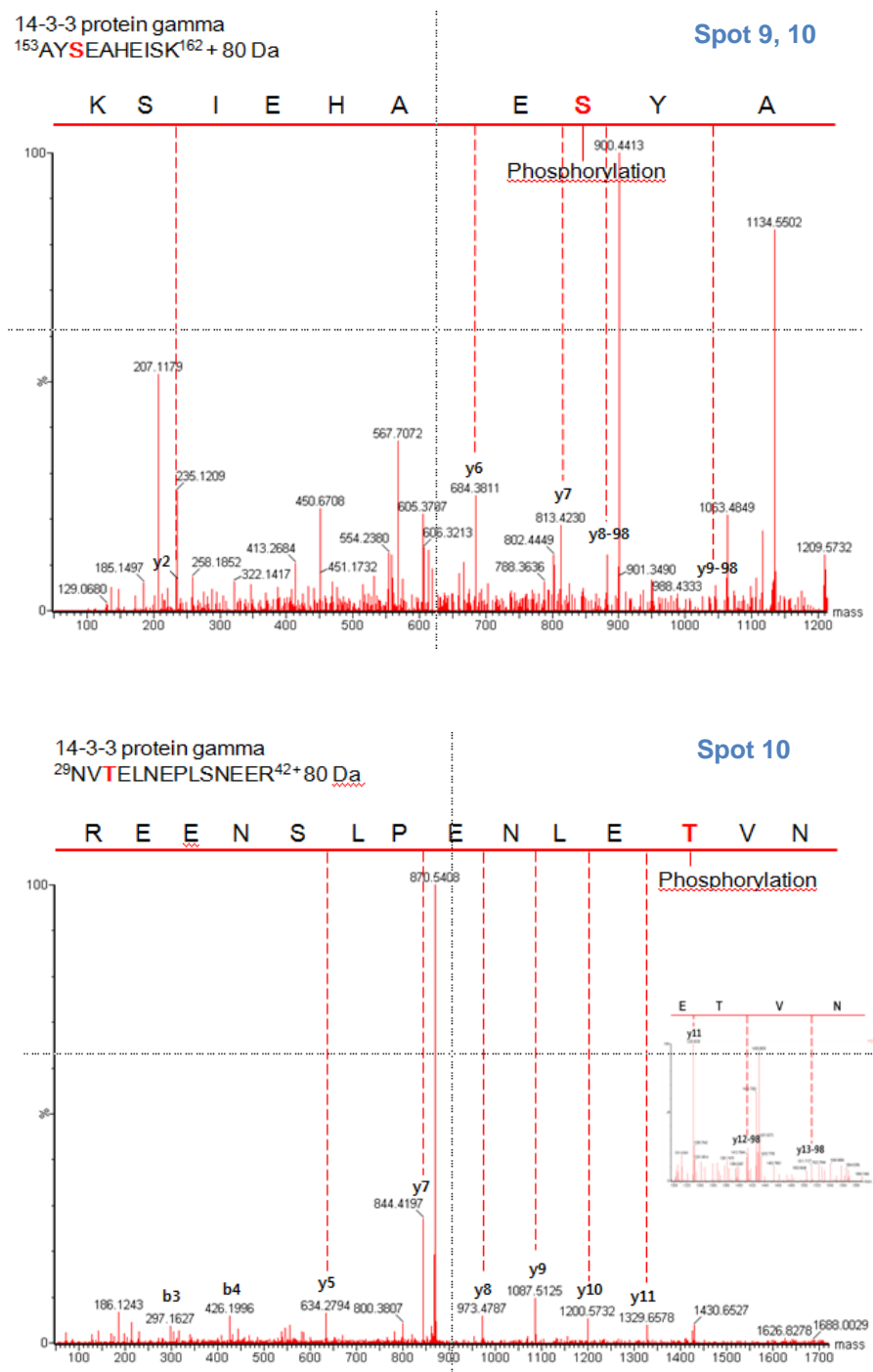

Supplement: S6 Fig — (PDF) [file pone.0120536.s006.pdf]

Figure S7. MS/MS spectra of phosphorylated 14-3-3  $\delta/\zeta$

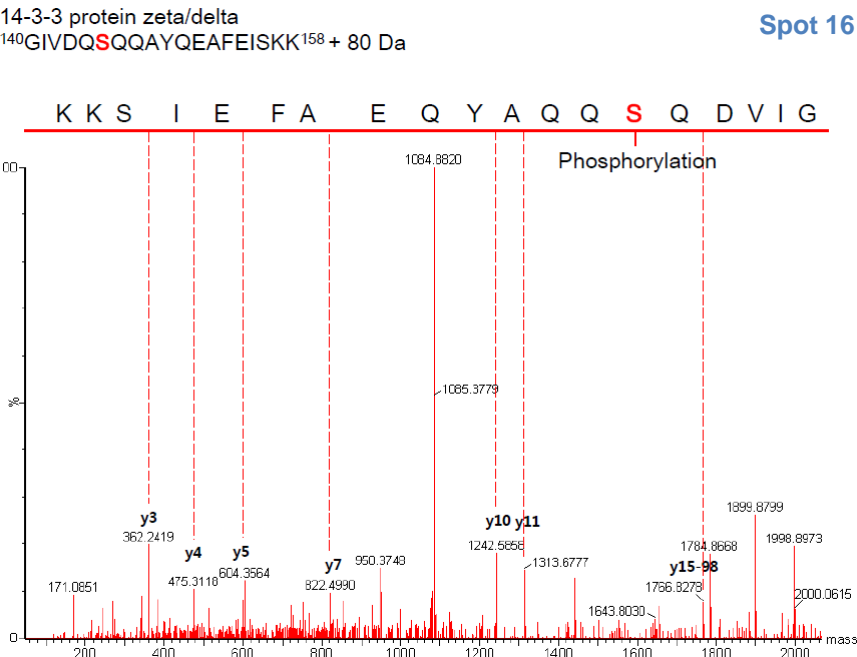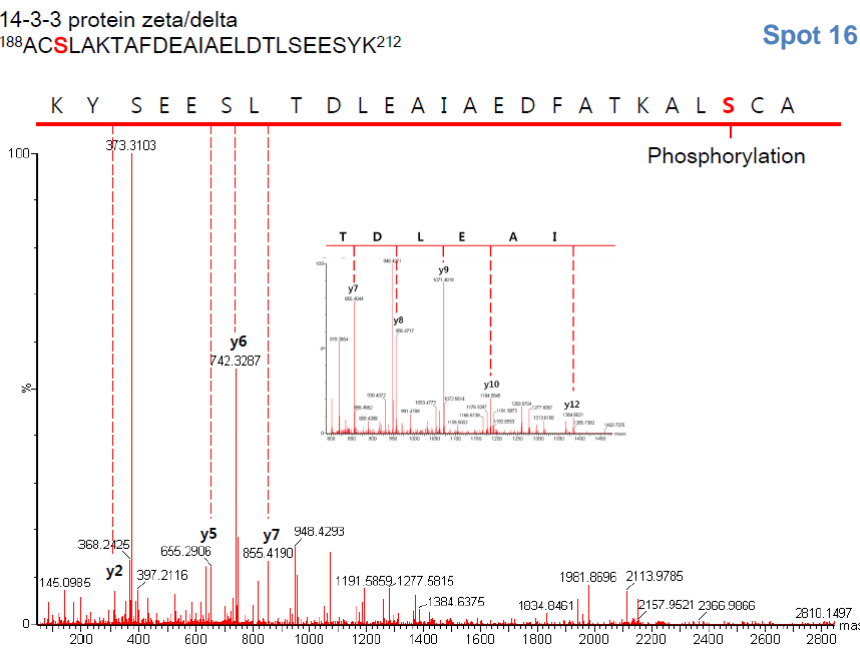

Supplement: S7 Fig — (PDF) [file pone.0120536.s007.pdf]
