# Supplementary material for: Proteomic Analysis of INS-1 Rat Insulinoma Cells: ER Stress Effects and the Protective Role of Exenatide, a GLP-1 Receptor Agonist
Source: PLoS One. 2015 Mar 20;10(3):e0120536. doi: 10.1371/journal.pone.0120536 (PMC4368701; doi:10.1371/journal.pone.0120536)

Figure S4. MS/MS spectra of phosphorylated 14-3-3ε

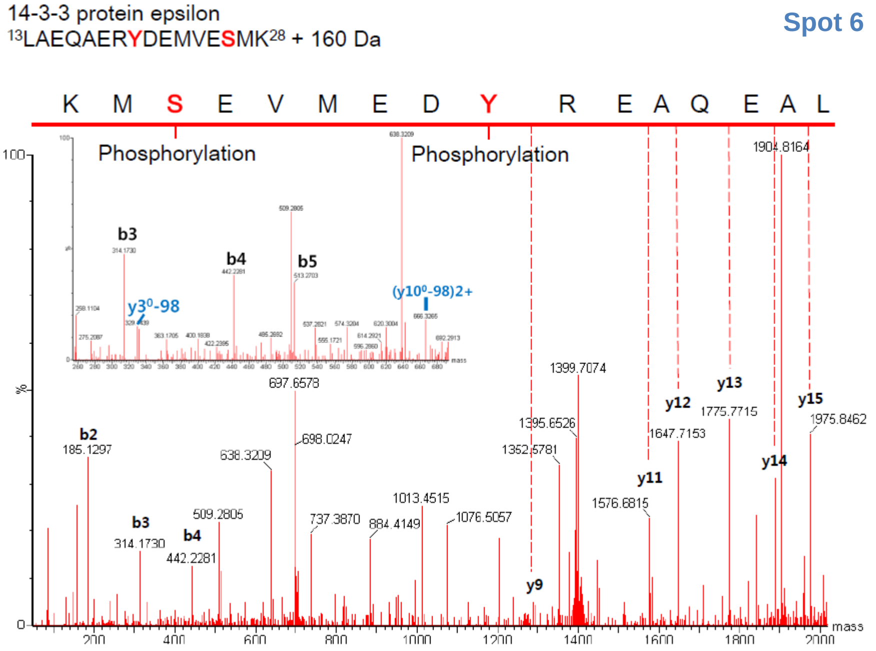

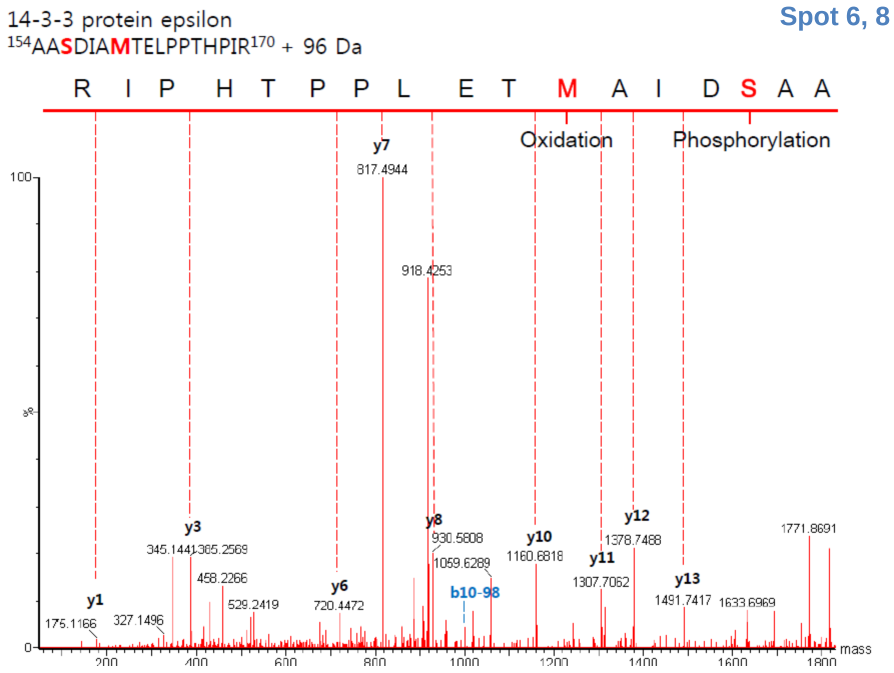

Supplement: S4 Fig — (PDF) [file pone.0120536.s004.pdf]
