# Supplementary material for: Proteomic Analysis of INS-1 Rat Insulinoma Cells: ER Stress Effects and the Protective Role of Exenatide, a GLP-1 Receptor Agonist
Source: PLoS One. 2015 Mar 20;10(3):e0120536. doi: 10.1371/journal.pone.0120536 (PMC4368701; doi:10.1371/journal.pone.0120536)
Supplement: S1 Table — (PDF) [file pone.0120536.s008.pdf]

Table S1. List of primer sequences and polymerase chain reaction conditions for rat target genes

Target gene	***Forward***	***Reward***	***Anneal. Temp.***	***Mg^{2+} (mM)***	***Cycle No.***
TXNIP	5'-atcccagacaccccagaag-3'	5'-tcaagcagagaggcagaaag-3'	63℃	1.5	27
IRS-2	5'-tcgccacctcgtcgaagactct-3'	5'-gacaggtgtgtggggacagcg-3'	63℃	1.5	25
Bip	5'-ccaccaggatgcagacattg-3'	5'-agggcctccacttccataga-3'	57℃	2.5	24
CHOP	5'-ccagcagaggtcacaagcac-3'	5'-cgcactgaccactctgtttc-3'	57℃	2.5	27
GLP1R	5'-gggtgctgttgacctgaaat-3'	5'-gtggggtgagagaaaccaga-3'	60℃	2.5	27
β actin	5'-ggctgtgttgtccctgtatg-3'	5'-aggaaggaaggctggaagag-3'	63℃	1.5	27
