# Supplementary material for: Proteomic Analysis of INS-1 Rat Insulinoma Cells: ER Stress Effects and the Protective Role of Exenatide, a GLP-1 Receptor Agonist
Source: PLoS One. 2015 Mar 20;10(3):e0120536. doi: 10.1371/journal.pone.0120536 (PMC4368701; doi:10.1371/journal.pone.0120536)
Supplement: S3 Table — (PDF) [file pone.0120536.s010.pdf]

Table S3.Classification of 18 protein spots which were unchanged after exenatide treatment

Spot no.	Mascot score	Accession no.	Queries matched	Protein name	Mass	pI	Fold difference			
							Co.	Tg	Tg+Exn	*P value*
Protein metabolic process										
**D13**	269	Q3B8Q2	11	**Eukaryotic initiation factor 4A-III**	46811	6.30	1.00	0.670	1.000	0.210
**D14**	179	Q68FR6	10	**Elongation factor 1-gamma**	50029	6.31	1.00	0.647	0.764	0.081
**D15**	256	P62193	15	**26S protease regulatory subunit 4**	49154	5.87	1.00	0.568	0.487	0.050
**D36**	197	P19945	10	**60S acidic ribosomal protein P0**	34194	5.91	1.00	0.512	0.556	0.594
**U52**	308	Q01205	12	**Dihydrolipoyllysine-residue succinyltransferase component of 2-oxoglutarate dehydrogenase complex, mitochondrial**	48894	8.89	1.00	1.434	1.381	0.585
Cellular organization										
**D22**	64	P62738	3	**Actin, aortic smooth muscle**	41982	5.24	1.00	0.352	0.273	0.059
**D32**	414	Q6P9V9	25	**Tubulin alpha-1B chain**	50120	4.94	1.00	0.772	0.699	0.082
**D33**	414	Q6P9V9	25	**Tubulin alpha-1B chain**	50120	4.94	1.00	0.718	0.695	0.735
Protein folding										
**D20**	410	P34058	20	**Heat shock protein HSP 90-beta**	83229	4.97	1.00	0.443	0.518	0.057
Transport										
**D16**	69	P10719	2	**ATP synthase subunit beta, mitochondrial**	56318	5.18	1.00	0.380	0.420	0.244
**D18**	70	P85515	1	**Alpha-centractin**	42587	6.19	1.00	0.777	1.020	0.077
**D19**	239	P85515	9	**Alpha-centractin**	42587	6.19	1.00	0.740	1.050	0.079
Lipid metabolic process										
**D10**	348	O35077	17	**Glycerol-3-phosphate dehydrogenase [NAD+], cytoplasmic**	37428	6.16	1.00	0.418	0.410	0.924
**D11**	146	O35077	11	**Glycerol-3-phosphate dehydrogenase [NAD+], cytoplasmic**	37428	6.16	1.00	0.222	0.167	0.151
Tricarboxylic acid cycle										
**D12**	139	P16638	11	**ATP-citrate synthase**	120559	6.96	1.00	0.340	0.489	0.275
**D34**	278	P42123	19	**L-lactate dehydrogenase B chain**	36589	5.70	1.00	0.755	0.821	0.264
Apoptosis										
**U45**	448	Q9QZA2	16	**Programmed cell death 6-interacting protein**	96570	6.15	1.00	1.467	1.522	0.438
Carbohydrate metabolic process										
**D35**	239	P11980	8	**Pyruvate kinase isozymes M1/M2**	57781	6.63	1.00	0.568	0.438	0.080
