# Supplementary material for: Proteomic Analysis of INS-1 Rat Insulinoma Cells: ER Stress Effects and the Protective Role of Exenatide, a GLP-1 Receptor Agonist
Source: PLoS One. 2015 Mar 20;10(3):e0120536. doi: 10.1371/journal.pone.0120536 (PMC4368701; doi:10.1371/journal.pone.0120536)
Supplement: S4 Table — (PDF) [file pone.0120536.s011.pdf]

Table S4. Classification of 8 protein spots which were augmented by exenatide

Spot no.	Mascot score	Accession no.	Queries matched	Protein name	Mass	pI	Fold difference			
							Co.	Tg	Tg+Exn	*P value*
Carbohydrate metabolic process										
**D44**	564	P25113	35	**Phosphoglyceratemutase 1**	28814	6.67	1.00	0.740	0.369	<0.05
Cellular organization										
**D23**	186	P63259	10	**Actin, cytoplasmic 2**	41766	5.31	1.00	0.432	0.312	<0.05
Lipid metabolic process										
**D25**	351	O35077	16	**Glycerol-3-phosphate dehydrogenase [NAD+], cytoplasmic**	37428	6.16	1.00	0.540	0.157	<0.05
Protein folding										
**D9**	686	P18418	43	**Calreticulin**	47966	4.33	1.00	0.649	0.384	<0.05
**D21**	299	P63018	14	**Heat shock cognate 71 kDa protein**	70827	5.37	1.00	0.719	0.593	<0.05
**D39**	178	P63018	16	**Heat shock cognate 71 kDa protein**	70827	5.37	1.00	0.564	0.430	<0.05
Protein metabolic process										
**D40**	156	P38983	6	**40S ribosomal protein SA**	32803	4.80	1.00	0.529	0.438	<0.05
Transport										
**D17**	323	P10719	11	**ATP synthase subunit beta, mitochondrial**	56318	5.18	1.00	0.788	0.513	<0.05
