# Supplementary material for: Proteomic Analysis of INS-1 Rat Insulinoma Cells: ER Stress Effects and the Protective Role of Exenatide, a GLP-1 Receptor Agonist
Source: PLoS One. 2015 Mar 20;10(3):e0120536. doi: 10.1371/journal.pone.0120536 (PMC4368701; doi:10.1371/journal.pone.0120536)
Supplement: S5 Table — (PDF) [file pone.0120536.s012.pdf]

Table S5. Classification of 32 protein spots which were reversed by exenatide treatment (N=3)

Spot no.	Mascot score	Accession no.	Queries matched	Protein name	Mass	pI	Fold difference			*P value*
							Co.	Tg	Tg+Exn	
Protein metabolic process										
**D3**	409	P24155	15	**Thimetoligopeptidase**	78335	5.64	1.00	0.178	1.020	<0.05
**D4**	587	P24155	26	**Thimetoligopeptidase**	78335	5.64	1.00	0.300	1.214	<0.05
**U47**	385	P24155	20	**Thimetoligopeptidase**	78335	5.64	1.00	3.722	0.490	<0.05
**D6**	381	Q9JJP9	12	**Ubiquilin-1**	62032	4.87	1.00	0.752	1.383	<0.05
**D7**	179	Q9JJP9	11	**Ubiquilin-1**	62032	4.87	1.00	0.770	1.514	<0.05
**D8**	425	Q64303	15	**Serine/threonine-protein kinase PAK 2**	57924	5.57	1.00	0.570	1.251	<0.05
**D31**	640	B0BNA7	26	**Eukaryotic translation initiation factor 3 subunit I**	36438	5.38	1.00	0.734	0.857	<0.05
**D43**	324	P18422	18	**Proteasome subunit alpha type-3**	28401	5.29	1.00	0.672	1.079	<0.05
Protein folding										
**D1**	446	Q66HA8	21	**Heat shock protein 105 kDa**	96357	5.40	1.00	0.383	1.240	<0.05
**D2**	94	Q66HA8	3	**Heat shock protein 105 kDa**	96357	5.40	1.00	0.622	1.150	0.199
**U49**	62	Q66HA8	2	**Heat shock protein 105 kDa**	96357	5.40	1.00	2.703	0.872	<0.05
**U46**	203	P34058	8	**Heat shock protein HSP 90-beta**	83229	4.97	1.00	2.084	0.401	<0.05
**U54**	133	P34058	6	**Heat shock protein HSP 90-beta**	83229	4.97	1.00	5.087	0.521	<0.05
**U55**	313	P34058	16	**Heat shock protein HSP 90-beta**	83229	4.97	1.00	7.797	1.030	<0.05
Nucleobase, nucleoside, nucleotide and nucleic acid metabolic process										
**D5**	201	Q4V7C6	14	**GMP synthase**	76709	6.21	1.00	0.464	1.235	< 0.05
**D27**	103	Q3SWU3	2	**Heterogeneous nuclear ribonucleoprotein D-like**	35272	9.14	1.00	0.710	0.971	< 0.05
**D30**	165	Q3SWU3	5	**Heterogeneous nuclear ribonucleoprotein D-like**	35272	9.14	1.00	0.593	1.458	< 0.05
**D28**	418	P13084	12	**Nucleophosmin**	32540	4.62	1.00	0.680	1.191	< 0.05
**D29**	547	P13084	20	**Nucleophosmin**	32540	4.62	1.00	0.530	1.103	<0.05
**D41**	113	Q5M827	7	**Pirin**	32158	6.22	1.00	0.616	0.774	<0.05

Table S5. Classification of 32 protein spots which were reversed by exenatide treatment (*continued*)

Spot no.	Mascot score	Accession no.	Queries matched	Protein name	Mass	pI	Fold difference			
							Co.	Tg	Tg+Exn	*P value*
Signal transduction										
**U56**	238	P35213	11	**14-3-3 protein beta/alpha**	28037	4.81	1.00	15.693	0.619	<0.05
**U57**	205	P68255	8	**14-3-3 protein theta**	27761	4.69	1.00	7.464	0.827	<0.05
**U58**	317	P62260	22	**14-3-3 protein epsilon**	29155	4.63	1.00	13.220	0.672	<0.05
Carbohydrate metabolic process										
**D37**	346	P07943	13	**Aldose reductase**	35774	6.26	1.00	0.709	1.060	0.064
**D38**	138	P54311	5	**Guanine nucleotide-binding protein G(I)/G(S)/G(T) subunit beta-1**	37353	5.60	1.00	0.745	0.897	<0.05
Cellular organization										
**U48**	116	Q62871	6	**Cytoplasmic dynein 1 intermediate chain 2**	71134	5.11	1.00	3.407	1.080	<0.05
**U50**	331	P68370	14	**Tubulin alpha-1A chain**	50104	4.94	1.00	3.380	1.100	<0.05
**U51**	361	P68370	21	**Tubulin alpha-1A chain**	50104	4.94	1.00	6.029	0.806	<0.05
Lipid metabolic process										
**D42**	64	Q8CIN7	2	**Inositol monophosphatase 2**	31776	5.68	1.00	0.555	1.020	0.103
**U53**	48	O35077	4	**Glycerol-3-phosphate dehydrogenase [NAD+], cytoplasmic**	37428	6.16	1.00	1.816	0.840	<0.05
Biological process unclassified										
**D24**	50	P04764	1	**Alpha-enolase**	47098	6.16	1.00	0.296	1.000	<0.05
**D26**	206	Q6JE36	6	**Protein NDRG1**	42927	5.77	1.00	0.377	0.793	<0.05
