# Supplementary material for: Proteomic Analysis of INS-1 Rat Insulinoma Cells: ER Stress Effects and the Protective Role of Exenatide, a GLP-1 Receptor Agonist
Source: PLoS One. 2015 Mar 20;10(3):e0120536. doi: 10.1371/journal.pone.0120536 (PMC4368701; doi:10.1371/journal.pone.0120536)
Supplement: S6 Table — (PDF) [file pone.0120536.s013.pdf]

Table S6. List of peptide sequences for each protein assignment

**Spot D1**	HS105_RAT Mass: 96357 Score: 446 Queries matched: 21 emPAI: 0.69<br>Heat shock protein 105 kDa OS=Rattus norvegicus GN=Hsph1 PE=1 SV=1							
Observed	Mr(expt)	Mr(calc)	Delta	Miss	Score	Expect	Rank	Peptide
560.2860	1118.5574	1118.6084	-0.0510	0	(35)	0.026	1	R.FVVQNVSAQK.D
560.2861	1118.5576	1118.6084	-0.0508	0	35	0.025	1	R.FVVQNVSAQK.D
580.3246	1158.6346	1158.6244	0.0102	1	45	0.0024	1	K.LKETAENNLK.K
641.8284	1281.6422	1281.6313	0.0109	0	102	4.8e-09	1	K.QDLPNADEKPR.V
654.8403	1307.6660	1307.6431	0.0229	0	(16)	2	1	K.ENLSYDLVPMK.N
661.3521	1320.6896	1320.7078	-0.0182	0	66	1.9e-05	1	K.VLGTAFDPFLGGK.N
662.8366	1323.6586	1323.6380	0.0206	0	30	0.078	1	K.ENLSYDLVPMK.N + Oxidation (M)
731.3553	1460.6960	1460.7107	-0.0146	0	68	1.4e-05	1	K.DVSTTLNADEAVAR.G
740.3460	1478.6774	1478.7001	-0.0227	0	20	0.78	1	R.AGGIETIANEFSDR.C
766.3929	1530.7712	1530.7889	-0.0177	0	85	2.3e-07	1	K.TEEVSAIEIVGGATR.I
780.3603	1558.7060	1558.6664	0.0396	0	(1)	68	8	R.AGGIETIANEFSDR.C + Phospho (ST)
791.4116	1580.8086	1580.7756	0.0331	1	75	2.7e-06	1	K.EKENLSYDLVPMK.N + Oxidation (M)
795.9721	1589.9296	1589.8817	0.0479	1	61	5.6e-05	1	K.LKVLGTAFDPFLGGK.N + Formyl (K)
831.3931	1660.7716	1660.7419	0.0297	1	(37)	0.02	1	K.EKENLSYDLVPMK.N + Oxidation (M); Phospho (ST)
563.6222	1687.8448	1687.8278	0.0170	0	21	0.68	1	K.NQQITHANNTVSSFK.R
916.9717	1831.9288	1831.8848	0.0440	0	(28)	0.15	1	R.LMNDMTAVALNYGIYK.Q + Oxidation (M)
924.9473	1847.8800	1847.8797	0.0003	0	70	8.8e-06	1	R.LMNDMTAVALNYGIYK.Q + 2 Oxidation (M)
965.9721	1929.9296	1929.8907	0.0389	0	19	1.1	1	R.VNTHGIFTISTASMVEK.V + Oxidation (M); Phospho (ST)
1096.0347	2190.0548	2190.0068	0.0480	0	22	0.48	1	K.KPVTDCVISVPSFFTDAER.R + Phospho (ST)
1178.0725	2354.1304	2354.1107	0.0198	0	17	1.5	1	R.GPFELEAFYSDPQAVPYPEAK.I
785.7247	2354.1523	2354.1107	0.0416	0	(9)	10	1	R.GPFELEAFYSDPQAVPYPEAK.I

**Spot D2**	HS105_RAT Mass: 96357 Score: 94 Queries matched: 3 emPAI: 0.08<br>Heat shock protein 105 kDa OS=Rattus norvegicus GN=Hsph1 PE=1 SV=1							
Observed	Mr(expt)	Mr(calc)	Delta	Miss	Score	Expect	Rank	Peptide
713.3480	1424.6814	1424.6857	-0.0043	0	49	0.0012	1	R.DLLNMYIETEGK.M
766.4022	1530.7898	1530.7889	0.0009	0	75	2.5e-06	1	K.TEEVSAIEIVGGATR.I
1084.9965	2167.9784	2167.9797	-0.0012	0	9	11	1	R.LLTETEDWLYEEGEDQAK.Q

**Spot D3**	THOP1_RAT Mass: 78335 Score: 409 Queries matched: 15 emPAI: 0.91<br>Thimet oligopeptidase OS=Rattus norvegicus GN=Thop1 PE=1 SV=4							
Observed	Mr(expt)	Mr(calc)	Delta	Miss	Score	Expect	Rank	Peptide
543.7847	1085.5548	1085.5546	0.0003	0	29	0.1	1	K.FYLDLYPR.E
552.3024	1102.5902	1102.5883	0.0019	0	42	0.005	1	R.QANAGLFNLR.Q
558.7969	1115.5792	1115.5723	0.0069	0	42	0.0042	1	R.WDLSAQQIR.A
623.3529	1244.6912	1244.6976	-0.0064	0	54	0.00033	1	R.ALTTQLIEQTK.C
629.8142	1257.6138	1257.6088	0.0050	0	56	0.00019	1	R.TGGEAPEDLLEK.L
643.3193	1284.6240	1284.6173	0.0068	0	41	0.0063	1	K.EYFPMQVVTR.G + Oxidation (M)
666.8007	1331.5868	1331.5816	0.0053	0	65	2.6e-05	1	R.YYMNQVEETR.Y
732.8936	1463.7726	1463.7620	0.0106	0	53	0.00039	1	K.ALADVEVTYTVQR.N
754.9016	1507.7886	1507.7783	0.0103	0	56	0.00021	1	R.NILDFPQHVSPNK.D
776.4026	1550.7906	1550.7940	-0.0034	0	67	1.6e-05	1	K.TSQTVATFLDELAR.K
784.3904	1566.7662	1566.7678	-0.0016	0	59	0.00011	1	K.NLNEDTTFLPFTR.E
894.4208	1786.8270	1786.8261	0.0009	0	57	0.00019	1	R.VGAQDFEDVSYESTLK.A

Table S6. List of peptide sequences for each protein assignment

**Spot D3**	THOP1_RAT Mass: 78335 Score: 409 Queries matched: 15 emPAI: 0.91<br>Thimet oligopeptidase OS=Rattus norvegicus GN=Thop1 PE=1 SV=4							
Observed	Mr(expt)	Mr(calc)	Delta	Miss	Score	Expect	Rank	Peptide
895.4463	1788.8780	1788.8781	-0.0001	0	47	0.0018	1	R.EELGGLPEDFLNSLEK.T
1054.5072	2106.9998	2106.9721	0.0278	0	32	0.05	1	R.DFVEAPSQMLENWVWEK.E
1062.4963	2122.9780	2122.9670	0.0111	0	(22)	0.54	1	R.DFVEAPSQMLENWVWEK.E + Oxidation (M)

**Spot D4**	THOP1_RAT Mass: 78335 Score: 587 Queries matched: 26 emPAI: 1.76<br>Thimet oligopeptidase OS=Rattus norvegicus GN=Thop1 PE=1 SV=4							
Observed	Mr(expt)	Mr(calc)	Delta	Miss	Score	Expect	Rank	Peptide
461.2523	920.4900	920.4967	-0.0067	0	20	0.79	1	K.QDAFLLSK.G
543.7767	1085.5388	1085.5546	-0.0157	0	43	0.0035	1	K.FYLDLYPR.E
552.2899	1102.5652	1102.5883	-0.0231	0	54	0.00031	1	R.QANAGLFNLR.Q
558.7914	1115.5682	1115.5723	-0.0041	0	48	0.0013	1	R.WDLSAQQIR.A
566.8215	1131.6284	1131.6288	-0.0004	1	30	0.068	1	R.FKQEGVLSPK.V
589.3259	1176.6372	1176.6331	0.0041	0	2	51	2	K.YPHYFPLLK.K
623.3404	1244.6662	1244.6976	-0.0314	0	54	0.00033	1	R.ALTTQLIEQTK.C
629.8103	1257.6060	1257.6088	-0.0028	0	52	0.00051	1	R.TGGEAPEDLLEK.L
635.3204	1268.6262	1268.6223	0.0039	0	46	0.0021	1	K.EYFPMQVVTR.G
643.3113	1284.6080	1284.6173	-0.0092	0	(30)	0.081	1	K.EYFPMQVVTR.G + Oxidation (M)
666.7944	1331.5742	1331.5816	-0.0073	0	(35)	0.028	1	R.YYMNQVEETR.Y
672.3070	1342.5994	1342.5897	0.0097	0	(46)	0.0019	1	K.LSEFDVEMSMR.Q
674.7879	1347.5612	1347.5765	-0.0152	0	78	1.3e-06	1	R.YYMNQVEETR.Y + Oxidation (M)
680.2992	1358.5838	1358.5846	-0.0008	0	50	0.00086	1	K.LSEFDVEMSMR.Q + Oxidation (M)
732.8823	1463.7500	1463.7620	-0.0120	0	63	4.3e-05	1	K.ALADVEVTYTVQR.N
754.8992	1507.7838	1507.7783	0.0055	0	60	7.8e-05	1	R.NILDFPQHVSPNK.D
761.8594	1521.7042	1521.6956	0.0087	0	57	0.00017	1	R.THADYVLEMNMAK.T
776.3992	1550.7838	1550.7940	-0.0102	0	83	4.2e-07	1	K.TSQTVATFLDELAR.K
776.4121	1550.8096	1550.7940	0.0156	0	(14)	3.3	1	K.TSQTVATFLDELAR.K
784.3839	1566.7532	1566.7678	-0.0146	0	80	7.8e-07	1	K.NLNEDTTFLPFTR.E
794.8752	1587.7358	1587.7446	-0.0088	0	(15)	2.8	1	R.NILDFPQHVSPNK.D + Phospho (ST)
894.4105	1786.8064	1786.8261	-0.0197	0	57	0.00017	1	R.VGAQDFEDVSYESTLK.A
895.4461	1788.8776	1788.8781	-0.0005	0	47	0.0016	1	R.EELGGLPEDFLNSLEK.T
1054.4989	2106.9832	2106.9721	0.0112	0	32	0.051	1	R.DFVEAPSQMLENWVWEK.E
1062.4928	2122.9710	2122.9670	0.0041	0	(4)	34	1	R.DFVEAPSQMLENWVWEK.E + Oxidation (M)
729.3363	2184.9871	2185.0287	-0.0417	0	47	0.002	1	K.VDQVLHTQTDVDPAEEYAR.L

**Spot D5**	GUAA_RAT Mass: 76709 Score: 201 Queries matched: 14 emPAI: 0.46<br>GMP synthase [glutamine-hydrolyzing] OS=Rattus norvegicus GN=Gmps PE=1 SV=1							
Observed	Mr(expt)	Mr(calc)	Delta	Miss	Score	Expect	Rank	Peptide
446.7578	891.5010	891.5066	-0.0055	0	16	1.7	1	K.IIGDTFVK.I
518.7818	1035.5490	1035.5601	-0.0110	0	38	0.012	1	K.IVADFSASVK.K
581.3310	1160.6474	1160.6455	0.0020	0	39	0.0087	1	R.HPFPGPGLAIR.V
595.8010	1189.5874	1189.5979	-0.0104	0	24	0.34	1	K.DFPETNNILK.I
648.8232	1295.6318	1295.6258	0.0060	0	48	0.0014	1	R.QADFEAHNILR.E + Gln->pyro-Glu (N-term Q)
650.3379	1298.6612	1298.6718	-0.0105	0	47	0.0017	1	R.ELDLPEELVSR.H
777.9035	1553.7924	1553.7937	-0.0013	0	89	1e-07	1	K.EEIVLLTHGDSVDK.V
795.9052	1589.7958	1589.7726	0.0233	0	68	1.3e-05	1	K.DEPDWESLIFLAR.L

Table S6. List of peptide sequences for each protein assignment

**Spot D5**	GUAA_RAT Mass: 76709 Score: 201 Queries matched: 14 emPAI: 0.46<br>GMP synthase [glutamine-hydrolyzing] OS=Rattus norvegicus GN=Gmps PE=1 SV=1							
Observed	Mr(expt)	Mr(calc)	Delta	Miss	Score	Expect	Rank	Peptide
942.4431	1882.8716	1882.8659	0.0058	0	21	0.67	1	R.IMYDLTSKPPGTTEWE.- + Oxidation (M)
691.0528	2070.1366	2070.1183	0.0183	0	6	15	1	K.LMEITSQHSLNAFLLPIK.T + Oxidation (M)
1073.0715	2144.1284	2144.1365	-0.0081	0	45	0.002	1	K.EPPTDVTPTFLTTGVLSTLR.Q
715.7254	2144.1544	2144.1365	0.0178	0	(32)	0.044	1	K.EPPTDVTPTFLTTGVLSTLR.Q
1090.0884	2178.1622	2178.1612	0.0010	0	20	0.75	1	R.ELFVQSEIFPLETPAFAIK.E
921.4878	2761.4416	2761.4248	0.0167	0	12	4.2	1	R.TFITSDFMTGVPATPGNEIPVEVVLK.M

**Spot D6**	UBQL1_RAT Mass: 62032 Score: 381 Queries matched: 12 emPAI: 0.79<br>Ubiquilin-1 OS=Rattus norvegicus GN=Ubqln1 PE=1 SV=1							
Observed	Mr(expt)	Mr(calc)	Delta	Miss	Score	Expect	Rank	Peptide
619.7646	1237.5146	1237.5076	0.0071	0	44	0.0035	1	R.NPAMMQEMMR.N
720.9186	1439.8226	1439.8136	0.0090	0	38	0.011	1	K.SHIDQLVLIFAGK.I
869.9201	1737.8256	1737.8210	0.0047	0	22	0.53	1	K.EEFAVPENSSVQQFK.E
897.9789	1793.9432	1793.9280	0.0152	0	(48)	0.0013	1	R.QLIMANPQMQQLIQR.N + Gln->pyro-Glu (N-term Q)
906.4895	1810.9644	1810.9546	0.0099	0	(59)	0.0001	1	R.QLIMANPQMQQLIQR.N
914.4824	1826.9502	1826.9495	0.0008	0	(18)	1.4	1	R.QLIMANPQMQQLIQR.N + Oxidation (M)
914.4832	1826.9518	1826.9495	0.0024	0	70	7.8e-06	1	R.QLIMANPQMQQLIQR.N + Oxidation (M)
637.3199	1908.9379	1908.9516	-0.0137	0	(43)	0.0039	1	R.FQQQLEQLSAMGFLNR.E
955.4865	1908.9584	1908.9516	0.0068	0	57	0.00015	1	R.FQQQLEQLSAMGFLNR.E
665.9931	1994.9575	1994.9585	-0.0010	1	50	0.0009	1	K.EKEEFAVPENSSVQQFK.E
1020.5370	2039.0594	2039.0647	-0.0052	0	37	0.016	1	R.EANLQALIATGGDINAAIER.L
882.4273	2644.2601	2644.2737	-0.0137	0	120	6.9e-11	1	R.QQLPTFLQQMQNPDTLSAMSNPR.A

**Spot D7**	UBQL1_RAT Mass: 62032 Score: 179 Queries matched: 11 emPAI: 0.50<br>Ubiquilin-1 OS=Rattus norvegicus GN=Ubqln1 PE=1 SV=1							
Observed	Mr(expt)	Mr(calc)	Delta	Miss	Score	Expect	Rank	Peptide
619.7624	1237.5102	1237.5076	0.0027	0	44	0.0033	1	R.NPAMMQEMMR.N
635.7662	1269.5178	1269.4974	0.0205	0	(23)	0.44	1	R.NPAMMQEMMR.N + 2 Oxidation (M)
720.9168	1439.8190	1439.8136	0.0054	0	8	11	1	K.SHIDQLVLIFAGK.I
869.9215	1737.8284	1737.8210	0.0075	0	18	1.5	1	K.EEFAVPENSSVQQFK.E
897.9768	1793.9390	1793.9280	0.0110	0	62	5.2e-05	1	R.QLIMANPQMQQLIQR.N + Gln->pyro-Glu (N-term Q)
914.4857	1826.9568	1826.9495	0.0074	0	(40)	0.0077	1	R.QLIMANPQMQQLIQR.N + Oxidation (M)
637.3237	1908.9493	1908.9516	-0.0023	0	51	0.00074	1	R.FQQQLEQLSAMGFLNR.E
955.4862	1908.9578	1908.9516	0.0062	0	(50)	0.00082	1	R.FQQQLEQLSAMGFLNR.E
665.9915	1994.9527	1994.9585	-0.0058	1	44	0.0031	1	K.EKEEFAVPENSSVQQFK.E
998.4911	1994.9676	1994.9585	0.0091	1	(31)	0.068	1	K.EKEEFAVPENSSVQQFK.E
1020.5399	2039.0652	2039.0647	0.0006	0	23	0.4	1	R.EANLQALIATGGDINAAIER.L

**Spot D8**	PAK2_RAT Mass: 57924 Score: 425 Queries matched: 15 emPAI: 0.98<br>Serine/threonine-protein kinase PAK 2 OS=Rattus norvegicus GN=Pak2 PE=1 SV=1							
Observed	Mr(expt)	Mr(calc)	Delta	Miss	Score	Expect	Rank	Peptide
541.2850	1080.5554	1080.5491	0.0063	0	17	1.5	1	K.YLSFTPPEK.D
558.2800	1114.5454	1114.5329	0.0126	0	72	4.8e-06	1	R.MSSTIFSTGGK.D
562.8190	1123.6234	1123.6125	0.0110	0	9	10	1	K.IISIFSSTEK.G
562.8313	1123.6480	1123.6390	0.0091	0	13	4	1	K.ELLQHPFLK.L
612.8745	1223.7344	1223.7238	0.0107	1	68	1.1e-05	1	K.KNPQAVLDVLK.F

Table S6. List of peptide sequences for each protein assignment

**Spot D8**	PAK2_RAT Mass: 57924 Score: 425 Queries matched: 15 emPAI: 0.98<br>Serine/threonine-protein kinase PAK 2 OS=Rattus norvegicus GN=Pak2 PE=1 SV=1							
Observed	Mr(expt)	Mr(calc)	Delta	Miss	Score	Expect	Rank	Peptide
652.8352	1303.6558	1303.6408	0.0150	0	49	0.00098	1	K.DGFPSGTPALNTK.G
657.8955	1313.7764	1313.7628	0.0136	0	(18)	1.1	1	K.ELIINEILVMK.E
665.8925	1329.7704	1329.7577	0.0127	0	38	0.012	1	K.ELIINEILVMK.E + Oxidation (M)
674.8486	1347.6826	1347.6704	0.0122	0	47	0.0018	1	K.SDNVLLGMEGSVK.L
721.9443	1441.8740	1441.8578	0.0163	1	53	0.00031	1	K.KELIINEILVMK.E
546.0195	1635.0367	1635.0334	0.0032	0	(51)	0.00032	1	K.LAKPLSSLTPLILAAK.E
818.5329	1635.0512	1635.0334	0.0178	0	68	6.6e-06	1	K.LAKPLSSLTPLILAAK.E
969.5020	1936.9894	1936.9742	0.0153	0	(52)	0.00046	1	R.SVIDPIPAPVGDSNVDSGAK.S
969.5023	1936.9900	1936.9742	0.0159	0	61	5.7e-05	1	R.SVIDPIPAPVGDSNVDSGAK.S
686.7015	2057.0827	2057.0793	0.0034	0	73	3.9e-06	1	K.DPLSANHSLKPLPSVPEEK.K

**Spot D9**	CALR_RAT Mass: 47966 Score: 686 Queries matched: 43 emPAI: 1.28<br>Calreticulin OS=Rattus norvegicus GN=Calr PE=1 SV=1							
Observed	Mr(expt)	Mr(calc)	Delta	Miss	Score	Expect	Rank	Peptide
434.7159	867.4172	867.4127	0.0046	0	9	8.8	1	R.FEPFSNK.G
487.7680	973.5214	973.5233	-0.0018	0	(15)	2.1	1	K.LFPGGLDQK.D
487.7681	973.5216	973.5233	-0.0016	0	29	0.09	1	K.LFPGGLDQK.D
510.2784	1018.5422	1018.5600	-0.0177	0	(35)	0.024	1	K.VHVIFNYK.G
510.2912	1018.5678	1018.5600	0.0078	0	42	0.0049	1	K.VHVIFNYK.G
524.2403	1046.4660	1046.4628	0.0032	1	17	1.5	1	K.DKQDEEQR.L
610.3574	1218.7002	1218.6972	0.0030	0	(25)	0.22	1	K.GQTLVVQFTVK.H
610.3575	1218.7004	1218.6972	0.0032	0	(19)	0.82	1	K.GQTLVVQFTVK.H
610.3576	1218.7006	1218.6972	0.0034	0	(10)	6.8	1	K.GQTLVVQFTVK.H
610.3583	1218.7020	1218.6972	0.0048	0	(36)	0.018	1	K.GQTLVVQFTVK.H
610.3588	1218.7030	1218.6972	0.0058	0	42	0.0041	1	K.GQTLVVQFTVK.H
610.3588	1218.7030	1218.6972	0.0058	0	(21)	0.64	1	K.GQTLVVQFTVK.H
610.3596	1218.7046	1218.6972	0.0074	0	(10)	7.6	1	K.GQTLVVQFTVK.H
610.3598	1218.7050	1218.6972	0.0078	0	(34)	0.031	1	K.GQTLVVQFTVK.H
610.3599	1218.7052	1218.6972	0.0080	0	(22)	0.42	1	K.GQTLVVQFTVK.H
610.3601	1218.7056	1218.6972	0.0084	0	(25)	0.23	1	K.GQTLVVQFTVK.H
610.3604	1218.7062	1218.6972	0.0090	0	(7)	15	1	K.GQTLVVQFTVK.H
610.3606	1218.7066	1218.6972	0.0094	0	(36)	0.02	1	K.GQTLVVQFTVK.H
610.3607	1218.7068	1218.6972	0.0096	0	(36)	0.018	1	K.GQTLVVQFTVK.H
610.3607	1218.7068	1218.6972	0.0096	0	(27)	0.14	1	K.GQTLVVQFTVK.H
610.3610	1218.7074	1218.6972	0.0102	0	(21)	0.54	1	K.GQTLVVQFTVK.H
610.3610	1218.7074	1218.6972	0.0102	0	(29)	0.092	1	K.GQTLVVQFTVK.H
610.3612	1218.7078	1218.6972	0.0106	0	(33)	0.034	1	K.GQTLVVQFTVK.H
610.3612	1218.7078	1218.6972	0.0106	0	(19)	0.96	1	K.GQTLVVQFTVK.H
610.3613	1218.7080	1218.6972	0.0108	0	(16)	2	2	K.GQTLVVQFTVK.H
610.3618	1218.7090	1218.6972	0.0118	0	(37)	0.016	1	K.GQTLVVQFTVK.H
610.3619	1218.7092	1218.6972	0.0120	0	(23)	0.37	1	K.GQTLVVQFTVK.H
610.3627	1218.7108	1218.6972	0.0136	0	(36)	0.02	1	K.GQTLVVQFTVK.H
610.3635	1218.7124	1218.6972	0.0152	0	(21)	0.63	1	K.GQTLVVQFTVK.H
726.3367	1450.6588	1450.6477	0.0111	0	53	0.00042	1	K.EQFLDGDAWTNR.W

Table S6. List of peptide sequences for each protein assignment

**Spot D9**	CALR_RAT Mass: 47966 Score: 686 Queries matched: 43 emPAI: 1.28<br>Calreticulin OS=Rattus norvegicus GN=Calr PE=1 SV=1							
Observed	Mr(expt)	Mr(calc)	Delta	Miss	Score	Expect	Rank	Peptide
797.7034	2390.0884	2390.0914	-0.0030	0	41	0.0065	1	K.IDNSQVESGSLEDDWDFLPPK.K
797.7054	2390.0944	2390.0914	0.0030	0	(39)	0.011	1	K.IDNSQVESGSLEDDWDFLPPK.K
1196.0692	2390.1238	2390.0914	0.0324	0	(23)	0.43	1	K.IDNSQVESGSLEDDWDFLPPK.K
1196.0693	2390.1240	2390.0914	0.0326	0	(15)	2.8	1	K.IDNSQVESGSLEDDWDFLPPK.K
840.4013	2518.1821	2518.1864	-0.0043	1	(106)	2e-09	1	K.IDNSQVESGSLEDDWDFLPPKK.I
840.4078	2518.2016	2518.1864	0.0152	1	146	2.2e-13	1	K.IDNSQVESGSLEDDWDFLPPKK.I
690.8200	2759.2509	2759.2562	-0.0053	0	(14)	3	1	K.IDDPTDSKPEDWDKPEHIPDPDAK.K
947.4099	2839.2079	2839.2225	-0.0147	0	61	7e-05	1	K.IDDPTDSKPEDWDKPEHIPDPDAK.K + Phospho (ST)
710.8104	2839.2125	2839.2225	-0.0101	0	(18)	1.5	1	K.IDDPTDSKPEDWDKPEHIPDPDAK.K + Phospho (ST)
987.4368	2959.2886	2959.2858	0.0028	0	94	3.3e-08	1	K.KPEDWDEEMDGEWEPPVIQNPEYK.G
992.7655	2975.2747	2975.2807	-0.0060	0	(83)	4.7e-07	1	K.KPEDWDEEMDGEWEPPVIQNPEYK.G + Oxidation (M)
992.7657	2975.2753	2975.2807	-0.0054	0	(74)	3.8e-06	1	K.KPEDWDEEMDGEWEPPVIQNPEYK.G + Oxidation (M)
1090.1718	3267.4936	3267.4884	0.0052	0	11	5.9	1	K.SGTIFDNFLITNDEAYAEEFGNETWGVTK.A

**Spot D10**	GPDA_RAT Mass: 37428 Score: 348 Queries matched: 17 emPAI: 2.47<br>Glycerol-3-phosphate dehydrogenase [NAD+], cytoplasmic OS=Rattus norvegicus GN=Gpd1 PE=1 SV=4							
Observed	Mr(expt)	Mr(calc)	Delta	Miss	Score	Expect	Rank	Peptide
515.3223	1028.6300	1028.6230	0.0071	0	31	0.062	1	K.ANTIGISLIK.G
515.3225	1028.6304	1028.6230	0.0075	0	(22)	0.44	1	K.ANTIGISLIK.G
543.3098	1084.6050	1084.5957	0.0093	0	25	0.22	1	K.FPLFTAVYK.V
559.2788	1116.5430	1116.5386	0.0044	0	38	0.012	1	K.ELMQTPNFR.I + Glu->pyro-Glu (N-term E)
568.2855	1134.5564	1134.5492	0.0073	0	(33)	0.038	1	K.ELMQTPNFR.I
612.3350	1222.6554	1222.6454	0.0101	0	72	5.1e-06	1	R.LGLMEMIAFAK.L
620.3326	1238.6506	1238.6403	0.0104	0	(71)	5.3e-06	1	R.LGLMEMIAFAK.L + Oxidation (M)
628.3309	1254.6472	1254.6352	0.0121	0	(53)	0.00035	1	R.LGLMEMIAFAK.L + 2 Oxidation (M)
769.8746	1537.7346	1537.7235	0.0111	0	70	8.3e-06	1	R.VTMWVFEEDIGGR.K
769.8747	1537.7348	1537.7235	0.0113	0	(16)	2.2	1	R.VTMWVFEEDIGGR.K
777.8730	1553.7314	1553.7184	0.0130	0	(65)	2.6e-05	1	R.VTMWVFEEDIGGR.K + Oxidation (M)
533.3036	1596.8890	1596.8916	-0.0026	1	68	1.2e-05	1	K.GLVDKFPLFTAVYK.V
799.4594	1596.9042	1596.8916	0.0127	1	(58)	0.00012	1	K.GLVDKFPLFTAVYK.V
537.9457	1610.8153	1610.8165	-0.0012	0	(29)	0.11	1	K.IVGSNASQLAHFDPR.V
806.4221	1610.8296	1610.8165	0.0132	0	36	0.021	1	K.IVGSNASQLAHFDPR.V
813.4550	1624.8954	1624.8865	0.0090	1	(34)	0.029	1	K.GLVDKFPLFTAVYK.V + Formyl (K)
846.4016	1690.7886	1690.7828	0.0058	0	(13)	3.8	1	K.IVGSNASQLAHFDPR.V + Phospho (ST)

**Spot D11**	GPDA_RAT Mass: 37428 Score: 146 Queries matched: 11 emPAI: 0.78<br>Glycerol-3-phosphate dehydrogenase [NAD+], cytoplasmic OS=Rattus norvegicus GN=Gpd1 PE=1 SV=4							
Observed	Mr(expt)	Mr(calc)	Delta	Miss	Score	Expect	Rank	Peptide
515.3214	1028.6282	1028.6230	0.0053	0	(12)	5.2	1	K.ANTIGISLIK.G
515.3215	1028.6284	1028.6230	0.0055	0	24	0.28	1	K.ANTIGISLIK.G
543.3079	1084.6012	1084.5957	0.0055	0	40	0.0085	1	K.FPLFTAVYK.V
568.2864	1134.5582	1134.5492	0.0091	0	12	4.5	2	K.ELMQTPNFR.I
612.3331	1222.6516	1222.6454	0.0063	0	(1)	60	1	R.LGLMEMIAFAK.L
620.3350	1238.6554	1238.6403	0.0152	0	(36)	0.017	1	R.LGLMEMIAFAK.L + Oxidation (M)
628.3266	1254.6386	1254.6352	0.0035	0	54	0.00031	1	R.LGLMEMIAFAK.L + 2 Oxidation (M)

Table S6. List of peptide sequences for each protein assignment

Spot								
**Spot D11**	GPDA_RAT Mass: 37428 Score: 146 Queries matched: 11 emPAI: 0.78							
	Glycerol-3-phosphate dehydrogenase [NAD+], cytoplasmic OS=Rattus norvegicus GN=Gpd1 PE=1 SV=4							
Observed	Mr(expt)	Mr(calc)	Delta	Miss	Score	Expect	Rank	Peptide
777.8730	1553.7314	1553.7184	0.0130	0	70	7.7e-06	1	R.VTMWVFEEDIGGR.K + Oxidation (M)
533.3021	1596.8845	1596.8916	-0.0071	1	46	0.002	1	K.GLVDKFPLFTAVYK.V
537.9468	1610.8186	1610.8165	0.0021	0	18	1.2	1	K.IVGSNASQLAHFDPR.V
846.3979	1690.7812	1690.7828	-0.0016	0	(13)	4.1	2	K.IVGSNASQLAHFDPR.V + Phospho (ST)
**Spot D12**	ACLY_RAT Mass: 120559 Score: 139 Queries matched: 11 emPAI: 0.16							
	ATP-citrate synthase OS=Rattus norvegicus GN=Acly PE=1 SV=1							
Observed	Mr(expt)	Mr(calc)	Delta	Miss	Score	Expect	Rank	Peptide
467.7749	933.5352	933.5317	0.0035	0	44	0.0027	1	K.TILSLMTR.E
467.7755	933.5364	933.5317	0.0047	0	(15)	2.1	1	K.TILSLMTR.E
565.3064	1128.5982	1128.5928	0.0055	0	1	62	1	K.EHEVTIFVR.R
589.3297	1176.6448	1176.6390	0.0058	0	37	0.015	1	K.DGVYILDLAAK.V
692.4110	1382.8074	1382.8133	-0.0059	0	(1)	61	2	K.LGLVGVNLSLDGVK.S
692.4131	1382.8116	1382.8133	-0.0017	0	31	0.057	1	K.LGLVGVNLSLDGVK.S
709.3548	1416.6950	1416.6826	0.0124	0	45	0.0026	1	K.WGDIEFPPPFGR.E
746.3709	1490.7272	1490.7147	0.0125	0	21	0.64	1	R.SGGMSNELNNIISR.T
823.9568	1645.8990	1645.8887	0.0104	0	44	0.0028	1	K.DLVSSLTSGLLTIGDR.F
849.4059	1696.7972	1696.7831	0.0141	0	73	4.4e-06	1	R.EAYPEEAYIADLDAK.S
849.4067	1696.7988	1696.7831	0.0157	0	(10)	7.7	1	R.EAYPEEAYIADLDAK.S
**Spot D13**	IF4A3_RAT Mass: 46811 Score: 269 Queries matched: 11 emPAI: 1.33							
	Eukaryotic initiation factor 4A-III OS=Rattus norvegicus GN=Eif4a3 PE=1 SV=1							
Observed	Mr(expt)	Mr(calc)	Delta	Miss	Score	Expect	Rank	Peptide
440.2218	878.4290	878.4320	-0.0030	0	17	1.3	1	K.FMTDPIR.I
498.2801	994.5456	994.5236	0.0220	0	34	0.03	1	K.QFFVAVER.E
509.2804	1016.5462	1016.5390	0.0073	0	17	1.5	1	R.DELTLEGIK.Q
580.3268	1158.6390	1158.6397	-0.0007	0	63	3.9e-05	1	R.VLISTDVWAR.G
587.3271	1172.6396	1172.6401	-0.0004	1	57	0.00017	1	K.RDELTLEGIK.Q
606.8348	1211.6550	1211.6874	-0.0323	0	46	0.0018	1	R.ETQALILAPTR.E
760.8770	1519.7394	1519.7262	0.0133	0	(37)	0.018	1	K.MLVLDEADEMLNK.G
768.8798	1535.7450	1535.7211	0.0239	0	71	6.9e-06	1	K.MLVLDEADEMLNK.G + Oxidation (M)
609.9878	1826.9416	1826.9315	0.0101	0	(62)	4.6e-05	1	R.GIYAYGFEKPSAIQQR.A
914.4880	1826.9614	1826.9315	0.0299	0	70	7.2e-06	1	R.GIYAYGFEKPSAIQQR.A
1070.6006	2139.1866	2139.1324	0.0542	0	34	0.024	1	R.GLDVPQVSLIINYDLPNNR.E
**Spot D14**	EF1G_RAT Mass: 50029 Score: 179 Queries matched: 10 emPAI: 0.54							
	Elongation factor 1-gamma OS=Rattus norvegicus GN=Eef1g PE=2 SV=3							
Observed	Mr(expt)	Mr(calc)	Delta	Miss	Score	Expect	Rank	Peptide
488.2404	974.4662	974.5185	-0.0523	0	41	0.0054	1	K.QVLEPSFR.Q
543.2953	1084.5760	1084.5441	0.0320	0	19	1	1	K.STFVLDEFK.R
561.8607	1121.7068	1121.6808	0.0260	0	(8)	11	1	R.ILGLLDTHLK.T
561.8648	1121.7150	1121.6808	0.0342	0	36	0.018	1	R.ILGLLDTHLK.T
621.3463	1240.6780	1240.6452	0.0329	1	54	0.00034	1	K.STFVLDEFKR.K
681.3628	1360.7110	1360.7463	-0.0352	0	76	1.8e-06	1	K.ALIAAQYSGAQIR.V
776.9103	1551.8060	1551.7657	0.0404	0	25	0.23	1	R.WFLTCINQPQFR.A
786.9190	1571.8234	1571.8155	0.0080	1	84	3.1e-07	1	R.KLDPGSEETQTLVR.E

Table S6. List of peptide sequences for each protein assignment

**Spot D14**	EF1G_RAT Mass: 50029 Score: 179 Queries matched: 10 emPAI: 0.54<br>Elongation factor 1-gamma OS=Rattus norvegicus GN=Eef1g PE=2 SV=3							
Observed	Mr(expt)	Mr(calc)	Delta	Miss	Score	Expect	Rank	Peptide
854.4546	1706.8946	1706.8641	0.0305	0	0	75	1	R.VLSAPPHFHFGQTNR.T
891.7697	2672.2873	2672.2183	0.0689	0	20	0.71	1	R.GQDLAFPLSPDWQVDYESYTWR.K

**Spot D15**	PRS4_RAT Mass: 49154 Score: 256 Queries matched: 15 emPAI: 1.08<br>26S protease regulatory subunit 4 OS=Rattus norvegicus GN=Psmc1 PE=2 SV=1							
Observed	Mr(expt)	Mr(calc)	Delta	Miss	Score	Expect	Rank	Peptide
467.2477	932.4808	932.4451	0.0358	0	17	1.4	1	K.DDLSGADIK.A
544.3015	1086.5884	1086.5597	0.0287	0	26	0.19	1	K.IEFPLPDEK.T
579.8340	1157.6534	1157.6445	0.0090	0	43	0.0035	1	K.GVILYGPPGTGK.T
608.3623	1214.7100	1214.6547	0.0554	1	53	0.0004	1	R.KIEFPLPDEK.T
617.8372	1233.6598	1233.6241	0.0357	1	25	0.26	1	K.KQEGTPEGLYL.-
639.8372	1277.6598	1277.6728	-0.0129	0	66	2.2e-05	1	K.AVANQTSATFLR.V
687.3613	1372.7080	1372.6656	0.0424	0	28	0.14	1	R.NQEQMKPLEEK.Q
725.9450	1449.8754	1449.8303	0.0451	0	87	1.4e-07	1	R.IETLDPALIRPGR.I
729.3797	1456.7448	1456.6908	0.0541	0	4	30	1	K.DYLLMEEEFIR.N
876.4627	1750.9108	1750.8560	0.0549	0	(23)	0.4	1	R.TMLELLNQLDGFDSR.G
876.4669	1750.9192	1750.8560	0.0633	0	(11)	6	1	R.TMLELLNQLDGFDSR.G
882.9713	1763.9280	1763.8685	0.0595	0	59	9.4e-05	1	R.MTLADDVTLDDLIMAK.D
884.4583	1766.9020	1766.8509	0.0512	0	75	2.7e-06	1	R.TMLELLNQLDGFDSR.G + Oxidation (M)
890.9646	1779.9146	1779.8634	0.0512	0	(10)	9.1	1	R.MTLADDVTLDDLIMAK.D + Oxidation (M)
1102.0580	2202.1014	2202.0804	0.0210	0	29	0.11	1	K.APQETYADIGGLDNQIQEIK.E

**Spot D16**	ATPB_RAT Mass: 56318 Score: 69 Queries matched: 2 emPAI: 0.07<br>ATP synthase subunit beta, mitochondrial OS=Rattus norvegicus GN=Atp5b PE=1 SV=2							
Observed	Mr(expt)	Mr(calc)	Delta	Miss	Score	Expect	Rank	Peptide
720.4167	1438.8188	1438.7820	0.0368	0	69	9e-06	1	R.VALTGLTVAEYFR.D
994.5417	1987.0688	1987.0262	0.0426	0	0	71	4	R.AIAELGIYPAVDPLDSTSR.I

**Spot D17**	ATPB_RAT Mass: 56318 Score: 323 Queries matched: 11 emPAI: 0.67<br>ATP synthase subunit beta, mitochondrial OS=Rattus norvegicus GN=Atp5b PE=1 SV=2							
Observed	Mr(expt)	Mr(calc)	Delta	Miss	Score	Expect	Rank	Peptide
519.8017	1037.5888	1037.5869	0.0019	0	33	0.036	1	K.IPVGPETLGR.I
639.8073	1277.6000	1277.6286	-0.0285	0	84	3.1e-07	1	R.TIAMDGTEGLVR.G + Oxidation (M)
693.3598	1384.7050	1384.7020	0.0030	0	(19)	0.92	1	R.IMNVIGEPIDER.G
701.3397	1400.6648	1400.6969	-0.0321	0	45	0.0026	1	R.IMNVIGEPIDER.G + Oxidation (M)
718.3795	1434.7444	1434.7467	-0.0022	0	84	2.8e-07	1	R.FTQAGSEVSALLGR.I
720.3864	1438.7582	1438.7820	-0.0238	0	74	3.1e-06	1	R.VALTGLTVAEYFR.D
729.4247	1456.8348	1456.8323	0.0025	0	(5)	22	1	K.TVLIMELINNVAK.A
737.4190	1472.8234	1472.8272	-0.0038	0	65	2.5e-05	1	K.TVLIMELINNVAK.A + Oxidation (M)
961.5010	1920.9874	1920.9581	0.0293	0	65	2.5e-05	1	R.DQEGQDVLLFIDNIFR.F
994.5090	1987.0034	1987.0262	-0.0228	0	49	0.0011	1	R.AIAELGIYPAVDPLDSTSR.I
897.7979	2690.3719	2690.3684	0.0035	1	17	1.4	1	K.SLQDIIAILGMDELSEEDKLTVSR.A + Oxidation (M)

**Spot D18**	ACTZ_RAT Mass: 42587 Score: 225 Queries matched: 16 emPAI: 0.66<br>Alpha-centractin OS=Rattus norvegicus GN=Actr1a PE=1 SV=1							
Observed	Mr(expt)	Mr(calc)	Delta	Miss	Score	Expect	Rank	Peptide
502.2629	1002.5112	1002.5134	-0.0022	0	20	0.81	1	K.AGFAGDQIPK.Y

Table S6. List of peptide sequences for each protein assignment

**Spot D18**	ACTZ_RAT Mass: 42587 Score: 225 Queries matched: 16 emPAI: 0.66<br>Alpha-centractin OS=Rattus norvegicus GN=Actr1a PE=1 SV=1							
Observed	Mr(expt)	Mr(calc)	Delta	Miss	Score	Expect	Rank	Peptide
537.2841	1072.5536	1072.5375	0.0161	0	29	0.1	1	R.YPMEHGIVK.D
543.7833	1085.5520	1085.5546	-0.0025	0	36	0.019	1	R.IWQYVYSK.D
759.4241	1516.8336	1516.7959	0.0377	0	48	0.0012	1	R.VMAGALEGDIFIGPK.A
759.4297	1516.8448	1516.7959	0.0489	0	(26)	0.19	1	R.VMAGALEGDIFIGPK.A
759.4316	1516.8486	1516.7959	0.0527	0	(5)	22	1	R.VMAGALEGDIFIGPK.A
759.4337	1516.8528	1516.7959	0.0569	0	(10)	7.9	1	R.VMAGALEGDIFIGPK.A
561.9974	1682.9704	1682.9243	0.0460	0	(22)	0.48	1	R.TLFSNIVLSGGSTLFK.G
842.4980	1682.9814	1682.9243	0.0571	0	28	0.1	1	R.TLFSNIVLSGGSTLFK.G
842.5039	1682.9932	1682.9243	0.0689	0	(8)	11	1	R.TLFSNIVLSGGSTLFK.G
882.4800	1762.9454	1762.8907	0.0548	0	(19)	0.99	1	R.TLFSNIVLSGGSTLFK.G + Phospho (ST)
882.4863	1762.9580	1762.8907	0.0674	0	(5)	24	1	R.TLFSNIVLSGGSTLFK.G + Phospho (ST)
896.3930	1790.7714	1790.8856	-0.1141	0	(0)	89	2	R.TLFSNIVLSGGSTLFK.G + Phospho (ST); Formyl (K)
933.9761	1865.9376	1865.9159	0.0217	0	82	5.4e-07	1	K.AQYYLPDGSTIEIGPSR.F
986.5652	1971.1158	1971.0353	0.0805	0	29	0.086	1	R.LYSTWIGGSILASLDTFK.K
845.4417	2533.3033	2533.2812	0.0220	0	108	1.2e-09	1	K.DQLQTFSEEHPVLLTEAPLNPR.K

**Spot D19**	ACTZ_RAT Mass: 42587 Score: 239 Queries matched: 9 emPAI: 0.52<br>Alpha-centractin OS=Rattus norvegicus GN=Actr1a PE=1 SV=1							
Observed	Mr(expt)	Mr(calc)	Delta	Miss	Score	Expect	Rank	Peptide
543.8019	1085.5892	1085.5546	0.0347	0	14	2.7	1	R.IWQYVYSK.D
759.4266	1516.8386	1516.7959	0.0427	0	(70)	8.5e-06	1	R.VMAGALEGDIFIGPK.A
767.4064	1532.7982	1532.7909	0.0074	0	92	4.8e-08	1	R.VMAGALEGDIFIGPK.A + Oxidation (M)
842.5032	1682.9918	1682.9243	0.0675	0	70	6e-06	1	R.TLFSNIVLSGGSTLFK.G
882.4739	1762.9332	1762.8907	0.0426	0	(1)	61	1	R.TLFSNIVLSGGSTLFK.G + Phospho (ST)
887.4363	1772.8580	1772.7893	0.0687	0	12	5.8	1	K.EGYDFHSSSEFEIVK.A
927.4218	1852.8290	1852.7557	0.0734	0	(6)	25	1	K.EGYDFHSSSEFEIVK.A + Phospho (ST)
933.9854	1865.9562	1865.9159	0.0403	0	53	0.0004	1	K.AQYYLPDGSTIEIGPSR.F
845.4539	2533.3399	2533.2812	0.0586	0	50	0.00064	1	K.DQLQTFSEEHPVLLTEAPLNPR.K

**Spot D20**	HS90B_RAT Mass: 83229 Score: 410 Queries matched: 20 emPAI: 0.76<br>Heat shock protein HSP 90-beta OS=Rattus norvegicus GN=Hsp90ab1 PE=1 SV=4							
Observed	Mr(expt)	Mr(calc)	Delta	Miss	Score	Expect	Rank	Peptide
451.2648	900.5150	900.5181	-0.0031	0	7	38	1	K.TKPIWTR.N
476.2342	950.4538	950.4570	-0.0031	0	29	0.22	1	R.ADHGEPIGR.G
520.2490	1038.4834	1038.4869	-0.0035	0	50	0.002	1	R.YESLTDPSK.L
523.2847	1044.5548	1044.5927	-0.0379	1	16	4.8	2	K.VEKVTISNR.L
576.2868	1150.5590	1150.5506	0.0085	0	42	0.013	1	K.YIDQEELNK.T
597.8220	1193.6294	1193.6404	-0.0110	0	65	6.4e-05	1	K.IDIIPNPQER.T
621.8548	1241.6950	1241.6979	-0.0029	0	58	0.00035	1	K.ADLINNLGTIAK.S
622.3501	1242.6856	1242.6819	0.0037	0	(32)	0.14	1	K.ADLINNLGTIAK.S + Deamidated (NQ)
638.3279	1274.6412	1274.6354	0.0059	0	14	9.4	1	R.ELISNASDALDK.I
654.8362	1307.6578	1307.6721	-0.0143	1	7	51	8	K.IRYESLTDPSK.L
656.2899	1310.5652	1310.5626	0.0026	0	67	4.9e-05	1	K.EDQTEYLEER.R
675.3687	1348.7228	1348.7272	-0.0044	0	(73)	1.2e-05	1	R.TLTLVDTGIGMTK.A
683.3688	1364.7230	1364.7221	0.0009	0	81	1.9e-06	1	R.TLTLVDTGIGMTK.A + Oxidation (M)

Table S6. List of peptide sequences for each protein assignment

**Spot D20**	HS90B_RAT Mass: 83229 Score: 410 Queries matched: 20 emPAI: 0.76<br>Heat shock protein HSP 90-beta OS=Rattus norvegicus GN=Hsp90ab1 PE=1 SV=4							
Observed	Mr(expt)	Mr(calc)	Delta	Miss	Score	Expect	Rank	Peptide
747.8060	1493.5974	1493.6005	-0.0031	0	86	7e-07	1	K.IEDVGSDEEDDSGK.D
509.9153	1526.7241	1526.7365	-0.0124	0	(12)	20	1	K.SLTNDWEDHLAVK.H
764.3754	1526.7362	1526.7365	-0.0003	0	68	4.6e-05	1	K.SLTNDWEDHLAVK.H
770.3828	1538.7510	1538.7464	0.0046	1	6	80	1	R.YESLTDPSKLDSGK.E
579.9102	1736.7088	1736.7224	-0.0137	1	52	0.0024	1	K.IEDVGSDEEDDSGKDK.K
909.3420	1816.6694	1816.6888	-0.0193	1	(2)	2.1e+02	1	K.IEDVGSDEEDDSGKDK.K + Phospho (ST)
924.3999	1846.7852	1846.7897	-0.0045	0	59	0.0005	1	R.NPDDITQEEYGEFYK.S

**Spot D21**	HSP7C_RAT Mass: 70827 Score: 299 Queries matched: 14 emPAI: 0.75<br>Heat shock cognate 71 kDa protein OS=Rattus norvegicus GN=Hspa8 PE=1 SV=1							
Observed	Mr(expt)	Mr(calc)	Delta	Miss	Score	Expect	Rank	Peptide
429.7221	857.4296	857.4494	-0.0198	0	15	5.5	1	R.GTLDPVEK.A
472.7563	943.4980	943.5161	-0.0180	0	11	17	1	K.VCNPIITK.L + Carbamidomethyl (C)
495.2573	988.5000	988.5189	-0.0188	1	49	0.0027	1	R.LSKEDIER.M
509.7711	1017.5276	1017.5455	-0.0178	1	29	0.23	1	K.ITITNDKGR.L + Deamidated (NQ)
541.7734	1081.5322	1081.5444	-0.0122	0	47	0.004	1	K.LLQDFFNGK.E + Deamidated (NQ)
599.3215	1196.6284	1196.6553	-0.0269	0	44	0.0085	1	K.FELTGIPPAPR.G
627.2993	1252.5840	1252.6088	-0.0247	0	74	9.2e-06	1	R.FEELNADLFR.G
639.3124	1276.6102	1276.6122	-0.0019	0	33	0.12	1	K.CNEIISWLDK.N + Carbamidomethyl (C)
660.2874	1318.5602	1318.5863	-0.0261	0	58	0.00045	1	K.NSLESYAFNMK.A + Oxidation (M)
660.7899	1319.5652	1319.5703	-0.0051	0	(24)	1.1	1	K.NSLESYAFNMK.A + Deamidated (NQ); Oxidation (M)
741.3934	1480.7722	1480.7998	-0.0276	0	54	0.0011	1	K.SQIHDIVLVGGSTR.I
754.0332	2259.0778	2259.1383	-0.0605	0	63	0.00024	1	K.SINPDEAVAYGAAVQAAILSGDK.S
1130.5558	2259.0970	2259.1383	-0.0412	0	(49)	0.0052	1	K.SINPDEAVAYGAAVQAAILSGDK.S
925.4310	2773.2712	2773.3195	-0.0483	0	32	0.37	1	K.QTQTFTTYSDNQPGVLIQVYEGER.A

**Spot D22**	ACTA_RAT Mass: 41982 Score: 64 Queries matched: 3 emPAI: 0.29<br>Actin, aortic smooth muscle OS=Rattus norvegicus GN=Acta2 PE=1 SV=1							
Observed	Mr(expt)	Mr(calc)	Delta	Miss	Score	Expect	Rank	Peptide
581.3173	1160.6200	1160.6111	0.0090	0	23	0.39	1	K.EITALAPSTMK.I
654.3089	1959.9049	1959.9036	0.0012	0	50	0.00093	1	K.YPIEHGIITNWDDMEK.I
850.7284	2549.1634	2549.1665	-0.0031	0	33	0.047	1	K.LCYVALDFENEMATAASSSSLEK.S + Propionamide (C)

**Spot D23**	ACTG_RAT Mass: 41766 Score: 186 Queries matched: 10 emPAI: 0.54<br>Actin, cytoplasmic 2 OS=Rattus norvegicus GN=Actg1 PE=1 SV=1							
Observed	Mr(expt)	Mr(calc)	Delta	Miss	Score	Expect	Rank	Peptide
532.8231	1063.6316	1063.6389	-0.0073	1	23	0.35	1	K.IKIIAPPER.K + Formyl (K)
566.7733	1131.5320	1131.5197	0.0124	0	7	16	4	R.GYSFTTTAER.E
581.3168	1160.6190	1160.6111	0.0080	0	32	0.046	1	K.EITALAPSTMK.I
589.3164	1176.6182	1176.6060	0.0122	0	(4)	27	1	K.EITALAPSTMK.I + Oxidation (M)
691.8201	1381.6256	1381.6110	0.0147	1	4	32	1	K.DSYVGDEAQSKR.G + Formyl (K)
895.9558	1789.8970	1789.8846	0.0124	0	78	1.3e-06	1	K.SYELPDGQVITIGNER.F
911.9058	1821.7970	1821.8124	-0.0154	0	8	16	1	M.EEEIAALVIDNGSGMCK.A + Oxidation (M); Formyl (K)
652.0268	1953.0586	1953.0571	0.0015	0	81	6.1e-07	1	R.VAPEEHPVLLTEAPLNPK.A
1108.0480	2214.0814	2214.0627	0.0188	0	53	0.00041	1	K.DLYANTVLSGGTTMYPGIADR.M
775.0838	2322.2296	2322.2332	-0.0036	1	2	39	1	R.VAPEEHPVLLTEAPLNPKANR.E + Formyl (K)

Table S6. List of peptide sequences for each protein assignment

**Spot D24**	ENOA_RAT Mass: 47098 Score: 50 Queries matched: 1 emPAI: 0.08<br>Alpha-enolase OS=Rattus norvegicus GN=Eno1 PE=1 SV=4							
Observed	Mr(expt)	Mr(calc)	Delta	Miss	Score	Expect	Rank	Peptide
703.8680	1405.7214	1405.7089	0.0125	0	50	0.00073	1	R.GNPTVEVDLYTAK.G

**Spot D25**	GPDA_RAT Mass: 37428 Score: 351 Queries matched: 16 emPAI: 1.60<br>Glycerol-3-phosphate dehydrogenase [NAD+], cytoplasmic OS=Rattus norvegicus GN=Gpd1 PE=1 SV=4							
Observed	Mr(expt)	Mr(calc)	Delta	Miss	Score	Expect	Rank	Peptide
543.3069	1084.5992	1084.5957	0.0035	0	(1)	61	5	K.FPLFTAVYK.V
543.3081	1084.6016	1084.5957	0.0059	0	32	0.051	1	K.FPLFTAVYK.V
568.2850	1134.5554	1134.5492	0.0063	0	(16)	1.9	1	K.ELMQTPNFR.I
576.2833	1150.5520	1150.5441	0.0080	0	29	0.11	1	K.ELMQTPNFR.I + Oxidation (M)
612.3346	1222.6546	1222.6454	0.0093	0	59	0.00011	1	R.LGLMEMIAFAK.L
620.3321	1238.6496	1238.6403	0.0094	0	(13)	3.7	1	R.LGLMEMIAFAK.L + Oxidation (M)
769.8716	1537.7286	1537.7235	0.0051	0	(7)	15	1	R.VTMWVFEEDIGGR.K
769.8745	1537.7344	1537.7235	0.0109	0	(70)	8.5e-06	1	R.VTMWVFEEDIGGR.K
513.6091	1537.8055	1537.8100	-0.0045	0	(20)	0.74	1	K.LTEIINTQHENVK.Y
769.9164	1537.8182	1537.8100	0.0083	0	50	0.00088	1	K.LTEIINTQHENVK.Y
777.8721	1553.7296	1553.7184	0.0112	0	86	1.9e-07	1	R.VTMWVFEEDIGGR.K + Oxidation (M)
533.3050	1596.8932	1596.8916	0.0016	1	87	1.6e-07	1	K.GLVDKFPLFTAVYK.V
799.4572	1596.8998	1596.8916	0.0083	1	(50)	0.0008	1	K.GLVDKFPLFTAVYK.V
537.9448	1610.8126	1610.8165	-0.0039	0	53	0.00038	1	K.IVGSNASQLAHFDPR.V
806.4210	1610.8274	1610.8165	0.0109	0	(51)	0.0007	1	K.IVGSNASQLAHFDPR.V
846.4007	1690.7868	1690.7828	0.0040	0	(17)	1.5	1	K.IVGSNASQLAHFDPR.V + Phospho (ST)

**Spot D26**	NDRG1_RAT Mass: 42927 Score: 206 Queries matched: 6 emPAI: 0.40<br>Protein NDRG1 OS=Rattus norvegicus GN=Ndrg1 PE=1 SV=1							
Observed	Mr(expt)	Mr(calc)	Delta	Miss	Score	Expect	Rank	Peptide
791.9216	1581.8286	1581.8185	0.0102	0	52	0.00054	1	K.SVIGMGTGAGAYILTR.F + Oxidation (M)
791.9219	1581.8292	1581.8185	0.0108	0	(11)	6.1	1	K.SVIGMGTGAGAYILTR.F + Oxidation (M)
612.0033	1832.9881	1832.9884	-0.0003	0	92	4.4e-08	1	R.ELHDVDLAEVKPLVEK.G
921.4101	1840.8056	1840.7946	0.0110	0	68	1.4e-05	1	K.YFVQGMGYMPSASMTR.L + Oxidation (M)
696.0305	2085.0697	2085.0717	-0.0021	0	69	9.2e-06	1	K.ISGWTQALPDMVVSHLFGK.E
701.3600	2101.0582	2101.0667	-0.0085	0	(8)	12	1	K.ISGWTQALPDMVVSHLFGK.E + Oxidation (M)

**Spot D27**	HNRDL_RAT Mass: 35272 Score: 103 Queries matched: 2 emPAI: 0.22<br>Heterogeneous nuclear ribonucleoprotein D-like OS=Rattus norvegicus GN=Hnrpdl PE=2 SV=1							
Observed	Mr(expt)	Mr(calc)	Delta	Miss	Score	Expect	Rank	Peptide
498.7560	995.4974	995.4924	0.0051	0	34	0.025	1	K.DLTEYLSR.F
853.4418	1704.8690	1704.8570	0.0120	0	94	3.7e-08	1	K.VFVGGLSPDTSEEQIK.E

**Spot D28**	NPM_RAT Mass: 32540 Score: 418 Queries matched: 12 emPAI: 1.69<br>Nucleophosmin OS=Rattus norvegicus GN=Npm1 PE=1 SV=1							
Observed	Mr(expt)	Mr(calc)	Delta	Miss	Score	Expect	Rank	Peptide
523.5781	1567.7125	1567.7226	-0.0102	0	(55)	0.00028	1	K.VDNDENEHQLSLR.T
523.5785	1567.7137	1567.7226	-0.0090	0	(54)	0.00034	1	K.VDNDENEHQLSLR.T
784.8753	1567.7360	1567.7226	0.0134	0	132	4.9e-12	1	K.VDNDENEHQLSLR.T
910.4302	1818.8458	1818.8359	0.0099	0	(75)	3e-06	1	R.MTDQEAIQDLWQWR.K
918.4271	1834.8396	1834.8308	0.0088	0	78	1.4e-06	1	R.MTDQEAIQDLWQWR.K + Oxidation (M)
715.6773	2144.0101	2144.0095	0.0005	0	72	5.3e-06	1	K.DELHIVEAEAMNYEGSPIK.V

Table S6. List of peptide sequences for each protein assignment

**Spot D28**	NPM_RAT Mass: 32540 Score: 418 Queries matched: 12 emPAI: 1.69<br>Nucleophosmin OS=Rattus norvegicus GN=Npm1 PE=1 SV=1							
Observed	Mr(expt)	Mr(calc)	Delta	Miss	Score	Expect	Rank	Peptide
743.0763	2226.2071	2226.2083	-0.0012	0	(36)	0.015	1	K.MSVQPTVSLGGFEITPPVVLR.L
1114.1170	2226.2194	2226.2083	0.0112	0	(28)	0.1	1	K.MSVQPTVSLGGFEITPPVVLR.L
748.4097	2242.2073	2242.2032	0.0041	0	(49)	0.00081	1	K.MSVQPTVSLGGFEITPPVVLR.L + Oxidation (M)
1122.1162	2242.2178	2242.2032	0.0147	0	53	0.00034	1	K.MSVQPTVSLGGFEITPPVVLR.L + Oxidation (M)
1154.0999	2306.1852	2306.1746	0.0107	0	(29)	0.087	1	K.MSVQPTVSLGGFEITPPVVLR.L + Phospho (ST)
1162.0955	2322.1764	2322.1695	0.0070	0	(21)	0.54	1	K.MSVQPTVSLGGFEITPPVVLR.L + Oxidation (M); Phospho (ST)

**Spot D29**	NPM_RAT Mass: 32540 Score: 547 Queries matched: 20 emPAI: 2.73<br>Nucleophosmin OS=Rattus norvegicus GN=Npm1 PE=1 SV=1							
Observed	Mr(expt)	Mr(calc)	Delta	Miss	Score	Expect	Rank	Peptide
523.5796	1567.7170	1567.7226	-0.0057	0	(37)	0.015	1	K.VDNDENEHQLSLR.T
523.5796	1567.7170	1567.7226	-0.0057	0	(50)	0.00074	1	K.VDNDENEHQLSLR.T
784.8731	1567.7316	1567.7226	0.0090	0	127	1.8e-11	1	K.VDNDENEHQLSLR.T
910.4280	1818.8414	1818.8359	0.0055	0	(40)	0.0083	1	R.MTDQEAIQDLWQWR.K
918.4291	1834.8436	1834.8308	0.0128	0	77	1.6e-06	1	R.MTDQEAIQDLWQWR.K + Oxidation (M)
918.4315	1834.8484	1834.8308	0.0176	0	(3)	40	2	R.MTDQEAIQDLWQWR.K + Oxidation (M)
715.6764	2144.0074	2144.0095	-0.0022	0	(49)	0.001	1	K.DELHIVEAEAMNYEGSPIK.V
721.0092	2160.0058	2160.0045	0.0013	0	98	1.2e-08	1	K.DELHIVEAEAMNYEGSPIK.V + Oxidation (M)
1081.0172	2160.0198	2160.0045	0.0154	0	(57)	0.00018	1	K.DELHIVEAEAMNYEGSPIK.V + Oxidation (M)
1114.1202	2226.2258	2226.2083	0.0176	0	(40)	0.0062	1	K.MSVQPTVSLGGFEITPPVVLR.L
748.4059	2242.1959	2242.2032	-0.0073	0	(33)	0.035	1	K.MSVQPTVSLGGFEITPPVVLR.L + Oxidation (M)
748.4085	2242.2037	2242.2032	0.0005	0	72	3.8e-06	1	K.MSVQPTVSLGGFEITPPVVLR.L + Oxidation (M)
1122.1160	2242.2174	2242.2032	0.0143	0	(18)	0.93	1	K.MSVQPTVSLGGFEITPPVVLR.L + Oxidation (M)
1122.1170	2242.2194	2242.2032	0.0163	0	(3)	34	1	K.MSVQPTVSLGGFEITPPVVLR.L + Oxidation (M)
1122.1171	2242.2196	2242.2032	0.0165	0	(53)	0.00032	1	K.MSVQPTVSLGGFEITPPVVLR.L + Oxidation (M)
1154.1030	2306.1914	2306.1746	0.0169	0	(29)	0.088	1	K.MSVQPTVSLGGFEITPPVVLR.L + Phospho (ST)
1162.0953	2322.1760	2322.1695	0.0066	0	(11)	5.9	1	K.MSVQPTVSLGGFEITPPVVLR.L + Oxidation (M); Phospho (ST)
1162.0977	2322.1808	2322.1695	0.0114	0	(43)	0.0034	1	K.MSVQPTVSLGGFEITPPVVLR.L + Oxidation (M); Phospho (ST)
737.1152	2944.4317	2944.4488	-0.0171	1	(7)	15	1	R.TVSLGAGAKDELHIVEAEAMNYEGSPIK.V + Oxidation (M)
982.4880	2944.4422	2944.4488	-0.0066	1	100	6.7e-09	1	R.TVSLGAGAKDELHIVEAEAMNYEGSPIK.V + Oxidation (M)

**Spot D30**	HNRDL_RAT Mass: 35272 Score: 165 Queries matched: 5 emPAI: 0.22<br>Heterogeneous nuclear ribonucleoprotein D-like OS=Rattus norvegicus GN=Hnrpdl PE=2 SV=1							
Observed	Mr(expt)	Mr(calc)	Delta	Miss	Score	Expect	Rank	Peptide
457.7621	913.5096	913.5062	0.0035	0	7	15	1	R.GFGFVLFK.D
498.7549	995.4952	995.4924	0.0029	0	33	0.033	1	K.DLTEYLSR.F
853.4418	1704.8690	1704.8570	0.0120	0	89	9.9e-08	1	K.VFVGGLSPDTSEEQIK.E
853.4420	1704.8694	1704.8570	0.0124	0	(89)	1e-07	1	K.VFVGGLSPDTSEEQIK.E
1110.0238	2218.0330	2218.0140	0.0191	0	10	9.6	1	K.EYFGAFGEIENIELPMDTK.T + Oxidation (M)

**Spot D31**	EIF3I_RAT Mass: 36438 Score: 640 Queries matched: 26 emPAI: 3.82<br>Eukaryotic translation initiation factor 3 subunit I OS=Rattus norvegicus GN=Eif3i PE=2 SV=1							
Observed	Mr(expt)	Mr(calc)	Delta	Miss	Score	Expect	Rank	Peptide
472.7756	943.5366	943.5338	0.0028	0	10	8.4	1	K.SGEVLVNVK.E
535.2877	1068.5608	1068.5564	0.0045	0	(40)	0.007	1	R.QINDIQLSR.D + Gln->pyro-Glu (N-term Q)
543.8001	1085.5856	1085.5829	0.0027	0	61	6e-05	1	R.QINDIQLSR.D

Table S6. List of peptide sequences for each protein assignment

Spot D31	EIF3I_RAT Mass: 36438 Score: 640 Queries matched: 26 emPAI: 3.82<br>Eukaryotic translation initiation factor 3 subunit I OS=Rattus norvegicus GN=Eif3i PE=2 SV=1							
Observed	Mr(expt)	Mr(calc)	Delta	Miss	Score	Expect	Rank	Peptide
546.8034	1091.5922	1091.5863	0.0060	0	40	0.008	1	R.EGDLLFTVAK.D
565.7684	1129.5222	1129.5148	0.0075	0	(39)	0.011	1	R.DMTMFVTASK.D
565.7684	1129.5222	1129.5148	0.0075	0	(39)	0.011	1	R.DMTMFVTASK.D
573.7650	1145.5154	1145.5097	0.0058	0	59	9.7e-05	1	R.DMTMFVTASK.D + Oxidation (M)
573.7654	1145.5162	1145.5097	0.0066	0	(53)	0.00041	1	R.DMTMFVTASK.D + Oxidation (M)
638.7782	1275.5418	1275.5368	0.0051	0	28	0.14	1	K.SYSSGGEDGYVR.I
652.8308	1303.6470	1303.6408	0.0062	0	(21)	0.64	1	K.LFDSTSLEHQK.T
441.2492	1320.7258	1320.7336	-0.0078	0	(51)	0.00057	1	-.MKPILLQGHER.S
441.2492	1320.7258	1320.7336	-0.0078	0	52	0.00051	1	-.MKPILLQGHER.S
661.3771	1320.7396	1320.7336	0.0061	0	(21)	0.57	1	-.MKPILLQGHER.S
446.5811	1336.7215	1336.7285	-0.0070	0	(36)	0.019	1	-.MKPILLQGHER.S + Oxidation (M)
692.8101	1383.6056	1383.6071	-0.0015	0	40	0.0092	1	K.LFDSTSLEHQK.T + Phospho (ST)
762.3433	1522.6720	1522.6609	0.0111	0	86	1.9e-07	1	R.DPSQIDSNEPYMK.I
770.3385	1538.6624	1538.6559	0.0066	0	(27)	0.18	1	R.DPSQIDSNEPYMK.I + Oxidation (M)
824.4141	1646.8136	1646.8053	0.0084	0	(13)	4.1	1	K.DPIVNVWYSVNGER.L
824.4161	1646.8176	1646.8053	0.0124	0	42	0.0047	1	K.DPIVNVWYSVNGER.L
560.6119	1678.8139	1678.8216	-0.0077	0	102	5.6e-09	1	K.GHFGPINSVAFHPDGK.S
560.6139	1678.8199	1678.8216	-0.0017	0	(98)	1.5e-08	1	K.GHFGPINSVAFHPDGK.S
840.4218	1678.8290	1678.8216	0.0074	0	(49)	0.0011	1	K.GHFGPINSVAFHPDGK.S
991.4407	1980.8668	1980.8570	0.0098	0	(15)	2.8	1	R.IHYFDPQYFEFEFEA.-
991.4437	1980.8728	1980.8570	0.0158	0	(36)	0.024	1	R.IHYFDPQYFEFEFEA.-
991.4437	1980.8728	1980.8570	0.0158	0	39	0.012	1	R.IHYFDPQYFEFEFEA.-
898.1817	3588.6977	3588.7115	-0.0138	0	111	5.7e-10	1	R.TERPVNSAALSPNYDHVVLGGGQEAMDVTTTSTR.I + Oxidation ([illegible]

Spot D32	TBA1B_RAT Mass: 50120 Score: 414 Queries matched: 25 emPAI: 1.20<br>Tubulin alpha-1B chain OS=Rattus norvegicus GN=Tuba1b PE=1 SV=1							
Observed	Mr(expt)	Mr(calc)	Delta	Miss	Score	Expect	Rank	Peptide
455.2448	908.4750	908.4967	-0.0217	1	19	2.1	1	R.LSVDYGKK.S
495.2270	988.4394	988.4630	-0.0236	1	(2)	1.2e+02	1	R.LSVDYGKK.S + Phospho (ST)
534.2968	1066.5790	1066.6022	-0.0232	0	17	3.6	1	K.EIIDLVLDR.I + Glu->pyro-Glu (N-term E)
697.3624	1392.7102	1392.7402	-0.0299	0	43	0.012	1	R.QLFHPEQLITGK.E + Gln->pyro-Glu (N-term Q)
705.8772	1409.7398	1409.7667	-0.0268	0	(36)	0.06	1	R.QLFHPEQLITGK.E
705.8778	1409.7410	1409.7667	-0.0256	0	(17)	4.7	1	R.QLFHPEQLITGK.E
744.4245	1486.8344	1486.8719	-0.0374	0	(7)	60	1	R.LISQIVSSITASLR.F
744.4275	1486.8404	1486.8719	-0.0314	0	94	9.8e-08	1	R.LISQIVSSITASLR.F
744.4298	1486.8450	1486.8719	-0.0268	0	(57)	0.00048	1	R.LISQIVSSITASLR.F
784.4005	1566.7864	1566.8382	-0.0517	0	(16)	7.7	1	R.LISQIVSSITASLR.F + Phospho (ST)
784.4185	1566.8224	1566.8382	-0.0157	0	(30)	0.29	1	R.LISQIVSSITASLR.F + Phospho (ST)
851.4376	1700.8606	1700.8985	-0.0379	0	(5)	1.2e+02	1	R.AVFVDLEPTVIDEVR.T
851.4390	1700.8634	1700.8985	-0.0351	0	(20)	3.3	1	R.AVFVDLEPTVIDEVR.T
851.4402	1700.8658	1700.8985	-0.0327	0	105	1e-08	1	R.AVFVDLEPTVIDEVR.T
851.4436	1700.8726	1700.8985	-0.0259	0	(4)	1.3e+02	1	R.AVFVDLEPTVIDEVR.T
573.6159	1717.8259	1717.8747	-0.0488	0	63	0.00018	1	R.NLDIERPTYTNLNR.L
859.9276	1717.8406	1717.8747	-0.0341	0	(42)	0.021	1	R.NLDIERPTYTNLNR.L

Table S6. List of peptide sequences for each protein assignment

**Spot D32**	TBA1B_RAT Mass: 50120 Score: 414 Queries matched: 25 emPAI: 1.20<br>Tubulin alpha-1B chain OS=Rattus norvegicus GN=Tuba1b PE=1 SV=1							
Observed	Mr(expt)	Mr(calc)	Delta	Miss	Score	Expect	Rank	Peptide
586.3047	1755.8923	1755.9559	-0.0637	0	(26)	0.91	1	R.IHFPLATYAPVISAEK.A
586.3097	1755.9073	1755.9559	-0.0487	0	(21)	2.4	1	R.IHFPLATYAPVISAEK.A
878.9676	1755.9206	1755.9559	-0.0353	0	(12)	20	1	R.IHFPLATYAPVISAEK.A
878.9706	1755.9266	1755.9559	-0.0293	0	57	0.00065	1	R.IHFPLATYAPVISAEK.A
878.9723	1755.9300	1755.9559	-0.0259	0	(54)	0.0013	1	R.IHFPLATYAPVISAEK.A
899.9075	1797.8004	1797.8410	-0.0406	0	(9)	50	1	R.NLDIERPTYTNLNR.L + Phospho (ST)
1004.4115	2006.8084	2006.8858	-0.0774	0	58	0.0007	1	K.TIGGGDDSFNTFFSETGAGK.H
803.7223	2408.1451	2408.2012	-0.0562	0	45	0.013	1	R.FDGALNVDLTEFQTNLVPYPR.I

**Spot D33**	TBB5_RAT Mass: 49639 Score: 472 Queries matched: 23 emPAI: 1.76<br>Tubulin beta-5 chain OS=Rattus norvegicus GN=Tubb5 PE=1 SV=1							
Observed	Mr(expt)	Mr(calc)	Delta	Miss	Score	Expect	Rank	Peptide
539.2518	1076.4890	1076.5250	-0.0360	1	(0)	1.9e+02	10	K.IREEYPDR.I
539.2578	1076.5010	1076.5250	-0.0240	1	32	0.12	1	K.IREEYPDR.I
565.7846	1129.5546	1129.5880	-0.0333	0	66	5.2e-05	1	R.FPGQLNADLR.K
572.3077	1142.6008	1142.6270	-0.0262	0	61	0.00019	1	K.LAVNMVPFPR.L
580.3002	1158.5858	1158.6219	-0.0361	0	(59)	0.00031	1	K.LAVNMVPFPR.L + Oxidation (M)
644.3531	1286.6916	1286.7169	-0.0253	1	(40)	0.024	1	R.KLAVNMVPFPR.L + Oxidation (M)
651.3048	1300.5950	1300.6299	-0.0349	0	51	0.0018	1	R.ISVYYNEATGGK.Y
658.3476	1314.6806	1314.7118	-0.0312	1	56	0.00064	1	R.KLAVNMVPFPR.L + Oxidation (M); Formyl (K)
668.3211	1334.6276	1334.6904	-0.0628	0	83	1.3e-06	1	R.IMNTFSVVPSPK.V + Oxidation (M)
808.4097	1614.8048	1614.8287	-0.0239	0	59	0.00039	1	R.AILVDLEPGTMDSVR.S
816.3953	1630.7760	1630.8236	-0.0476	0	(58)	0.0005	1	R.AILVDLEPGTMDSVR.S + Oxidation (M)
818.9049	1635.7952	1635.8232	-0.0279	0	(11)	27	1	R.LHFFMPGFAPLTSR.G + Oxidation (M)
818.9050	1635.7954	1635.8232	-0.0277	0	44	0.012	1	R.LHFFMPGFAPLTSR.G + Oxidation (M)
818.9064	1635.7982	1635.8232	-0.0249	0	(9)	44	1	R.LHFFMPGFAPLTSR.G + Oxidation (M)
546.2742	1635.8008	1635.8232	-0.0224	0	(13)	16	1	R.LHFFMPGFAPLTSR.G + Oxidation (M)
818.9077	1635.8008	1635.8232	-0.0223	0	(32)	0.2	1	R.LHFFMPGFAPLTSR.G + Oxidation (M)
818.9089	1635.8032	1635.8232	-0.0199	0	(32)	0.21	1	R.LHFFMPGFAPLTSR.G + Oxidation (M)
546.2792	1635.8158	1635.8232	-0.0074	0	(10)	35	1	R.LHFFMPGFAPLTSR.G + Oxidation (M)
830.4238	1658.8330	1658.8879	-0.0549	0	(63)	0.00015	1	R.ALTVPELTQQVFDAK.N
830.4240	1658.8334	1658.8879	-0.0545	0	66	8.9e-05	1	R.ALTVPELTQQVFDAK.N
608.2955	1821.8647	1821.9156	-0.0509	0	20	3.6	1	R.EIVHIQAGQCGNQIGAK.F + Carbamidomethyl (C)
979.9829	1957.9512	1957.9745	-0.0233	0	19	4.9	1	K.GHYTEGAELVDSVLDVVR.K
908.7625	2723.2657	2723.3259	-0.0602	0	53	0.0024	1	K.LTTPTYGDLNHLVSATMSGVTTCLR.F<br>+ Carbamidomethyl (C); Oxidation (M)

**Spot D34**	LDHB_RAT Mass: 36589 Score: 278 Queries matched: 19 emPAI: 1.66<br>L-lactate dehydrogenase B chain OS=Rattus norvegicus GN=Ldhb PE=1 SV=2							
Observed	Mr(expt)	Mr(calc)	Delta	Miss	Score	Expect	Rank	Peptide
479.3118	956.6090	956.6059	0.0032	0	19	0.81	1	K.FIIPQIVK.Y
480.2819	958.5492	958.5447	0.0045	0	7	16	1	R.GLTSVINQK.L
506.2876	1010.5606	1010.5583	0.0024	0	16	1.6	1	R.IHPVSTMVK.G
588.8011	1175.5876	1175.5822	0.0054	0	(15)	2.4	1	K.SADTLWDIQK.D
588.8014	1175.5882	1175.5822	0.0060	0	55	0.00027	1	K.SADTLWDIQK.D

Table S6. List of peptide sequences for each protein assignment

**Spot D34**	LDHB_RAT Mass: 36589 Score: 278 Queries matched: 19 emPAI: 1.66<br>L-lactate dehydrogenase B chain OS=Rattus norvegicus GN=Ldhb PE=1 SV=2							
Observed	Mr(expt)	Mr(calc)	Delta	Miss	Score	Expect	Rank	Peptide
593.8290	1185.6434	1185.6353	0.0081	1	70	8.4e-06	1	K.LKDDEVAQLR.K
614.8656	1227.7166	1227.7187	-0.0020	1	30	0.076	1	R.GLTSVINQKLK.D + Formyl (K)
627.3234	1252.6322	1252.6373	-0.0051	0	(4)	29	1	K.MVVDSAYEVIK.L
627.3242	1252.6338	1252.6373	-0.0035	0	(9)	11	1	K.MVVDSAYEVIK.L
635.3257	1268.6368	1268.6322	0.0046	0	56	0.00023	1	K.MVVDSAYEVIK.L + Oxidation (M)
504.2602	1509.7588	1509.7675	-0.0087	1	(36)	0.022	1	K.IVADKDYSVTANSK.I
755.8928	1509.7710	1509.7675	0.0036	1	41	0.0071	1	K.IVADKDYSVTANSK.I
761.9191	1521.8236	1521.8112	0.0124	1	35	0.025	1	K.MVVDSAYEVIKLK.G + Formyl (K)
815.4345	1628.8544	1628.8509	0.0036	0	(33)	0.04	1	K.SLADELALVDVLEDK.L
815.4393	1628.8640	1628.8509	0.0132	0	84	3.5e-07	1	K.SLADELALVDVLEDK.L
833.9373	1665.8600	1665.8573	0.0027	0	36	0.019	1	K.LIAPVADDETAVPNNK.I
873.9201	1745.8256	1745.8237	0.0020	0	(10)	9.7	1	K.LIAPVADDETAVPNNK.I + Phospho (ST)
644.6516	1930.9330	1930.9281	0.0049	0	61	6.3e-05	1	K.GEMMDLQHGSLFLQTPK.I
655.3134	1962.9184	1962.9179	0.0004	0	(24)	0.31	1	K.GEMMDLQHGSLFLQTPK.I + 2 Oxidation (M)

**Spot D35**	KPYM_RAT Mass: 57781 Score: 239 Queries matched: 8 emPAI: 0.55<br>Pyruvate kinase isozymes M1/M2 OS=Rattus norvegicus GN=Pkm2 PE=1 SV=3							
Observed	Mr(expt)	Mr(calc)	Delta	Miss	Score	Expect	Rank	Peptide
571.3131	1140.6116	1140.6026	0.0090	0	28	0.11	1	R.GDLGIEIPAEK.V
599.2990	1196.5834	1196.5747	0.0088	0	35	0.022	1	K.ITLDNAYMEK.C
818.9535	1635.8924	1635.8832	0.0093	0	69	1.1e-05	1	K.GVNLPGAAVDLPAVSEK.D
930.4553	1858.8960	1858.8924	0.0037	0	(24)	0.32	1	K.FGVEQDVDMVFASFIR.K
930.4619	1858.9092	1858.8924	0.0169	0	66	2.3e-05	1	K.FGVEQDVDMVFASFIR.K
625.9692	1874.8858	1874.8873	-0.0015	0	(30)	0.089	1	K.FGVEQDVDMVFASFIR.K + Oxidation (M)
938.4555	1874.8964	1874.8873	0.0091	0	(45)	0.0025	1	K.FGVEQDVDMVFASFIR.K + Oxidation (M)
812.4314	2434.2724	2434.2744	-0.0020	0	89	9.3e-08	1	R.AATESFASDPILYRPVAVALDTK.G

**Spot D36**	RLA0_RAT Mass: 34194 Score: 197 Queries matched: 10 emPAI: 0.52<br>60S acidic ribosomal protein P0 OS=Rattus norvegicus GN=Rplp0 PE=1 SV=2							
Observed	Mr(expt)	Mr(calc)	Delta	Miss	Score	Expect	Rank	Peptide
609.3465	1216.6784	1216.6703	0.0081	0	56	0.00018	1	K.IIQLLDDYPK.C
657.3597	1312.7048	1312.7027	0.0021	0	(35)	0.025	1	K.TSFFQALGITTK.I
657.3630	1312.7114	1312.7027	0.0087	0	(26)	0.22	1	K.TSFFQALGITTK.I
657.3634	1312.7122	1312.7027	0.0095	0	(27)	0.16	1	K.TSFFQALGITTK.I
657.3642	1312.7138	1312.7027	0.0111	0	87	1.5e-07	1	K.TSFFQALGITTK.I
707.3830	1412.7514	1412.7446	0.0069	1	19	0.93	1	R.DMLLANKVPAAAR.A + Oxidation (M); Formyl (K)
714.9218	1427.8290	1427.8235	0.0055	0	(13)	4.1	1	R.GTIEILSDVQLIK.T
714.9246	1427.8346	1427.8235	0.0111	0	54	0.00027	1	R.GTIEILSDVQLIK.T
918.3483	1834.6820	1834.6727	0.0093	0	2	51	1	K.EESEESDEDMGFGLFD.-
948.5129	1895.0112	1894.9928	0.0185	0	52	0.00053	1	R.VLALSVETDYTFPLAEK.V

**Spot D37**	ALDR_RAT Mass: 35774 Score: 346 Queries matched: 13 emPAI: 1.46<br>Aldose reductase OS=Rattus norvegicus GN=Akr1b1 PE=1 SV=3							
Observed	Mr(expt)	Mr(calc)	Delta	Miss	Score	Expect	Rank	Peptide
462.7359	923.4572	923.4535	0.0038	0	43	0.0032	1	K.VAIDMGYR.H
475.2733	948.5320	948.5280	0.0040	0	30	0.08	1	R.QDLFIVSK.L

Table S6. List of peptide sequences for each protein assignment

**Spot D37**	ALDR_RAT Mass: 35774 Score: 346 Queries matched: 13 emPAI: 1.46<br>Aldose reductase OS=Rattus norvegicus GN=Akr1b1 PE=1 SV=3							
Observed	Mr(expt)	Mr(calc)	Delta	Miss	Score	Expect	Rank	Peptide
552.3020	1102.5894	1102.5845	0.0049	0	(54)	0.0003	1	K.MPTLGLGTWK.S
552.3032	1102.5918	1102.5845	0.0073	0	(74)	2.9e-06	1	K.MPTLGLGTWK.S
553.3242	1104.6338	1104.6291	0.0047	1	(15)	2.7	1	K.RQDLFIVSK.L
553.3261	1104.6376	1104.6291	0.0085	1	45	0.0022	1	K.RQDLFIVSK.L
560.2998	1118.5850	1118.5794	0.0056	0	77	1.7e-06	1	K.MPTLGLGTWK.S + Oxidation (M)
711.4312	1420.8478	1420.8402	0.0077	1	31	0.058	1	R.NLVVIPKSVTPAR.I + Formyl (K)
779.4173	1556.8200	1556.8311	-0.0110	0	107	1.7e-09	1	K.AIGVSNFNPLQIER.I
779.4274	1556.8402	1556.8311	0.0092	0	(67)	1.5e-05	1	K.AIGVSNFNPLQIER.I
819.4002	1636.7858	1636.7974	-0.0115	0	(36)	0.022	1	K.AIGVSNFNPLQIER.I + Phospho (ST)
1132.5367	2263.0588	2263.0467	0.0122	0	41	0.0064	1	K.VFDFELSNEDMATLLSYNR.N
1172.5212	2343.0278	2343.0130	0.0148	0	(13)	4.6	1	K.VFDFELSNEDMATLLSYNR.N + Phospho (ST)
**Spot D38**	GBB1_RAT Mass: 37353 Score: 138 Queries matched: 5 emPAI: 0.47<br>Guanine nucleotide-binding protein G(I)/G(S)/G(T) subunit beta-1 OS=Rattus norvegicus GN=Gnb1 PE=1 SV=4							
Observed	Mr(expt)	Mr(calc)	Delta	Miss	Score	Expect	Rank	Peptide
668.8138	1335.6130	1335.6030	0.0101	0	48	0.0013	1	K.IYAMHWGTDSR.L
451.5391	1351.5955	1351.5979	-0.0024	0	(34)	0.034	1	K.IYAMHWGTDSR.L + Oxidation (M)
676.8107	1351.6068	1351.5979	0.0089	0	(36)	0.021	1	K.IYAMHWGTDSR.L + Oxidation (M)
676.8109	1351.6072	1351.5979	0.0093	0	(33)	0.037	1	K.IYAMHWGTDSR.L + Oxidation (M)
677.3590	1352.7034	1352.6976	0.0058	0	74	3e-06	1	K.LIIWDSYTTNK.V
**Spot D39**	HSP7C_RAT Mass: 70827 Score: 178 Queries matched: 16 emPAI: 0.29<br>Heat shock cognate 71 kDa protein OS=Rattus norvegicus GN=Hspa8 PE=1 SV=1							
Observed	Mr(expt)	Mr(calc)	Delta	Miss	Score	Expect	Rank	Peptide
417.7073	833.4000	833.3953	0.0048	0	4	27	2	R.MVQEAEK.Y
599.3373	1196.6600	1196.6553	0.0047	0	26	0.2	1	K.FELTGIPPAPR.G
610.8041	1219.5936	1219.5907	0.0030	0	12	5.1	1	K.CNEIISWLDK.N
652.3064	1302.5982	1302.5914	0.0068	0	64	3.5e-05	1	K.NSLESYAFNMK.A
652.3068	1302.5990	1302.5914	0.0076	0	(29)	0.11	1	K.NSLESYAFNMK.A
660.3021	1318.5896	1318.5863	0.0033	0	(3)	37	1	K.NSLESYAFNMK.A + Oxidation (M)
660.3022	1318.5898	1318.5863	0.0035	0	(59)	9.1e-05	1	K.NSLESYAFNMK.A + Oxidation (M)
660.3024	1318.5902	1318.5863	0.0039	0	(10)	8.4	1	K.NSLESYAFNMK.A + Oxidation (M)
660.3048	1318.5950	1318.5863	0.0087	0	(11)	6.2	1	K.NSLESYAFNMK.A + Oxidation (M)
660.3057	1318.5968	1318.5863	0.0105	0	(16)	2	1	K.NSLESYAFNMK.A + Oxidation (M)
692.3528	1382.6910	1382.5577	0.1333	0	(4)	30	3	K.NSLESYAFNMK.A + Phospho (ST)
582.6060	1744.7962	1744.8016	-0.0054	1	39	0.012	1	K.NQTAEKEEFEHQQK.E
754.0526	2259.1360	2259.1383	-0.0023	0	78	1.4e-06	1	K.SINPDEAVAYGAAVQAAILSGDK.S
1130.5817	2259.1488	2259.1383	0.0106	0	(12)	4.9	1	K.SINPDEAVAYGAAVQAAILSGDK.S
1170.5618	2339.1090	2339.1046	0.0045	0	(22)	0.55	1	K.SINPDEAVAYGAAVQAAILSGDK.S + Phospho (ST)
1116.1672	3345.4798	3345.4919	-0.0121	0	10	8.1	1	K.LYQSAGGMPGGMPGGFPGGGAPPSGGASSGPTIEEVD.-
**Spot D40**	RSSA_RAT Mass: 32803 Score: 156 Queries matched: 6 emPAI: 0.39<br>40S ribosomal protein SA OS=Rattus norvegicus GN=Rpsa PE=1 SV=3							
Observed	Mr(expt)	Mr(calc)	Delta	Miss	Score	Expect	Rank	Peptide
456.7798	911.5450	911.5440	0.0010	0	21	1.3	1	R.LLVVTDPR.A
602.3313	1202.6480	1202.6408	0.0073	0	44	0.0087	1	K.FAAATGATPIAGR.F

Table S6. List of peptide sequences for each protein assignment

**Spot D40**	RSSA_RAT Mass: 32803 Score: 156 Queries matched: 6 emPAI: 0.39<br>40S ribosomal protein SA OS=Rattus norvegicus GN=Rpsa PE=1 SV=3							
Observed	Mr(expt)	Mr(calc)	Delta	Miss	Score	Expect	Rank	Peptide
653.8303	1305.6460	1305.6387	0.0073	0	4	91	9	R.YVDIAIPCNNK.G + Carbamidomethyl (C)
654.3245	1306.6344	1306.6227	0.0117	0	(3)	1.4e+02	1	R.YVDIAIPCNNK.G + Carbamidomethyl (C); Deamidated (NQ)
849.9393	1697.8640	1697.8526	0.0115	0	83	1.9e-06	1	R.FTPGTFTNQIQAAFR.E
870.9830	1739.9514	1739.9417	0.0097	0	74	1.3e-05	1	R.AIVAIENPADVSVISSR.N
**Spot D41**	PIR_RAT Mass: 32158 Score: 113 Queries matched: 7 emPAI: 0.74<br>Pirin OS=Rattus norvegicus GN=Pir PE=1 SV=1							
Observed	Mr(expt)	Mr(calc)	Delta	Miss	Score	Expect	Rank	Peptide
437.7714	873.5282	873.5284	-0.0001	0	27	0.13	1	K.VTLSVLSR.E
501.8210	1001.6274	1001.6233	0.0041	1	14	2.9	1	K.KVTLSVLSR.E
549.2994	1096.5842	1096.5805	0.0038	0	47	0.0014	1	R.TPTLYLDFK.L
632.8212	1263.6278	1263.6169	0.0109	0	22	0.49	1	K.MVEPQYQELK.S
746.8419	1491.6692	1491.6599	0.0094	0	47	0.0018	1	K.MNPGDLQWMTAGR.G + Oxidation (M)
746.8443	1491.6740	1491.6599	0.0142	0	(19)	1.2	1	K.MNPGDLQWMTAGR.G + Oxidation (M)
764.9381	1527.8616	1527.8508	0.0108	0	52	0.0005	1	K.DGVTVAVISGEALGIK.S
**Spot D42**	IMPA2_RAT Mass: 31776 Score: 64 Queries matched: 2 emPAI: 0.25<br>Inositol monophosphatase 2 OS=Rattus norvegicus GN=Impa2 PE=2 SV=1							
Observed	Mr(expt)	Mr(calc)	Delta	Miss	Score	Expect	Rank	Peptide
520.8226	1039.6306	1039.6277	0.0029	0	41	0.0063	1	K.ALVLTEIGPK.R
586.8333	1171.6520	1171.6448	0.0072	0	52	0.00046	1	R.VEDLIVSELR.K
**Spot D43**	PSA3_RAT Mass: 28401 Score: 324 Queries matched: 18 emPAI: 2.96<br>Proteasome subunit alpha type-3 OS=Rattus norvegicus GN=Psma3 PE=1 SV=3							
Observed	Mr(expt)	Mr(calc)	Delta	Miss	Score	Expect	Rank	Peptide
460.2633	918.5120	918.5035	0.0085	1	15	2.6	1	K.RLFNVDR.H
475.2602	948.5058	948.4917	0.0142	0	24	0.27	1	K.DGVVFGVEK.L
548.2737	1094.5328	1094.5356	-0.0028	1	29	0.093	1	K.LYEEGSNKR.L
548.2795	1094.5444	1094.5356	0.0088	1	(10)	7.2	2	K.LYEEGSNKR.L
557.7880	1113.5614	1113.5529	0.0086	0	(29)	0.1	1	R.VFQVEYAMK.A
565.7845	1129.5544	1129.5478	0.0067	0	35	0.026	1	R.VFQVEYAMK.A + Oxidation (M)
576.8080	1151.6014	1151.5975	0.0040	0	(34)	0.029	1	R.SNFGYNIPLK.H
576.8107	1151.6068	1151.5975	0.0094	0	69	9.9e-06	1	R.SNFGYNIPLK.H
609.3288	1216.6430	1216.6411	0.0019	0	72	5.6e-06	1	K.AVENSSTAIGIR.C
410.2322	1227.6748	1227.6863	-0.0115	0	(29)	0.095	1	K.IIYIVHDEVK.D
614.8532	1227.6918	1227.6863	0.0055	0	31	0.063	1	K.IIYIVHDEVK.D
614.8534	1227.6922	1227.6863	0.0059	0	(26)	0.21	1	K.IIYIVHDEVK.D
690.8786	1379.7426	1379.7343	0.0083	0	(67)	1.7e-05	1	R.HVGMAVAGLLADAR.S
690.8811	1379.7476	1379.7343	0.0133	0	(16)	2	1	R.HVGMAVAGLLADAR.S
698.8756	1395.7366	1395.7293	0.0074	0	96	2.2e-08	1	R.HVGMAVAGLLADAR.S + Oxidation (M)
491.2739	1470.7999	1470.8082	-0.0083	1	55	0.00024	1	K.IIYIVHDEVKDK.A
736.4145	1470.8144	1470.8082	0.0062	1	(40)	0.0073	1	K.IIYIVHDEVKDK.A
868.3012	1734.5878	1734.5815	0.0064	1	13	4.3	1	K.ESLKEEDESDDDNM.- + Phospho (ST)

Table S6. List of peptide sequences for each protein assignment

**Spot D44**	PGAM1_RAT Mass: 28814 Score: 564 Queries matched: 35 emPAI: 2.88<br>Phosphoglycerate mutase 1 OS=Rattus norvegicus GN=Pgam1 PE=1 SV=4							
Observed	Mr(expt)	Mr(calc)	Delta	Miss	Score	Expect	Rank	Peptide
488.2504	974.4862	974.4855	0.0008	0	8	33	1	K.AMEAVAAQGK.V
530.2824	1058.5502	1058.5509	-0.0006	0	(1)	1.6e+02	10	R.HYGGLTGLNK.A
530.2824	1058.5502	1058.5509	-0.0006	0	(1)	1.8e+02	8	R.HYGGLTGLNK.A
530.2834	1058.5522	1058.5509	0.0014	0	(10)	22	1	R.HYGGLTGLNK.A
530.2838	1058.5530	1058.5509	0.0022	0	(12)	14	1	R.HYGGLTGLNK.A
530.2839	1058.5532	1058.5509	0.0024	0	(16)	5.1	1	R.HYGGLTGLNK.A
530.2844	1058.5542	1058.5509	0.0034	0	26	0.53	1	R.HYGGLTGLNK.A
530.2848	1058.5550	1058.5509	0.0042	0	(17)	3.8	1	R.HYGGLTGLNK.A
530.2866	1058.5586	1058.5509	0.0078	0	(1)	1.6e+02	2	R.HYGGLTGLNK.A
552.2990	1102.5834	1102.5804	0.0030	1	(41)	0.016	1	R.KAMEAVAAQGK.V
560.2970	1118.5794	1118.5754	0.0041	1	60	0.00024	1	R.KAMEAVAAQGK.V + Oxidation (M)
575.8378	1149.6610	1149.6618	-0.0008	0	(24)	0.96	1	R.VLIAAHGNSLR.G
575.8392	1149.6638	1149.6618	0.0020	0	(22)	1.2	1	R.VLIAAHGNSLR.G
575.8408	1149.6670	1149.6618	0.0052	0	(50)	0.0021	1	R.VLIAAHGNSLR.G
575.8408	1149.6670	1149.6618	0.0052	0	(12)	14	1	R.VLIAAHGNSLR.G
575.8411	1149.6676	1149.6618	0.0058	0	64	9.4e-05	1	R.VLIAAHGNSLR.G
656.8055	1311.5964	1311.5956	0.0009	0	(24)	0.93	1	R.HGESAWNLENR.F
656.8064	1311.5982	1311.5956	0.0027	0	(33)	0.14	1	R.HGESAWNLENR.F
656.8070	1311.5994	1311.5956	0.0039	0	44	0.0094	1	R.HGESAWNLENR.F
656.8093	1311.6040	1311.5956	0.0085	0	(8)	40	1	R.HGESAWNLENR.F
842.4561	1682.8976	1682.9032	-0.0055	0	(11)	26	1	R.ALPFWNEEIVPQIK.E
842.4634	1682.9122	1682.9032	0.0091	0	68	5.3e-05	1	R.ALPFWNEEIVPQIK.E
934.9388	1867.8630	1867.8509	0.0121	0	47	0.0074	1	R.YADLTEDQLPSCESLK.D + Carbamidomethyl (C)
660.6279	1978.8619	1978.8697	-0.0078	0	(26)	1.1	1	R.FSGWYDADLSPAGHEEAK.R
660.6295	1978.8667	1978.8697	-0.0030	0	(61)	0.00033	1	R.FSGWYDADLSPAGHEEAK.R
660.6320	1978.8742	1978.8697	0.0045	0	90	3.7e-07	1	R.FSGWYDADLSPAGHEEAK.R
990.4490	1978.8834	1978.8697	0.0137	0	(16)	11	1	R.FSGWYDADLSPAGHEEAK.R
1013.5409	2025.0672	2025.0571	0.0101	1	5	1.1e+02	1	R.ALPFWNEEIVPQIKEGK.R + Formyl (K)
806.3726	2416.0960	2416.1045	-0.0086	0	(31)	0.38	1	R.SYDVPPPPMEPDHPFYSNISK.D
806.3749	2416.1029	2416.1045	-0.0017	0	(37)	0.096	1	R.SYDVPPPPMEPDHPFYSNISK.D
809.0561	2424.1465	2424.1478	-0.0014	1	117	9.7e-10	1	R.YADLTEDQLPSCESLKDTIAR.A + Carbamidomethyl (C)
809.0562	2424.1468	2424.1478	-0.0011	1	(112)	2.7e-09	1	R.YADLTEDQLPSCESLKDTIAR.A + Carbamidomethyl (C)
809.0562	2424.1468	2424.1478	-0.0011	1	(25)	1.4	1	R.YADLTEDQLPSCESLKDTIAR.A + Carbamidomethyl (C)
811.7068	2432.0986	2432.0995	-0.0009	0	38	0.07	1	R.SYDVPPPPMEPDHPFYSNISK.D + Oxidation (M)
811.7089	2432.1049	2432.0995	0.0054	0	(14)	20	1	R.SYDVPPPPMEPDHPFYSNISK.D + Oxidation (M)

**Spot U45**	PDC6I_RAT Mass: 96570 Score: 448 Queries matched: 16 emPAI: 0.57<br>Programmed cell death 6-interacting protein OS=Rattus norvegicus GN=Pdcd6ip PE=1 SV=2							
Observed	Mr(expt)	Mr(calc)	Delta	Miss	Score	Expect	Rank	Peptide
426.7360	851.4574	851.4753	-0.0178	0	5	21	9	R.IYGGLTTK.V
450.2206	898.4266	898.4436	-0.0170	0	22	0.4	1	K.FTDLFEK.M
494.7682	987.5218	987.5236	-0.0018	0	20	0.77	1	R.EILEESLR.L
532.7948	1063.5750	1063.5913	-0.0163	0	66	2e-05	1	K.LALASLGYEK.S
569.8116	1137.6086	1137.6142	-0.0055	0	50	0.00066	1	K.HEGALETLLR.Y

Table S6. List of peptide sequences for each protein assignment

**Spot U45**	PDC6I_RAT Mass: 96570 Score: 448 Queries matched: 16 emPAI: 0.57<br>Programmed cell death 6-interacting protein OS=Rattus norvegicus GN=Pdcd6ip PE=1 SV=2							
Observed	Mr(expt)	Mr(calc)	Delta	Miss	Score	Expect	Rank	Peptide
630.3580	1258.7014	1258.7132	-0.0118	1	15	2.4	1	K.SLLSNLDEIKK.E
652.8438	1303.6730	1303.6806	-0.0075	0	(47)	0.0016	1	K.TMQGSEVVNVLK.S
660.8362	1319.6578	1319.6755	-0.0177	0	57	0.00016	1	K.TMQGSEVVNVLK.S + Oxidation (M)
698.8646	1395.7146	1395.7398	-0.0251	0	69	9.8e-06	1	K.FYNELTEILVR.F
765.8619	1529.7092	1529.7150	-0.0058	0	73	4.6e-06	1	K.LANQAADYFGDAFK.Q
766.8324	1531.6502	1531.7002	-0.0499	0	109	9.4e-10	1	R.LLDEEEATDNDLR.A
862.4388	1722.8630	1722.8657	-0.0027	0	29	0.11	1	K.YFYFQEVFPTLAAK.Q
888.4706	1774.9266	1774.9400	-0.0134	0	(49)	0.00097	1	K.MVPVSVQQSLAVFSQR.K
896.4664	1790.9182	1790.9349	-0.0167	0	88	1.5e-07	1	K.MVPVSVQQSLAVFSQR.K + Oxidation (M)
943.5089	1885.0032	1885.0157	-0.0124	0	19	1.1	1	K.STAVVEQGGIQTVDQLIK.E
835.4217	2503.2433	2503.2806	-0.0373	0	48	0.0013	1	K.FLTALAQDGVINEEALSVTELDR.I

**Spot U46**	HS90B_RAT Mass: 83229 Score: 203 Queries matched: 8 emPAI: 0.30<br>Heat shock protein HSP 90-beta OS=Rattus norvegicus GN=Hsp90ab1 PE=1 SV=4							
Observed	Mr(expt)	Mr(calc)	Delta	Miss	Score	Expect	Rank	Peptide
520.2683	1038.5220	1038.4869	0.0351	0	40	0.0078	1	R.YESLTDPSK.L
597.8501	1193.6856	1193.6404	0.0452	0	66	2.1e-05	1	K.IDIIPNPQER.T
621.8731	1241.7316	1241.6979	0.0337	0	30	0.077	1	K.ADLINNLGTIAK.S
638.3391	1274.6636	1274.6354	0.0283	0	67	1.6e-05	1	R.ELISNASDALDK.I
656.3135	1310.6124	1310.5626	0.0498	0	46	0.0019	1	K.EDQTEYLEER.R
683.3941	1364.7736	1364.7221	0.0515	0	81	5.9e-07	1	R.TLTLVDTGIGMTK.A + Oxidation (M)
603.6797	1808.0173	1807.9509	0.0664	0	(4)	27	1	K.HSQFIGYPITLYLEK.E
905.0248	1808.0350	1807.9509	0.0842	0	21	0.49	1	K.HSQFIGYPITLYLEK.E

**Spot U47**	THOP1_RAT Mass: 78335 Score: 385 Queries matched: 20 emPAI: 0.91<br>Thimet oligopeptidase OS=Rattus norvegicus GN=Thop1 PE=1 SV=4							
Observed	Mr(expt)	Mr(calc)	Delta	Miss	Score	Expect	Rank	Peptide
461.2368	920.4590	920.4967	-0.0377	0	19	0.95	1	K.QDAFLLSK.G
552.2694	1102.5242	1102.5883	-0.0641	0	53	0.00035	1	R.QANAGLFNLR.Q
558.7732	1115.5318	1115.5723	-0.0405	0	50	0.00073	1	R.WDLSAQQIR.A
566.8056	1131.5966	1131.6288	-0.0322	1	17	1.6	1	R.FKQEGVLSPK.V
623.3322	1244.6498	1244.6976	-0.0478	0	34	0.033	1	R.ALTTQLIEQTK.C
643.2835	1284.5524	1284.6173	-0.0648	0	30	0.083	1	K.EYFPMQVVTR.G + Oxidation (M)
674.7783	1347.5420	1347.5765	-0.0344	0	31	0.076	1	R.YYMNQVEETR.Y + Oxidation (M)
680.2810	1358.5474	1358.5846	-0.0372	0	48	0.0014	1	K.LSEFDVEMSMR.Q + Oxidation (M)
688.2789	1374.5432	1374.5795	-0.0363	0	(43)	0.0047	1	K.LSEFDVEMSMR.Q + 2 Oxidation (M)
732.8601	1463.7056	1463.7620	-0.0564	0	53	0.0004	1	K.ALADVEVTYTVQR.N
754.8663	1507.7180	1507.7783	-0.0603	0	43	0.0041	1	R.NILDFPQHVSPNK.D
776.3815	1550.7484	1550.7940	-0.0456	0	85	2.8e-07	1	K.TSQTVATFLDELAR.K
776.3819	1550.7492	1550.7940	-0.0448	0	(30)	0.082	1	K.TSQTVATFLDELAR.K
777.8325	1553.6504	1553.6854	-0.0349	0	23	0.46	1	R.THADYVLEMNMAK.T + 2 Oxidation (M)
784.3632	1566.7118	1566.7678	-0.0560	0	64	3e-05	1	K.NLNEDTTFLPFTR.E
816.3673	1630.7200	1630.7604	-0.0403	0	(28)	0.14	1	K.TSQTVATFLDELAR.K + Phospho (ST)
889.9285	1777.8424	1777.8880	-0.0456	0	4	38	1	R.TSILRPGGSEDASTMLK.Q + Oxidation (M)
894.3693	1786.7240	1786.8261	-0.1021	0	57	0.00016	1	R.VGAQDFEDVSYESTLK.A

Table S6. List of peptide sequences for each protein assignment

**Spot U47**	THOP1_RAT Mass: 78335 Score: 385 Queries matched: 20 emPAI: 0.91<br>Thimet oligopeptidase OS=Rattus norvegicus GN=Thop1 PE=1 SV=4							
Observed	Mr(expt)	Mr(calc)	Delta	Miss	Score	Expect	Rank	Peptide
895.4135	1788.8124	1788.8781	-0.0657	0	46	0.0022	1	R.EELGGLPEDFLNSLEK.T
729.3123	2184.9151	2185.0287	-0.1137	0	46	0.0021	1	K.VDQVLHTQTDVDPAEEYAR.L

**Spot U48**	DC1I2_RAT Mass: 71134 Score: 116 Queries matched: 6 emPAI: 0.36<br>Cytoplasmic dynein 1 intermediate chain 2 OS=Rattus norvegicus GN=Dync1i2 PE=1 SV=1							
Observed	Mr(expt)	Mr(calc)	Delta	Miss	Score	Expect	Rank	Peptide
487.7571	973.4996	973.5192	-0.0196	0	51	0.00061	1	R.TLAEINASR.A
536.7924	1071.5702	1071.5713	-0.0011	0	40	0.0083	1	K.ITQVDFPPR.E
427.2038	1278.5896	1278.6092	-0.0196	0	(26)	0.2	1	K.APPHELTEEEK.Q
640.3021	1278.5896	1278.6092	-0.0195	0	34	0.034	1	K.APPHELTEEEK.Q
853.0982	2556.2728	2556.3071	-0.0344	0	47	0.0015	1	R.EIAVGDSEGQIVIYDVGEQIAVPR.N
984.8109	2951.4109	2951.4624	-0.0516	0	35	0.027	1	R.LDLWNLNNDTEVPTASISVEGNPALNR.V

**Spot U49**	HS105_RAT Mass: 96357 Score: 62 Queries matched: 2 emPAI: 0.08<br>Heat shock protein 105 kDa OS=Rattus norvegicus GN=Hsph1 PE=1 SV=1							
Observed	Mr(expt)	Mr(calc)	Delta	Miss	Score	Expect	Rank	Peptide
713.3527	1424.6908	1424.6857	0.0051	0	27	0.17	1	R.DLLNMYIETEGK.M
766.4060	1530.7974	1530.7889	0.0085	0	59	9.2e-05	1	K.TEEVSAIEIVGGATR.I

**Spot U50**	TBA1A_RAT Mass: 50104 Score: 361 Queries matched: 21 emPAI: 1.74<br>Tubulin alpha-1A chain OS=Rattus norvegicus GN=Tuba1a PE=1 SV=1							
Observed	Mr(expt)	Mr(calc)	Delta	Miss	Score	Expect	Rank	Peptide
452.2231	902.4316	902.4208	0.0109	0	16	1.9	1	K.FDLMYAK.R + Oxidation (M)
508.2831	1014.5516	1014.5709	-0.0193	0	31	0.06	1	K.DVNAAIATIK.T
534.3250	1066.6354	1066.6022	0.0332	0	(22)	0.41	1	K.EIIDLVLDR.I + Glu->pyro-Glu (N-term E)
543.3032	1084.5918	1084.6128	-0.0210	0	50	0.00071	1	K.EIIDLVLDR.I
697.3917	1392.7688	1392.7402	0.0287	0	(33)	0.035	1	R.QLFHPEQLITGK.E + Gln->pyro-Glu (N-term Q)
697.3962	1392.7778	1392.7402	0.0377	0	(0)	72	1	R.QLFHPEQLITGK.E + Gln->pyro-Glu (N-term Q)
470.9415	1409.8027	1409.7667	0.0360	0	(1)	56	1	R.QLFHPEQLITGK.E
705.9116	1409.8086	1409.7667	0.0420	0	43	0.0032	1	R.QLFHPEQLITGK.E
729.4584	1456.9022	1456.8613	0.0409	0	65	1.8e-05	1	R.LIGQIVSSITASLR.F
729.4599	1456.9052	1456.8613	0.0439	0	(25)	0.18	1	R.LIGQIVSSITASLR.F
745.8862	1489.7578	1489.7330	0.0248	0	(21)	0.7	1	R.QLFHPEQLITGK.E + Phospho (ST)
769.4378	1536.8610	1536.8276	0.0334	0	(4)	27	1	R.LIGQIVSSITASLR.F + Phospho (ST)
851.4603	1700.9060	1700.8985	0.0075	0	113	3.9e-10	1	R.AVFVDLEPTVIDEVR.T
573.6356	1717.8850	1717.8747	0.0103	0	(60)	8.9e-05	1	R.NLDIERPTYTNLNR.L
859.9548	1717.8950	1717.8747	0.0203	0	83	4.2e-07	1	R.NLDIERPTYTNLNR.L
586.3367	1755.9883	1755.9559	0.0323	0	(26)	0.2	1	R.IHFPLATYAPVISAEK.A
879.0048	1755.9950	1755.9559	0.0391	0	45	0.0023	1	R.IHFPLATYAPVISAEK.A
899.9471	1797.8796	1797.8410	0.0386	0	(19)	0.99	1	R.NLDIERPTYTNLNR.L + Phospho (ST)
912.9537	1823.8928	1823.9782	-0.0853	0	24	0.37	1	K.VGINYQPPTVVPGGDLAK.V
1004.4675	2006.9204	2006.8858	0.0346	0	55	0.00025	1	K.TIGGGDDSFNTFFSETGAGK.H
1118.6317	2235.2488	2235.2012	0.0477	1	4	23	1	K.VGINYQPPTVVPGGDLAKVQR.A + Formyl (K)

Table S6. List of peptide sequences for each protein assignment

Spot U51	TBA1A_RAT Mass: 50104 Score: 331 Queries matched: 14 emPAI: 1.05<br>Tubulin alpha-1A chain OS=Rattus norvegicus GN=Tuba1a PE=1 SV=1							
Observed	Mr(expt)	Mr(calc)	Delta	Miss	Score	Expect	Rank	Peptide
543.3203	1084.6260	1084.6128	0.0132	0	42	0.0049	1	K.EIIDLVLDR.I
697.3954	1392.7762	1392.7402	0.0361	0	43	0.0038	1	R.QLFHPEQLITGK.E + Gln->pyro-Glu (N-term Q)
470.9434	1409.8084	1409.7667	0.0417	0	(16)	1.7	1	R.QLFHPEQLITGK.E
705.9119	1409.8092	1409.7667	0.0426	0	(41)	0.0052	1	R.QLFHPEQLITGK.E
729.4572	1456.8998	1456.8613	0.0385	0	48	0.00099	1	R.LIGQIVSSITASLR.F
729.4573	1456.9000	1456.8613	0.0387	0	(15)	2	1	R.LIGQIVSSITASLR.F
851.4513	1700.8880	1700.8985	-0.0105	0	109	9.8e-10	1	R.AVFVDLEPTVIDEVR.T
859.9600	1717.9054	1717.8747	0.0307	0	(70)	7.5e-06	1	R.NLDIERPTYTNLNR.L
573.6430	1717.9072	1717.8747	0.0325	0	76	2e-06	1	R.NLDIERPTYTNLNR.L
879.0103	1756.0060	1755.9559	0.0501	0	45	0.0021	1	R.IHFPLATYAPVISAEK.A
586.3427	1756.0063	1755.9559	0.0503	0	(27)	0.15	1	R.IHFPLATYAPVISAEK.A
913.0189	1824.0232	1823.9782	0.0451	0	24	0.3	1	K.VGINYQPPTVVPGGDLAK.V
918.9869	1835.9592	1835.9223	0.0370	0	(21)	0.72	1	R.IHFPLATYAPVISAEK.A + Phospho (ST)
1004.4649	2006.9152	2006.8858	0.0294	0	51	0.00072	1	K.TIGGGDDSFNTFFSETGAGK.H

Spot U52	ODO2_RAT Mass: 48894 Score: 308 Queries matched: 12 emPAI: 1.09<br>Dihydrolipoyllysine-residue succinyltransferase component of 2-oxoglutarate dehydrogenase complex, mitochondrial<br>OS=Rattus norvegicus GN=Dlst PE=1 SV=2							
Observed	Mr(expt)	Mr(calc)	Delta	Miss	Score	Expect	Rank	Peptide
418.2463	834.4780	834.4599	0.0181	0	41	0.0043	1	R.EAVTFLR.K
426.7911	851.5676	851.5593	0.0084	0	33	0.03	1	R.GLVVPVIR.N
452.2453	902.4760	902.4709	0.0052	0	19	1	1	R.TINELGEK.A
508.2728	1014.5310	1014.5208	0.0102	0	35	0.022	1	K.LGFMSAFVK.A + Oxidation (M)
595.3290	1188.6434	1188.6503	-0.0068	0	63	3.7e-05	1	K.VEGGTPLFTLR.K
727.8437	1453.6728	1453.6507	0.0221	0	69	1.1e-05	1	R.NVETMNYADIER.T
735.8328	1469.6510	1469.6456	0.0054	0	(67)	1.9e-05	1	R.NVETMNYADIER.T + Oxidation (M)
735.8329	1469.6512	1469.6456	0.0056	0	(69)	1.1e-05	1	R.NVETMNYADIER.T + Oxidation (M)
1094.0700	2186.1254	2186.1219	0.0035	0	37	0.017	1	K.ASAFALQEQPVVNAVIDDATK.E
729.7194	2186.1364	2186.1219	0.0145	0	(27)	0.15	1	K.ASAFALQEQPVVNAVIDDATK.E
1124.5637	2247.1128	2247.1019	0.0109	0	56	0.00019	1	K.NDVITVQTPAFAESVTEGDVR.W
750.0469	2247.1189	2247.1019	0.0169	0	(32)	0.052	1	K.NDVITVQTPAFAESVTEGDVR.W

Spot U53	GPDA_RAT Mass: 37428 Score: 48 Queries matched: 4 emPAI: 0.33<br>Glycerol-3-phosphate dehydrogenase [NAD+], cytoplasmic OS=Rattus norvegicus GN=Gpd1 PE=1 SV=4							
Observed	Mr(expt)	Mr(calc)	Delta	Miss	Score	Expect	Rank	Peptide
515.3281	1028.6416	1028.6230	0.0187	0	19	0.97	1	K.ANTIGISLIK.G
543.3188	1084.6230	1084.5957	0.0273	0	3	40	2	K.FPLFTAVYK.V
628.3408	1254.6670	1254.6352	0.0319	0	46	0.0019	1	R.LGLMEMIAFAK.L + 2 Oxidation (M)
777.8837	1553.7528	1553.7184	0.0344	0	27	0.16	1	R.VTMWVFEEDIGGR.K + Oxidation (M)

Spot U54	HS90B_RAT Mass: 83229 Score: 133 Queries matched: 6 emPAI: 0.24<br>Heat shock protein HSP 90-beta OS=Rattus norvegicus GN=Hsp90ab1 PE=1 SV=4							
Observed	Mr(expt)	Mr(calc)	Delta	Miss	Score	Expect	Rank	Peptide
523.2811	1044.5476	1044.5927	-0.0451	1	7	16	3	K.VEKVTISNR.L
597.8384	1193.6622	1193.6404	0.0218	0	49	0.00093	1	K.IDIIPNPQER.T
621.8576	1241.7006	1241.6979	0.0027	0	42	0.0051	1	K.ADLINNLGTIAK.S

Table S6. List of peptide sequences for each protein assignment

**Spot U54**	HS90B_RAT Mass: 83229 Score: 133 Queries matched: 6 emPAI: 0.24<br>Heat shock protein HSP 90-beta OS=Rattus norvegicus GN=Hsp90ab1 PE=1 SV=4							
Observed	Mr(expt)	Mr(calc)	Delta	Miss	Score	Expect	Rank	Peptide
638.3320	1274.6494	1274.6354	0.0141	0	67	1.6e-05	1	R.ELISNASDALDK.I
656.2999	1310.5852	1310.5626	0.0226	0	47	0.0016	1	K.EDQTEYLEER.R
683.3765	1364.7384	1364.7221	0.0163	0	15	2.5	1	R.TLTLVDTGIGMTK.A + Oxidation (M)

**Spot U55**	HS90B_RAT Mass: 83229 Score: 313 Queries matched: 16 emPAI: 0.48<br>Heat shock protein HSP 90-beta OS=Rattus norvegicus GN=Hsp90ab1 PE=1 SV=4							
Observed	Mr(expt)	Mr(calc)	Delta	Miss	Score	Expect	Rank	Peptide
571.2537	1140.4928	1140.5523	-0.0595	0	13	3.7	1	K.LGIHEDSTNR.R
576.2492	1150.4838	1150.5506	-0.0667	0	39	0.011	1	K.YIDQEELNK.T
580.7598	1159.5050	1159.5761	-0.0710	0	32	0.058	1	K.SIYYITGESK.E
597.7853	1193.5560	1193.6404	-0.0844	0	66	2.2e-05	1	K.IDIIPNPQER.T
618.7863	1235.5580	1235.6299	-0.0718	1	12	5.3	1	R.RAPFDLFENK.K
621.8184	1241.6222	1241.6979	-0.0757	0	41	0.0067	1	K.ADLINNLGTIAK.S
625.2707	1248.5268	1248.6098	-0.0830	0	44	0.0032	1	K.EQVANSAFVER.V
638.2849	1274.5552	1274.6354	-0.0801	0	66	1.8e-05	1	R.ELISNASDALDK.I
656.2494	1310.4842	1310.5626	-0.0784	0	46	0.0018	1	K.EDQTEYLEER.R
675.3188	1348.6230	1348.7272	-0.1042	0	78	1.2e-06	1	R.TLTLVDTGIGMTK.A
708.7826	1415.5506	1415.6303	-0.0797	0	19	1.1	1	K.EGLELPEDEEEK.K
757.3513	1512.6880	1512.7784	-0.0903	0	87	1.8e-07	1	R.GVVDSEDLPLNISR.E
764.3298	1526.6450	1526.7365	-0.0915	0	9	9.2	1	K.SLTNDWEDHLAVK.H
797.3293	1592.6440	1592.7447	-0.1007	0	(4)	39	1	R.GVVDSEDLPLNISR.E + Phospho (ST)
924.3403	1846.6660	1846.7897	-0.1237	0	47	0.0017	1	R.NPDDITQEEYGEFYK.S
726.2707	2175.7903	2175.9379	-0.1476	0	19	0.95	1	R.YHTSQSGDEMTSLSEYVSR.M

**Spot U56**	1433B_RAT Mass: 28037 Score: 238 Queries matched: 11 emPAI: 2.55<br>14-3-3 protein beta/alpha OS=Rattus norvegicus GN=Ywhab PE=1 SV=3							
Observed	Mr(expt)	Mr(calc)	Delta	Miss	Score	Expect	Rank	Peptide
454.2520	906.4894	906.5174	-0.0280	0	19	0.87	1	R.NLLSVAYK.N
591.7796	1181.5446	1181.5564	-0.0118	0	55	0.00027	1	R.YLSEVASGDNK.Q
595.3164	1188.6182	1188.6536	-0.0354	0	(45)	0.0028	1	K.DSTLIMQLLR.D
603.2985	1204.5824	1204.6485	-0.0661	0	56	0.0002	1	K.DSTLIMQLLR.D + Oxidation (M)
680.3540	1358.6934	1358.7194	-0.0259	0	70	8e-06	1	K.YLILNATHAESK.V
720.3335	1438.6524	1438.6857	-0.0333	0	(8)	12	1	K.YLILNATHAESK.V + Phospho (ST)
766.4127	1530.8108	1530.8518	-0.0410	1	67	1.5e-05	1	R.NLLSVAYKNVVGAR.R + Formyl (K)
799.8660	1597.7174	1597.7332	-0.0158	0	42	0.0052	1	K.AVTEQGHELSNEER.N
839.8474	1677.6802	1677.6995	-0.0193	0	(34)	0.037	1	K.AVTEQGHELSNEER.N + Phospho (ST)
1079.9570	2157.8994	2157.9953	-0.0959	0	48	0.0016	1	K.TAFDEAIAELDTLNEESYK.D
1079.9800	2157.9454	2158.0178	-0.0724	0	39	0.012	1	K.QTTVSNSQQAYQEAFEISK.K

**Spot U57**	1433T_RAT Mass: 27761 Score: 205 Queries matched: 8 emPAI: 1.79<br>14-3-3 protein theta OS=Rattus norvegicus GN=Ywhaq PE=1 SV=1							
Observed	Mr(expt)	Mr(calc)	Delta	Miss	Score	Expect	Rank	Peptide
454.2623	906.5100	906.5174	-0.0074	0	21	0.62	1	R.NLLSVAYK.N
595.3348	1188.6550	1188.6536	0.0014	0	(47)	0.0018	1	K.DSTLIMQLLR.D
603.3367	1204.6588	1204.6485	0.0103	0	55	0.00026	1	K.DSTLIMQLLR.D + Oxidation (M)
660.8378	1319.6610	1319.6721	-0.0110	0	56	0.00019	1	K.YLIANATNPESK.V

Table S6. List of peptide sequences for each protein assignment

**Spot U57**	1433T_RAT Mass: 27761 Score: 205 Queries matched: 8 emPAI: 1.79<br>14-3-3 protein theta OS=Rattus norvegicus GN=Ywhaq PE=1 SV=1							
Observed	Mr(expt)	Mr(calc)	Delta	Miss	Score	Expect	Rank	Peptide
707.8676	1413.7206	1413.6826	0.0380	0	40	0.008	1	R.SICTTVLELLDK.Y + Phospho (ST)
766.8508	1531.6870	1531.7114	-0.0243	0	100	9e-09	1	K.AVTEQGAELSNEER.N
1014.9833	2027.9520	2027.9436	0.0085	0	34	0.031	1	K.QTIENSQGAYQEAFDISK.K
1072.9917	2143.9688	2143.9797	-0.0108	0	36	0.024	1	K.TAFDEAIAELDTLNEDSYK.D

**Spot U58**	1433E_RAT Mass: 29155 Score: 317 Queries matched: 22 emPAI: 2.39<br>14-3-3 protein epsilon OS=Rattus norvegicus GN=Ywhae PE=1 SV=1							
Observed	Mr(expt)	Mr(calc)	Delta	Miss	Score	Expect	Rank	Peptide
454.2751	906.5356	906.5174	0.0182	0	17	1.5	1	R.NLLSVAYK.N
459.2706	916.5266	916.5229	0.0037	0	22	0.46	1	R.IISSIEQK.E
483.2512	964.4878	964.4865	0.0013	0	14	2.6	1	R.EDLVYQAK.L
497.2503	992.4860	992.4848	0.0012	0	20	0.74	1	R.QMVETELK.L + Oxidation (M)
595.3367	1188.6588	1188.6536	0.0052	0	(28)	0.11	1	K.DSTLIMQLLR.D
597.7849	1193.5552	1193.5928	-0.0375	0	55	0.00024	1	K.EAAENSLVAYK.A
603.3376	1204.6606	1204.6485	0.0121	0	(3)	39	1	K.DSTLIMQLLR.D + Oxidation (M)
603.3381	1204.6616	1204.6485	0.0131	0	50	0.00089	1	K.DSTLIMQLLR.D + Oxidation (M)
619.3345	1236.6544	1236.6462	0.0082	0	56	0.00019	1	K.HLIPAANTGESK.V
628.7966	1255.5786	1255.5833	-0.0046	0	(43)	0.0042	1	R.YLAEFATGNDR.K
628.8018	1255.5890	1255.5833	0.0057	0	62	5.3e-05	1	R.YLAEFATGNDR.K
462.2332	1383.6778	1383.6783	-0.0005	1	(8)	12	1	R.YLAEFATGNDRK.E
692.8505	1383.6864	1383.6783	0.0082	1	18	1.3	1	R.YLAEFATGNDRK.E
732.3543	1462.6940	1462.6974	-0.0033	0	(43)	0.0042	1	K.VAGMDVELTVEER.N + Oxidation (M)
732.3550	1462.6954	1462.6974	-0.0019	0	107	1.6e-09	1	K.VAGMDVELTVEER.N + Oxidation (M)
810.4097	1618.8048	1618.7872	0.0176	1	32	0.061	1	K.KVAGMDVELTVEER.N + Oxidation (M); Formyl (K)
895.9545	1789.8944	1789.8846	0.0098	1	25	0.27	1	R.EDLVYQAKLAEQAER.Y + Formyl (K)
612.6458	1834.9156	1834.9247	-0.0091	0	(22)	0.48	1	K.AASDIAMTELPPTHPIR.L + Oxidation (M)
918.4778	1834.9410	1834.9247	0.0163	0	70	7.6e-06	1	K.AASDIAMTELPPTHPIR.L + Oxidation (M)
958.4536	1914.8926	1914.8910	0.0016	0	(39)	0.011	1	K.AASDIAMTELPPTHPIR.L + Oxidation (M); Phospho (ST)
696.6575	2086.9507	2086.9582	-0.0075	0	16	2	1	K.AAFDDAIAELDTLSEESYK.D
1044.4976	2086.9806	2086.9582	0.0224	0	(1)	59	1	K.AAFDDAIAELDTLSEESYK.D
